# Application of Titanium Carbide MXenes in Chemiresistive Gas Sensors

**DOI:** 10.3390/nano13050850

**Published:** 2023-02-24

**Authors:** Elizaveta P. Simonenko, Nikolay P. Simonenko, Artem S. Mokrushin, Tatiana L. Simonenko, Philipp Yu. Gorobtsov, Ilya A. Nagornov, Ghenadii Korotcenkov, Victor V. Sysoev, Nikolay T. Kuznetsov

**Affiliations:** 1 Kurnakov Institute of General and Inorganic Chemistry of the Russian Academy of Sciences, 31 Leninsky pr., 119991 Moscow, Russia; 2 Department of Physics and Engineering, Moldova State University, 2009 Chisinau, Moldova; 3 Department of Physics, Yuri Gagarin State Technical University of Saratov, 77 Polytechnicheskaya str., 410054 Saratov, Russia

**Keywords:** MXene, chemiresistor, Ti_2_C, Ti_3_C_2_, nanocomposite, synthesis, gas-sensing mechanism, review

## Abstract

The titanium carbide MXenes currently attract an extreme amount of interest from the material science community due to their promising functional properties arising from the two-dimensionality of these layered structures. In particular, the interaction between MXene and gaseous molecules, even at the physisorption level, yields a substantial shift in electrical parameters, which makes it possible to design gas sensors working at RT as a prerequisite to low-powered detection units. Herein, we consider to review such sensors, primarily based on Ti_3_C_2_T_x_ and Ti_2_CT_x_ crystals as the most studied ones to date, delivering a chemiresistive type of signal. We analyze the ways reported in the literature to modify these 2D nanomaterials for (i) detecting various analyte gases, (ii) improving stability and sensitivity, (iii) reducing response/recovery times, and (iv) advancing a sensitivity to atmospheric humidity. The most powerful approach based on designing hetero-layers of MXenes with other crystals is discussed with regard to employing semiconductor metal oxides and chalcogenides, noble metal nanoparticles, carbon materials (graphene and nanotubes), and polymeric components. The current concepts on the detection mechanisms of MXenes and their hetero-composites are considered, and the background reasons for improving gas-sensing functionality in the hetero-composite when compared with pristine MXenes are classified. We formulate state-of-the-art advances and challenges in the field while proposing some possible solutions, in particular via employing a multisensor array paradigm.

## 1. Introduction

Since 2011, there has been a high level of scientific interest in 2D nanomaterials, called MXenes, with the general formula M_n+1_X_n_T_x_, where M is an early transition metal such as Ti, V, Nb, Cr, or Mo; X is C or N; and T is a surface functional group, primarily –F, –OH, or –O, which have unique properties due to a layered structure and variations in elemental composition [1,2,3]. These materials are distinguished by: (i) a high surface-to-volume ratio, which allows for an efficient transfer of surface gas adsorption into electrical properties when combined with an extremely low electrical noise; (ii) a high electrical conductivity that extremely depends on the synthesis method and may vary from metallic to semiconducting nature; and (iii) a high variability of available compositions that could be tuned via the chemical nature of the metal and/or functional groups on the surface in order to adjust the bandgap and work function. These remarkable features of MXenes provide researchers with great opportunities for numerous applications in diversely targeted units. Among others, the high specific surface makes it possible to harden composite materials of different chemical natures [4,5,6,7,8]. The possibility to intercalate various ions or molecules between the 2D layers [9,10,11] and high electrical conductivity led to a focus of major interest on the MXene application in R&D of energy generation and storage devices, lithium/sodium-ion batteries and supercapacitors [12,13,14,15,16,17,18], and fuel cells [19,20]. They are also widely considered in photo- and electrocatalytic processes for CO_2_ reduction, hydrogen production, industrial water purification, electrocatalytic nitrogen reduction to form ammonia, etc. [21,22,23] and to develop conducting coatings in optoelectronics [24,25].

**Figure 1:**
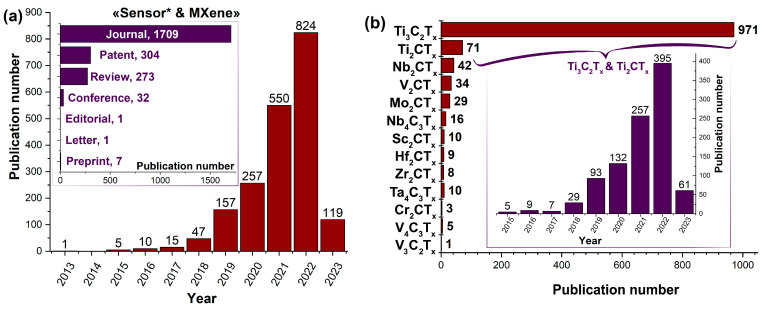
Analysis of reports devoted to MXenes: (**a**) the annual number of publications indexed with keywords “Sensor* and MXene”; insert is distribution of publications by type; (**b**) distribution of articles depending on the kind of MXene; insert is annual number of publications indexed for Ti_3_C_2_T_x_ and Ti_2_CT_x_. The data were taken with CAS, SciFinder^n^ (September 2022).

However, one of the most prospective ways to employ such a material lies in gas sensors. As noted, the targeted surface functionalization of MXenes could advance a selectivity for gas adsorption that could be registered even at room temperature (RT), which is important for energy-efficient sensor devices. Therefore, MXene structures are widely studied as sensing materials [26,27,28,29,30,31,32]. The number of publications in 2021–2022 with keywords “Sensor* and MXene” (CAS, SciFindern, September 2022) cumulatively increases by more than 2.5 times compared with the ones reported in 2013–2020 (Figure 1a), which was 1374 against 492. So far, the major focus has been on investigating the receptor properties, such as carbide MXenes, of Ti_3_C_2_T_x_ and Ti_2_CT_x_ formulas (Figure 1b).

In spite of the abundance of numerous review reports on the application of MXenes for liquid and gas sensors [33,34,35,36,37,38,39,40,41,42,43,44,45,46,47,48,49,50,51], there is some gap in the systematic research on employing titanium-containing phases of Ti_3_C_2_T_x_ and Ti_2_CT_x_ as receptor materials for chemiresistive gas sensors, including (i) a theoretical analysis of sorption and charge transfer, (ii) generalization of experimental data on the influence of the nature of functional surface groups and different modifiers, (iii) systematization of information on the gas sensitivity of nanocoating, and (iv) the analysis of the influence of the nature of functional surface groups and different modifiers. These issues are thoroughly considered in the present review.

## 2. Ti_3_C_2_T_x_ and Ti_2_CT_x_ MXenes Preparation Methods

First report on synthesis of 2D Ti_3_C_2_ carbide as a result of selective removal of Al atom layer from the structure of Ti_3_AlC_2_ MAX-phase [52] was published just 11 years ago by Naguib and coworkers [1]. Already in the primary works by Gogotsi and Barsoum group [1,2,3], the method of aluminum etching by HF acid according to the following reaction was considered:

M_n+1_AlX_n_ + 3HF = M_n+1_X_n_ + AlF_3_ + 1.5H_2_ (1)

M_n+1_X_n_ + 2HF = M_n+1_X_n_F_2_ + H_2_ (2)

M_n+1_X_n_ + 2H_2_O = M_n+1_X_n_(OH)_2_ + H_2_ (3)

This method became possible due to the considerably different nature and strength of the M–X (X = C, N) chemical bonds in M_n+1_X_n_ layers and M–Al chemical bonds, which are responsible for keeping these layers together in MAX-phases. The former bonds are strong covalent–metal interactions, while the latter M–Al bonds are relatively weak, mostly metallic bonds, which can be torn by reacting with certain compounds. In case of MAX-phases including more electronegative element of Si instead of Al as binding element, an oxidizing agent is needed in addition to HF, such as H_2_O_2_, FeCl_3_, HNO_3_, NH_4_S_2_O_8_, KMnO_4_ etc. [53].

The high toxicity of hydrofluoric acid used in the etching process and its excessively high reactivity result in not only more defective MXenes but also a dramatic heating of the reaction system at the initial stage of synthesis. In its turn, together with a large outgassing, it often leads to spewing from the vessel or to oxidation of the resulting MXene particles. Therefore, another milder approach has been developed to selectively etch Al under the influence of HF generated in situ via interacting hydrochloric acid with lithium, sodium, potassium, and iron fluorides [54,55,56,57,58]. With relatively slow release of HF molecules, the synthesis process of MXenes can be easier controlled, and the output layered products appear to be less defective and to contain relatively few M_n+1_X_n_ layers. In addition, the etchant composition significantly affects the kind of surface functional groups that form, such as –F, –OH, and –O, –Cl, which frequently manage the electrochemical properties of MXenes. Application of the NH_4_HF_2_-HCl system also allows one a simultaneous delamination due to intercalation of NH^4+^ cation into the interlayer space [59].

Obtaining single or few-layered MXene plates using the “HCl–MF” etching system, where M is Li^+^, Na^+^, K^+^, NH^4+^ cations, etc., usually requires an additional stage of delamination, i.e., exposure to intense ultrasound or organic molecule intercalation followed by splitting. This often results not only in the M_n+1_X_n_ layers’ separation but also in the reduction of their surface area and an increased defect rate. In order to get large and non-defective Ti_3_C_2_T_x_ particles, the technique of Ti_3_AlC_2_ MAX-phase interaction with HCl-LiF system was optimized to be called Minimally Intensive Layer Delamination (MILD) [60,61,62,63,64]. In this case, the higher *n*(LiF):*n*(M_n+1_AlX_n_) and *n*(HCl):*n*(LiF) ratios facilitate etching of aluminum layers and make it possible to separate accordion-like MXene aggregates into individual plates without using ultrasound; just shaking is sufficient. The morphology of MXenes synthesized by the MILD method, their arrangement in aggregates after drying, as well as their mechanical and electrophysical properties, essentially differ from those of products derived by the conventional etching method with lithium fluoride solution in hydrochloric acid.

Hydrothermal etching technique conducted at 270 °C with assistance of NaOH [65] is one of the most practically interesting for obtaining Ti_3_C_2_T_x_ MXenes with only OH– and O– species on the surface.

Some works [66,67] show the prospects of preparing fluorine-free MXenes as a result of electrochemical etching via taking, for example, HCl acid as a medium under moderate heating [67]:

Ti_3_AlC_2_ − 3ē + 3Cl^−^ = Ti_3_C_2_ + AlCl_3_ (4)

Ti_3_C_2_ + 2OH^−^ − 2ē = Ti_3_C_2_(OH)_2_ (5)

Ti_3_C_2_ + 2H_2_O = Ti_3_C_2_(OH)_2_ + H_2_ (6)

The known problem of rather fast oxidation of MXenes in aqueous media as well as the fact that the nitride MXenes are formed with a very low yield in contrast to the carbide phases when using etching with HF or HCl-LiF solutions led to the development of synthesis methods in molten salts. In Refs. [55,68], a reaction route to fabricate Ti-containing MXenes in the melts of LiF-NaF-KF and NaF-KF fluoride systems was proposed as follows:

Ti_2_AlC + Na^+^ + 2K^+^ + 6F^−^→Ti_2_CT_x_ + K_2_NaAlF_6_ (7)

The functional parameters of MXenes, such as bandgap, electron mobility at RT, work function, etc., depend very strongly on the surface groups. However, for some applications, the fluoride substituents are not suitable. Therefore, the approach of thermal treatment in molten chlorides, such as ZnCl_2_ [69,70] and other Lewis acids, such as CuCl_2_ [71], proved to be quite effective. In the case of ZnCl_2_ melt, either the substitution of Al atoms by Zn in the MAX phase, Reactions (8) and (10), or the synthesis of Cl-substituted Ti_3_C_2_Cl_2_ MXene with release of zinc metal particles as a by-product, Reactions (9) and (11) occur in dependence on the proportions of the components [69,70]:

Ti_3_AlC_2_ + 1.5ZnCl_2_ = Ti_3_ZnC_2_ + 0.5Zn + AlCl_3_↑ (8)

Ti_3_AlC_2_ + 1.5ZnCl_2_ = Ti_3_C_2_ + 1.5Zn + AlCl_3_↑ (9)

Ti_3_C_2_ + Zn = Ti_3_ZnC_2_ (10)

Ti_3_C_2_ + ZnCl_2_ = Ti_3_C_2_Cl_2_ + Zn (11)

The prepared chlorine- and bromine-substituted MXenes are convenient to further modify the surface groups to –S, –Se, and –Te ones in case of necessity to adjust the material properties [72].

In addition, alternative methods for MXene preparation, such as chemical vapor deposition (CVD), other gas-phase methods, and template synthesis, have been actively developed in recent years [73,74,75,76,77].

As a result of liquid-phase etching, packages of MXenes are formed with layers bound to each other by Van der Waals forces. Their morphology is very recognizable and has been almost universally named “accordion-like MXenes.” Depending on the method of synthesis, the degree of aggregation can vary greatly. For example, employing acid solutions of lithium/sodium/ammonium fluorides not only allows one to selectively remove layers of aluminum atoms from MAX-phase structures, but due to cation intercalation, the parallel process of delamination is also possible.

Typical and historically first method of delaminating the MXene layers was a separation under the influence of ultrasound [78], whose prolonged application results in a reduction of the MXene plate area. However, intercalation of some organic molecules greatly facilitates and accelerates the process. Among the most frequently used ones are tetramethylammonium and tetrabutylammonium hydroxides, dimethyl sulfoxide, isopropylamine, urea, and others.

## 3. Application of Individual Ti_3_C_2_T_x_ and Ti_2_CT_x_ MXenes in Chemiresistive Gas Sensors

### 3.1. Sensors Based on Pristine Ti_3_C_2_T_x_

One of the first theoretical works aimed at the analysis of the possibility to employ MXene Ti_3_C_2_T_x_ as a receptor material in chemiresistive gas sensors, particularly for ammonia detection, has been published in [79], which in addition to experimental studies simulated the interaction between MXene with completely substituted oxygen surface groups of the Ti_3_C_2_O_2_ formula and eight gases: CH_4_, H_2_S, H_2_O, C_2_H_5_OH, CH_3_OH, CH_3_C(O)CH_3_, NH_3_, and NO. First-principles calculations made it possible to determine the optimal adsorption geometry and evaluate the energy of this process [79]. The E_ads_ values indicate significantly stronger interactions of NH_3_, ethanol, methanol, and acetone molecules with the Ti_3_C_2_O_2_ surface compared with that of CH_4_, H_2_S, and H_2_O. The expression of E_ads_ in normalized eV/atom units, not in eVs, strongly emphasizes the ammonia molecule, which delivers the characteristic charge transfer value of 0.153e.

In the work by Khakbaz et al. [80], the electrostatic surface potential (ESP), projected density of states (PDOS), and thermal analysis methods were utilized to study the adsorption of NH_3_, NO, NO_2_, N_2_O, CO, CO_2_, CH_4_, and H_2_S molecules on the surface of MXene phases of Ti_3_C_2_T_x_ with various ratios of surface species that are most commonly formed by the often applied techniques for their synthesis: Ti_3_C(OH)_0.44_F_0.88_O_0.66_ (relatively high F-group content), Ti_3_C_2_(OH)_0.66_F_0.22_O_1.11_ (relatively low F-group content), and Ti_3_C_2_(OH)_0.44_F_0.88_O_0.66_(H_2_O)_0.44_ (with adsorbed water molecules). The calculation results have shown that the composition with high fluoride surface groups of Ti_3_C_2_(OH)_0.44_F_0.88_O_0.66_ is most sensitive to ammonia due to the maximum charge transfer (−0.098e) and minimum adsorption energy (−0.36 eV). It was found that sorption of the NO_2_ molecule on the surface of this composition results in the formation of nitrous acid from HNO_2_ as a result of proton detachment from the OH-group by the adsorbed molecule of the gas, the chemical bonding in the OH-group being weakened due to the high content of electronegative F-groups. It was shown that for MXene substrates, the adsorption energy goes down from −0.36 eV to −0.49 eV with a reduction in the number of F-groups and a corresponding enhancement in the number of oxygen-containing surface substituents, which leads to an increase in the sensor recovery time. At the same time, the charge transfer value for MXenes with different –F/(–OH and –O) ratios does not substantially change. If water molecules are present in the MXene receptor layer, a stabilization of the sorbed NH_3_ molecule on the surface occurs due to the formation of hydrogen bondings. However, in this case, a decrease in the interaction between the titanium and nitrogen atoms and, accordingly, the charge transfer down to 0.012e, i.e., a reduction by almost 90%, were also recorded. Thus, it is shown [80] that the chemical composition of the surface functional groups of MXene Ti_3_C_2_T_x_ as well as the presence of surface-adsorbed water molecules have a significant influence on the chemosensor properties of this material.

**Figure 2:**
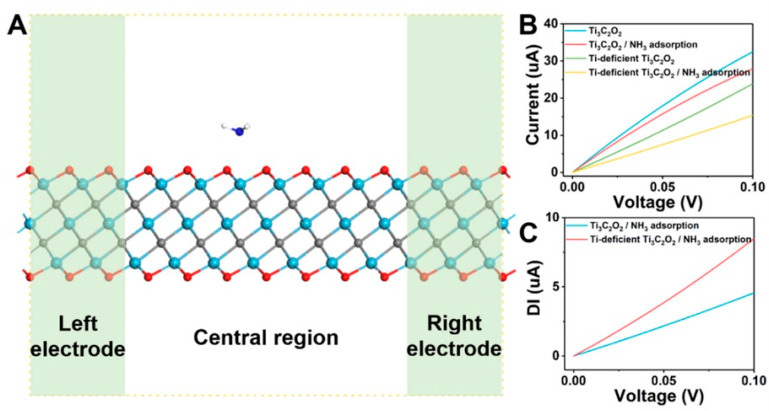
(**A**) Atomic-scale device simulation; (**B**) electronic transport behavior (I–V) of Ti_3_C_2_O_2_ MXene and Ti-deficient Ti_3_C_2_O_2_ MXene after NH_3_ adsorption under series bias voltages; and (**C**) change in current after NH_3_ adsorption. The left and right electrode regions of the two-probe system are in contact with the central region (4 × 4 supercell) to detect NH_3_ molecules. Reprinted with permission from [81]. Copyright 2022: American Chemical Society.

In work [81], density functional theory (DFT) was applied to examine features of the adsorption of ammonia molecules on Ti_3_C_2_O_2_ MXene, which is characterized by titanium defects. It is shown that the stronger physical interaction of the NH_3_ molecule with the MXene monolayer is observed at the titanium deficit, with a higher negative corresponding adsorption energy of −7.44 eV when compared with −6.29 eV for the defect-free Ti_3_C_2_O_2_. At the same time, the charge transfer has slightly decreased, from 0.226e to 0.115e. The calculations using an atomic-scale gas sensor model for studying electron transport indicate (Figure 2) that, in accordance with the I-V curves under ammonia molecule adsorption, there is a more significant current drop for titanium-deficient Ti_3_C_2_O_2_ in contrast to the reference MXene. According to the authors of [81], this proves that the introduction of titanium vacancies can increase the sensitivity of Ti_3_C_2_O_2_-based sensors for NH_3_ detection.

In theoretical work [82] for MXenes with different surface substituents (Ti_3_C_2_O_2_, Ti_3_C_2_F_2_, and Ti_3_C_2_(OH)_2_) and defects (point vacancies), the adsorption process of SF_6_ decomposition products (SO_2_, SOF_2_, and H_2_S) as markers for inevitable partial discharge in gas-insulated switchgear was investigated by DFT. It was shown that no stable adsorption configuration and low adsorption energy were found for the interaction of the defect-free Ti_3_C_2_(OH)_2_ with SOF_2_ and H_2_S gases, whereas for pure Ti_3_C_2_O_2_ and Ti_3_C_2_F_2_, all the optimized adsorption configurations are stable, but the low energy and large adsorption distance imply a very weak adsorption of all the above-mentioned SF_6_ decomposition products. Due to this, calculations with embedded point vacancies for oxygen and for fluorine were performed for the latter, which indicate a 2–4-fold increase in the adsorption energy for all selected test gases. In this case, physical adsorption is replaced by chemisorption with significant charge transfer. The most negative values of adsorption energy on Ti_3_C_2_O_2_ and Ti_3_C_2_F_2_ with the corresponding point vacancies were obtained for SO_2_, which indicates that the highest sensitivity should be observed for its detection.

Another group of researchers, Zeng and colleagues [83], also studied the features of the adsorption of gaseous products of sulfur hexafluoride oxidation (H_2_S, SO_2_, SOF_2_, SO_2_F_2_) on Ti_3_C_2_T_x_ MXene with different functional groups (O, F, OH). Here, DFT calculations allowed the authors to establish that all selected gases should adsorb spontaneously on Ti_3_C_2_O_2_ and Ti_3_C_2_(OH)_2_, and charge transfer reduces in the series Ti_3_C_2_(OH)_2_ > Ti_3_C_2_O_2_ > Ti_3_C_2_F_2_. For the most promising composition, Ti_3_C_2_(OH)_2_, the charge transfer upon adsorption of these four marker gases is in the range from 0.19e (H_2_S) to 1.27e (SO_2_F_2_). The work published in [84] continues a series of theoretical studies by Zeng’s group aimed at studying the efficiency of detection of SF_6_ decomposition products formed as a result of insulation overheating or partial discharge. However, in this case, it is taken into account that sulfur hexafluoride is often used in a mixture with nitrogen to reduce the negative effects of SF_6_ release into the environment. As a result, in addition to sulfur-containing oxidation products, it is necessary to consider the possibility of formation of such molecules as NO, NO_2_, N_2_O and NF_3_. The data of the first-principle calculations exhibited that all compositions of Ti_3_C_2_T_x_ (T = F, O, OH) are predicted to have good prospects for the detection of NO and NO_2_, while Ti_3_C_2_(OH)_2_ has a large negative adsorption energy for all four gases (from −1.7 eV to −9.1 eV, characterizing NO and NF_3_, respectively) and significant charge transfer (−1.6e for NO to −1.4e for NF_3_), which should facilitate the detection, but long recovery times are assumed for these parameters.

As emphasized in one of the first sensing studies by Lee and colleagues [85] involving Ti_3_C_2_T_x_ MXene, continuous monitoring of human physiological state by exhalation composition requires the integration of gas sensors into portable and wearable electronic hardware that is operable at RT and possesses flexible substrates that provide a natural fit to the human body. To study the possibility of using Ti_3_C_2_T_x_ MXene as a gas-sensitive material, derived by selective etching of aluminum from the corresponding Ti_3_AlC_2_ MAX-phase by LiF + HCl (9M) with subsequent water washing and ultrasonic treatment, the dispersion was applied to a flexible polyimide substrate equipped with platinum counter-pin electrodes. The coating was dried in a desiccator. The thickness of the formed multilayer film was 30 nm. An examination of the response to 100 ppm of ethanol, methanol, acetone, and ammonia at RT showed (Table 1) that in all cases there was a response characterized by an increase in resistance and a systematic enhancement of baseline resistance, especially when detecting ammonia. The authors believe [85] that the binding of gas molecules to MXene in the present study is due to interactions with both Ti_3_C_2_T_x_ surface defects and surface functional groups, primarily –O and –OH. According to the authors, the adsorbed water and oxygen molecules introduced under etching the MAX-phase play an important role. Possible reactions occurring during the sorption of the NH_3_ molecule on the MXene surface with a charge transfer have been suggested, with the formation of an electron that then recombines with the hole present in the MXene, resulting in the resistance increase [85]:

2NH_3_ + 3O^−^ → N_2_ + 3H_2_O + 3ē (12)

NH_3_ + OH^−^ → NH_2_ + H_2_O + ē (13)

The responses were 0.210, 0.143, 0.115, and 0.075 for ammonia, methanol, ethanol, and acetone, respectively [85]. The data obtained are consistent with the available theoretical studies [86] and indicate an increased absorption energy for the Ti_2_CO_2_-NH_3_ pair, which may account for the baseline drift and the high sensor recovery times observed in study [85]. In addition, the change of response to acetone in the concentration range of 25–200 ppm was studied in [85] to observe a linear dependence.

The scientific task set forth in [87] relates to the need for a correct determination of low concentrations of volatile organic compounds (VOCs) in the ppb range at RT, where a maximum signal-to-noise ratio should be observed. It was found that the deposited receptor layer of Ti_3_C_2_T_x_ MXene synthesized under etching by LiF + HCl according to a methodology similar to one reported in [85] showed a high response to 100 ppm ethanol, acetone, propanal, and ammonia. For ethanol, the response was almost twice as high as that for ammonia. DFT simulations highlighted this feature by the predominant presence of F and OH as surface functional groups, and the highest estimated sensitivity to both gases was predicted for the hydroxide-substituted Ti_3_C_2_(OH)_2_ MXene. For acetone, ethanol, and ammonia, i.e., gases capable of participating in hydrogen bonds, the detection limit was defined as equal to 50–100 ppb [87]. The authors noted the record-low electrical noise of the sensor, especially under the detection of VOCs, which is due to the high electrical conductivity of 3250 S/m of the MXene.

Wu and colleagues [79] experimentally investigated the gas-sensitive properties of single-layer Ti_3_C_2_ MXene, synthesized by etching the corresponding MAX phase with sodium fluoride solution in hydrochloric acid, at 25 °C as part of a chemiresistive sensor in a cylindrical coating geometry versus to 500 ppm of a number of gases (CH_4_, H_2_S, H_2_O, NH_3_, NO, ethanol, methanol, and acetone) under a varying humidity background. A high selectivity towards ammonia was found; the second most intense response to ethanol is only 24% of that to NH_3_. It seems to relate to the high adsorption energy of this analyte. The authors noted a significant increase in the response to ammonia when compared with the similar Ti_3_C_2_ sample obtained via etching with LiF + HCl [85]. This is explained by the cleaner impurities of metal cations on the surface of the MXene layer and, thus, an increased area for adsorption. In experiments performed to study the effect of humidity on ammonia detection [79], the response was found to go up with increasing humidity from 30 rel.% to 40–90 rel.%, and the response values at RH = 50–90% were very close. In addition, the phenomenon of some signal saturation at high NH_3_ concentrations was observed, which seems to be associated with the difficulty of desorption of this gas at RT and leads to a gradual increase in the baseline resistance. As mentioned above, the simulation of Ti_3_C_2_O_2_ interactions with eight gases (CH_4_, H_2_S, H_2_O, C_2_H_5_OH, CH_3_OH, CH_3_C(O)CH_3_, NH_3_, and NO) based on first-principle calculations [79] has shown promising sorption of specifically ammonium molecules, which is characterized by a high adsorption energy and charge transfer. The significant discrepancy between the low experimental sensitivity to NO and the very high values of charge transfer and adsorption energy calculated for this molecule was attributed by the authors [79] to the low content of OH- and F-groups on the surface of MXene.

A significant influence of the composition of the surface groups and the ion intercalation into the interlayer space on the chemosensory behavior of MXenes has been concerned in a fairly wide range of studies [88,89,90]. For example, Yang and colleagues [88] studied the effect of additional treating the Ti_3_C_2_T_x_ MXenes with sodium hydroxide solution on RT sensitivity to ammonia and atmospheric moisture. These MXene structures were synthesized from Ti_3_AlC_2_ MAX-phase with etching in a 45% HF solution at RT. The authors found that such a MXene processing results in (1) the removal of some of the surface F-groups and their replacement by OH-species, increasing the ratio of n(O)/n(F) by 2.5–3 times, and (2) the intercalation of Na^+^ cations into the interlayer space of the material. For the NaOH-impregnated Ti_3_C_2_T_x_ MXene, an inversion of the response to moisture and ammonia influx was observed: there was an increase in conductivity rather than an increase in resistance, as opposed to the original Ti_3_C_2_T_x_. Other sensory characteristics also changed. The experiments at 38 rel.% humidity showed that there was an almost two-fold increase in the response to ammonia and improved selectivity towards, in particular, NO_2_. The authors explained [88] the increased sensitivity to humidity by the formation of aqua-complexes, of [Na(H_2_O)_m_]^+^, on the surface of MXene layers, which advances the adsorption capacity of the material. Increasing the number of surface OH-groups led to improved NH_3_ binding. The simulation data presented in [87] show that the most negative adsorption energy is observed for Ti_3_C_2_(OH)_2_ in comparison to Ti_3_C_2_F_2_ and Ti_3_C_2_O_2_. The change in the signal direction, however, as believed by the authors [88], may be due to a change in the carrier type in Ti_3_C_2_T_x_ after functionalization with oxygen.

Another study [89] also addressed the role of the process of gas intercalation into the interlayer space of the Ti_3_C_2_T_x_ MXene upon detecting gases of different chemical natures as well as the influence of the intercalation of sodium cations as a result of processing in alkaline solutions. It was found that ethanol vapors, to which Ti_3_C_2_T_x_ is sensitive, resulted in a significant shift of the (002) reflex position, which corresponds to an increase in the interlayer distance of the MXene film from 13.24 Å to 14.06 Å. At the same time, such a process does not occur upon CO_2_ exposure, for which Ti_3_C_2_T_x_ lacks gas sensitivity. Preliminary intercalation of sodium cations as a result of impregnation of MXene layers with NaOH solutions of various concentrations leads to an increased response to both ethanol and CO_2_, since the adsorption of ethanol in the interlayer space is controlled by hydrogen bonds with functional surface groups of O/OH and the water present. It was found that there is an optimal concentration of NaOH solution of 0.3×10^−3^ mol/L, which allows one to obtain the maximum selectivity when detecting C_2_H_5_OH vapors. As a result, the authors in [89] conclude that the cause of the universal positive response to gaseous analytes of different natures (oxidants and reducing agents), along with the influence of the metallic nature of MXene conductivity, arises from interlayer swelling due to difficulties in electron transfer outside the Ti_3_C_2_T_x_ layer plane, where an increase in electrical resistance occurs.

The effect of ionic intercalation upon addition of electrolyte solutions (HCl acid, neutral KCl salt, and KOH alkali) to the colloidal solution of delaminated Ti_3_C_2_T_x_ MXenes, synthesized upon exposing Ti_3_AlC_2_ powder to a mixture of lithium fluoride with hydrochloric acid, on the sensitivity of chemiresistive gas sensors has been considered in research reported in [90]. As a part of the study, the optimal concentrations of modifying solutions, which do not lead to MXene aggregates, were established as being equal to 0.01 M for KCl and KOH and 0.001 M for HCl. It was shown that the introduction of KOH, in contrast to KCl and HCl, makes it possible to increase the number of surface OH-groups and also leads to the formation of films with the largest interlayer distance in MXenes due to the intercalation of potassium cations. The authors attribute the efficiency of potassium ion intercalation into the interlayer space of MXene specifically for the Ti_3_C_2_T_x_-KOH sample to the increased content of surface OH-groups capable of deprotonation, the formation of the enolate form (Ti-O-), and further intermediate compounds (Ti-O-K). The obtained Ti_3_C_2_T_x_ -KOH structure exhibited the highest response when detecting 100 ppm ammonia [90]. The response was 3.8%, which is higher than the 0.9% observed in the pristine Ti_3_C_2_T_x_. The minimum response was observed for the KCl-treated sample, equal to 0.4%. Sensitivity analysis to ethanol vapor, 100 ppm, showed that the Ti_3_C_2_T_x_-KOH sample had the highest response to this analyte, ~3.4%, which was significantly higher than that of the Ti_3_C_2_T_x_ (0.8%) and the other samples (<0.5%). The study of the response of the obtained sensors under a higher level of background humidity (RH = 30%) revealed that the response to ethanol for the Ti_3_C_2_T_x_-KOH, though significantly decreased, was still approx. four times higher than the response of other samples. With further increasing the humidity up to 50 rel.%, responses significantly deteriorate and become close for all the samples, equal to 0.25–0.5%, which the authors attribute to the additional adsorption of water molecules on the surface of MXene, which blocks adsorption centers for the analyte gases.

**Figure 3:**
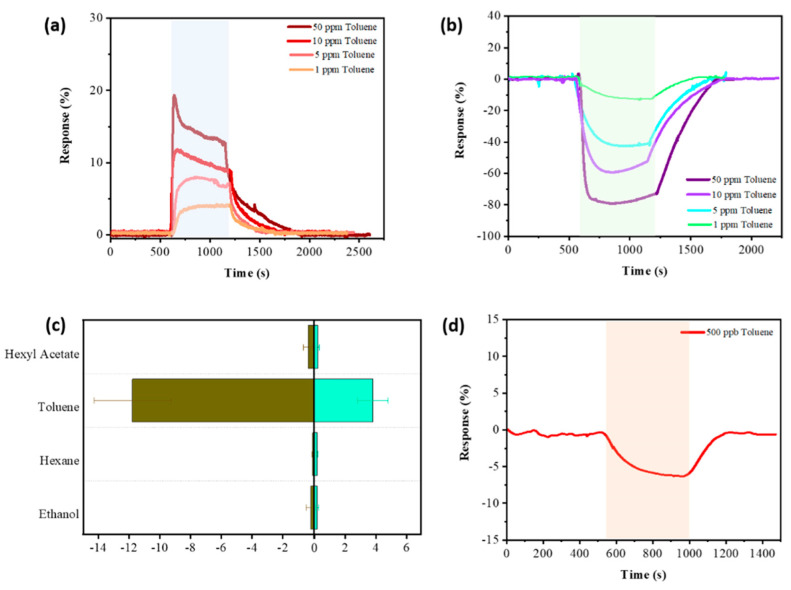
(**a**) Dynamic response curve of the pristine/undoped Ti_3_C_2_T_x_ MXene sensor for toluene response at 1, 5, 10, and 50 ppm at RT, (**b**) dynamic response curve of sulfur-doped Ti_3_C_2_T_x_ MXene sensors for toluene response at 1, 5, 10, and 50 ppm at RT, (**c**) comparison of the real-time response between undoped and doped Ti_3_C_2_T_x_ MXene sensors upon exposure to hexyl acetate, toluene, hexane, and ethanol at a concentration of 1 ppm, (**d**) dynamic response curve of the sulfur-doped Ti_3_C_2_T_x_ MXene sensor at 500 ppb toluene exposure. Reprinted with permission from [91]. Copyright 2020: American Chemical Society.

In a study by Shuvo et al. [91], taking into account such drawbacks of MXenes as their tendency to agglomerate under coating formation and a rather small interlayer distance, which hinders gas diffusion and reduces the effective surface-to-volume ratio, the effect of Ti_3_C_2_T_x_ doping with sulfur heteroatoms on the chemisensor properties was studied. For this purpose, the fresh Ti_3_C_2_T_x_ nanomaterial synthesized by the exposure of Ti_3_AlC_2_ powder to LiF + HCl was ground in a mortar with thiourea and heat-treated at 500 °C for 3 h in an Ar atmosphere. It was found that the alloying allowed for increasing the interlayer distance, which was expressed in a shift of the (002) reflex relative to that for Ti_3_C_2_T_x_ as well as confirmed by HR-TEM inspection; a more than two-fold increase in the distance between the layers was observed, from 0.96 nm (Ti_3_C_2_T_x_) to 1.91 nm (Ti_3_C_2_T_x_-S). For the Ti_3_C_2_T_x_ and Ti_3_C_2_T_x_-S receptor materials deposited on a flexible polyethylene terephthalate substrate equipped with 20 Ag counter-pin electrodes, responses to a number of VOCs were measured. An unexpected fact was established: among the studied series of analytes (hexyl acetate, toluene, hexane, and ethanol), the highest response for both sensors was recorded for toluene vapor, as shown in Figure 3. For the Ti_3_C_2_T_x_ sample, the conventional increase in resistance was observed upon a gas injection, while for the Ti_3_C_2_T_x_-S sample, there was a decrease in resistance. This anomalous behavior of the sensor based on sulfur-doped Ti_3_C_2_T_x_ was attributed by the authors to the presence of sulfur atoms on the surface, which leads to the extraction of electrons from the conduction band of MXenes with the formation of ionic forms of S^γ−^, e.g., S^−^, S^2−^, and an electron-depleted layer. These processes lead to increased receptor layer resistance. Following the suggestion of the authors, when a gaseous analyte appears, chemical processes with a reduction in sulfur compound concentration are possible, such as forming oxygen ionic species in MOS sensors, which can thereby increase the electron concentration and, consequently, advance the conductivity of the material.

The response value for sulfur-doped MXene exceeded the same for pristine MXene by 3–4 times. For example, the response to 50 ppm toluene for Ti_3_C_2_T_x_-S was ca. −79.5%, while for Ti_3_C_2_T_x_ it was about +19.3%, which the authors related to increasing the interlayer distance in the MXene [91]. For the Ti_3_C_2_T_x_-S sample, a detection limit of 500 ppb toluene was adjusted. Still, the higher sensitivity to toluene for both sensors is explained by the authors to come from the higher activity of the aromatic ring, which is enhanced by the methyl group due to its electron-donating effect. DFT calculations showed that the bond energy of the toluene molecule to the sulfur-doped functional surface groups has a much more negative value than for Ti_3_C_2_O_2_, which is consistent with the experimentally observed increased response for the Ti_3_C_2_T_x_-S sample.

**Figure 4:**
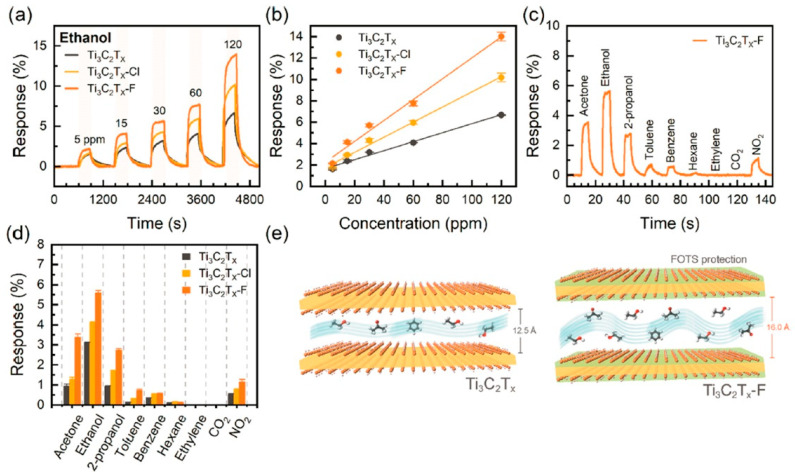
(**a**) Dynamic response curves of Ti_3_C_2_T_x_, Ti_3_C_2_T_x_-Cl, and Ti_3_C_2_T_x_-F sensors upon exposure to varying concentrations of ethanol from 5 to 120 ppm, and (**b**) the response versus ethanol concentration plots for the three sensors derived. (**c**) Sensing response of Ti_3_C_2_T_x_-F sensors toward 30 ppm of acetone, ethanol, 2-propanol, toluene, benzene, hexane, ethylene, carbon dioxide, and nitrogen dioxide at RT. (**d**) Maximal response changes upon exposure to various gases. (**e**) Schematics of Ti_3_C_2_T_x_ and Ti_3_C_2_T_x_-F upon exposure to VOCs. Reprinted with permission from [92]. Copyright 2020: American Chemical Society.

The problem of the rather low oxidative stability of MXenes is discussed in the study by Chen et al. [92], who proposed an alternative way to stabilize their structure via a surface functionalization with perfluoroalkoxysilane for further application in the detection of VOCs. For this purpose, the Ti_3_C_2_T_x_ MXene powder obtained by etching the MAX phase of Ti_3_AlC_2_ with LiF/HCl was functionalized in (3-chloropropyl)trimethoxysilane and 1H,1H,2H,2H-perfluorooctyltriethoxysilane solutions, which interacted with the existing O- and OH-groups on the surface to yield Ti_3_C_2_T_x_-Cl and Ti_3_C_2_T_x_-F samples, respectively. In this case, superhydrophobicity was demonstrated for the latter, expressed in a sharp increase of the boundary wetting angle from 33 to 156. It was also noted that the microstructure of Ti_3_C_2_T_x_-F is not characterized by the accordion-like aggregates, typical for the original Ti_3_C_2_T_x_ and Ti_3_C_2_T_x_-Cl, but instead appears in a porous three-dimensional architecture consisting of crumpled nanosheets, which facilitates gas diffusion. It was noted that storing the samples for 2 weeks under 100 rel.% humidity led to only a slight reduction of the electrical conductivity of the modified Ti_3_C_2_T_x_-F sample of ca. 14%, which was further restored to 93% of the original value under a dry environment. In contrast, the same humidity storage resulted in a catastrophic 97% decrease in the electrical conductivity of the Ti_3_C_2_T_x_ sample. For both modified receptor materials, an enhanced response to all the studied VOCs was recorded, primarily to ethanol, acetone, and 2-propanol, which the authors attributed to a significant enlarging of the interlayer distance (Figure 4) [92].

Thus, the responses to 120 ppm ethanol for the Ti_3_C_2_T_x_-F sample exceeded those for the original Ti_3_C_2_T_x_ MXene by more than two times and those for the Ti_3_C_2_T_x_-Cl sample by approximately 1.5 times. For the Ti_3_C_2_T_x_-F sample, it was found that the response to 30 ppm ethanol systematically decreased from 5.7% to 1.5% when the relative humidity of the gas environment went up from 5% to 80%. This seems to be due to a partial filling of the receptor material’s active centers with water molecules. Altogether, it was found that modification of the surface of Ti_3_C_2_T_x_ nanosheets with a perfluoroalkyl substituent leads to a significant increase in coating stability under the influence of oxygen and moisture for a long time, as well as to a significant improvement in gas-sensitive properties to VOCs at room temperature.

**Figure 5:**
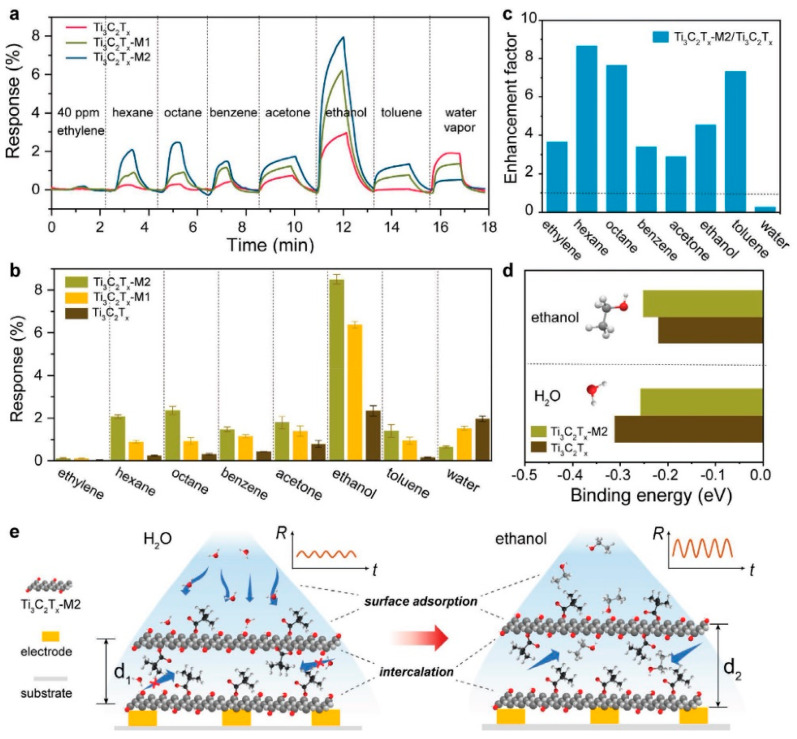
Enhanced VOCs sensing performances and sensing mechanisms of termination-modified Ti_3_C_2_T_x_. (**a**) The 40 ppm gas response curves and (**b**) equilibrium responses with error bars (N = 3) of pristine Ti_3_C_2_T_x_, Ti_3_C_2_T_x_-M1, and Ti_3_C_2_T_x_-M2 sensors, respectively. Notably, the concentrations marked in figures were approximate values. (**c**) The enhancement factor of Ti_3_C_2_T_x_-M2 sensor against VOCs and water vapor sensing, calculated by gas responses of Ti_3_C_2_T_x_-M2 divided by those of pristine Ti_3_C_2_T_x_. (**d**) DFT simulations for ethanol and H_2_O molecules absorbed on pristine Ti_3_C_2_T_x_ and Ti_3_C_2_T_x_-M2. (**e**) Schematic illustration of the H_2_O and ethanol sensing mechanism of Ti_3_C_2_T_x_-M2. R, the resistance of the sensor; t, time. Reprinted with permission from [93]. Copyright 2021: Wiley-VCH GmbH.

The study by Li et al. [93] deals with developing a Ti_3_C_2_T_x_-based sensor for VOCs in exhaled air, aimed at noninvasive detection of diseases. As numerous works highlight [31], the MXene structures are extremely sensitive to humidity, which could interfere with VOCs and eliminate the response to other gases. The peculiarity of the exhaled air, where the water vapors exceed by hundreds of times the concentration of the target analytes, can lead to the negation of the available signals of the latter. In view of this problem, the authors tried to increase the hydrophobicity of Ti_3_C_2_T_x_ receptor coatings synthesized from MAX phase exposure to LiF/HCl by grafting a branched hydrocarbon substituent onto the surface OH-groups as a result of interaction with trimethylacetic anhydride to obtain the sample called Ti_3_C_2_T_x_-M1. The second sample of Ti_3_C_2_T_x_-M2 was additionally exposed to NaHCO_3_ solution to increase the surface hydroxo-groups required for grafting the (CH_3_)_3_COO substituent. It was found that there is a significant shift in the XRD reflex position of the (002) plane in Ti_3_C_2_T_x_-M1 when compared with Ti_3_C_2_T_x_, going from 8.8° to 6.8°, which indicates an increase in the interlayer distance due to the introduction of bulk surface substituents. For the sample of Ti_3_C_2_T_x_-M2, however, in addition to an even greater shift of the same reflex position down to 6.3°, a significant change in morphology with the formation of curved porous flake aggregates was observed. The authors explained it by a random distribution of (CH_3_)_3_COO-groups on the surface, which weakens a polarity for local areas and leads to non-uniform interaction between Ti_3_C_2_T_x_ layers. Due to an almost four-fold increase in the specific surface area, from 10 m^2^/g characterizing pristine Ti_3_C_2_T_x_ to 39 m^2^/g for Ti_3_C_2_T_x_-M2, and the facilitation of gas diffusion into the interlayer space, a sharp increase in responses to all gases under test, including non-polar gases such as hexane, octane, and aromatic hydrocarbons, has been observed, as pictured in Figure 5. 

However, the largest response was obtained for 40 ppm ethanol [93]. At the same time, by introducing a bulk organic surface modifier, the response to water vapor decreased most significantly, from 2% for the original Ti_3_C_2_T_x_ to 0.6% for Ti_3_C_2_T_x_-M2. When detecting ethanol in the range of 20–500 ppm, it was found that there was an increase in the response in a power-dependent manner for all three samples, with the modified Ti_3_C_2_T_x_-M2 sensor having the highest response [93]. Experiments on the long-term stability of the sensor performance showed that the Ti_3_C_2_T_x_-M2 sample is characterized by a mild increase in the baseline associated with a decrease in conductivity due to some oxidation of the carbide core. At the same time, the responses to hexane and ethanol were reduced after 15 days of ambient exposure by only 41.9% and 31.7%, while the unmodified Ti_3_C_2_T_x_ lost ca. 90% of its sensitivity during this time. It is worth noting that the most promising sample, Ti_3_C_2_T_x_-M2, was integrated into a wearable tag for VOC detection as a part of a face mask and demonstrated the ability to distinguish between normal breath and breath after alcohol consumption.

In recent years, there has been increasing interest in the use of non-delaminated MXenes, which are characterized by an accordion-like microstructure to be preserved during coating with a large specific surface area [94,95]. The structures of Ti_3_C_2_T_x_ were successfully employed to produce an ultrafast humidity sensor [94]. To enrich the MXene surface with OH-substituents under an accordion-like architecture, after selective etching of Al from Ti_3_AlC_2_ by a concentrated HF acid, washing with distilled water, and drying in a vacuum, the layered aggregates of Ti_3_C_2_T_x_ were not subjected to delamination. It was found that the chemiresistive response is reversible and follows a linear function in the relative humidity range of 11–90%. The sensor response and recovery times were estimated to be ca. 60 s. FT-IR studies for Ti_3_C_2_T_x_ in dry air and at 95% relative humidity indicate the appearance of a layer of liquid water on the surface of the MXene in the latter case. The authors explain this by the abundance of OH-groups on the surface, which can form continuous and ordered films of H_2_O in the hierarchical Ti_3_C_2_T_x_ nanostructures, which are quickly grown by exposure to water vapor. In this case, the water molecules are stacked on top of each other, making an electrostatic field directed perpendicular to the surface of the MXene, which prevents the carrier transfer and, consequently, leads to an increase in the resistance of the receptor material.

The further evaluation of the efficiency of employing the accordion-like Ti_3_C_2_T_x_ derived by selective etching of Al layers from the MAX phase of Ti_3_AlC_2_ with concentrated HF (without delamination) as a sensor to detect low concentrations of acetone at 23 °C was performed by Majhi et al. [95]. They found out that the screen-printed receptor coatings could allow one to measure acetone at concentrations down to 0.25 ppm when the observed response was 17.3%. The response to acetone was quite selective. At a concentration of 100 ppm, it was equal to 99%, while one to 100 ppm of H_2_ was only 15%. Notably, the sensor performance was preserved versus at least acetone vapors while storing the unit for 3. Under RH = 50%, the acetone response reduced from 99% (at RH = 0%) to 80%, though the humidity at the 90 rel.% pressure forced the response to drop to ca. 5%.

Another study published in [96] is somewhat different from the already described studies. However, it is worth noting as an important example of a virtual sensor design capable of fairly accurate detection of ethanol content in the presence of water and methanol. The idea was to employ a broadband impedance spectrum from a single Ti_3_C_2_T_x_-based sensor and to process it by principal component and linear discriminant analysis methods. For this purpose, the sensor was exposed to 100–800 ppm of different VOCs (methanol, ethanol, acetone, isopropanol, acetonitrile, dichloromethane, hexane, and toluene) to record impedance over a wide range of AC frequencies. The authors have extracted eight representative parameters for each analyte at each concentration. Thus, unique fingerprints were created to characterize the gaseous test analytes, which were further used for their identification, including their appearance in complex gas mixtures. By performing multivariate mathematical data processing as well as cross-checking, the authors ensured a high level of gas identification and prediction of its concentration for the detection of tested VOCs in the presence of known and unknown interferences.

### 3.2. Sensors Based on Pristine Ti_2_CT_x_

The number of works, both theoretical and experimental ones, dealing with the application of Ti_2_CT_x_ MXene as a receptor material for chemiresistive gas sensors is significantly less when compared with Ti_3_C_2_T_x_. Nevertheless, the adsorption of gases on the Ti_2_CT_x_ surface has been considered theoretically already in 2015. In this study, Yu et al. [86] performed first-principles simulations to simulate the interaction between Ti_2_C MXene with oxygen surface functional groups and ammonia molecules. It was found to have a rather high charge transfer value, 0.174e from the nitrogen atom to the MXene, and a negative adsorption energy of −0.37 eV, which should result in the chemisorption of a given molecule on the Ti_2_CO_2_ surface (which is also expressed in distortion of both NH_3_ and solid phase geometry in the Ti_2_CO_2_ monolayer approach) and, alternatively, should allow for a relatively fast desorption at RT. At the same time, it is shown that application of 3% strain to the layer leads to an even greater increase in the adsorption energy of NH_3_ on the Ti_2_CO_2_ surface, up to 0.51 eV.

Thomas and coworkers used [97] the DFT method for estimation of phosgene adsorption onto the Ti_2_CT_x_ MXene with surface substituents by F-, OH-, and O- to evaluate the effect of both the nature of functional groups and atomic defects. Analysis of the adsorption energy of the COCl_2_ molecule over Ti_2_C, Ti_2_CF_2_, Ti_2_CO_2_, and Ti_2_C(OH)_2_ monolayers indicated that Ti_2_C(OH)_2_, including the defective one, is the most promising structure to efficiently detect this gas. Moreover, the maximum charge transfer, of 0.310–0.311e, is characteristic for pure Ti_2_C(OH)_2_ and carbon-deficient materials. Another theoretical study [98] shows that a sufficiently high amount of water molecules adsorbed on the surface of the Ti_2_C MXene facilitates a sharp, almost by an order of magnitude, increase in the response when detecting ethanol when compared with that for pure Ti_2_C.

For the thinnest carbide MXene of Ti_2_CT_x_, both chemistry and experiments concerning gas-sensing properties have been studied in much less detail compared with Ti_3_C_2_T_x_, despite the fact that, due to the smaller single layer thickness, it seems more promising for use in areas where surface processes are critical. The influence of the synthesis method and hence the composition of the surface functional groups on the sensory properties of the Ti_2_CT_x_ has been studied by Sun et al. [99]. It was found that in the case of selective removal of Al from the Ti_2_AlC MAX-phase using concentrated HF, which leads to an increased content of F-groups and a reduced content of OH-groups, 2D nanomaterials with *p*-type semiconductor properties are formed. At the same time, the technique of exposure to a mixture of hydrochloric acid and lithium fluoride allows one to obtain samples with metallic conductivity and a relatively high content of surface OH-groups. The ammonia sensitivity of this MXene synthesized by hydrofluoric acid etching is approximately 35% higher when compared with one of the Ti_2_CT_x_ MXenes obtained by etching with LiF/HCl.

It was also noted that the Ti_2_CT_x_ (HF) MXene is also characterized by faster desorption and restoration of the baseline; purging the cell with N_2_ was sufficient for this purpose, while the second MXene required additional UV irradiation [99]. This seems to be due to the more negative adsorption energy calculated in [86] for the Ti_2_C(OH)_2_ compound in comparison with Ti_2_CO_2_. Thus, etching of Ti_2_AlC with hydrofluoric acid allows one getting more promising results from a chemical gas-sensing standpoint of Ti_2_CT_x_ MXenes, both in terms of the response value and kinetic parameters, when compared with using a mixture of lithium fluoride and hydrochloric acid.

**Figure 6:**
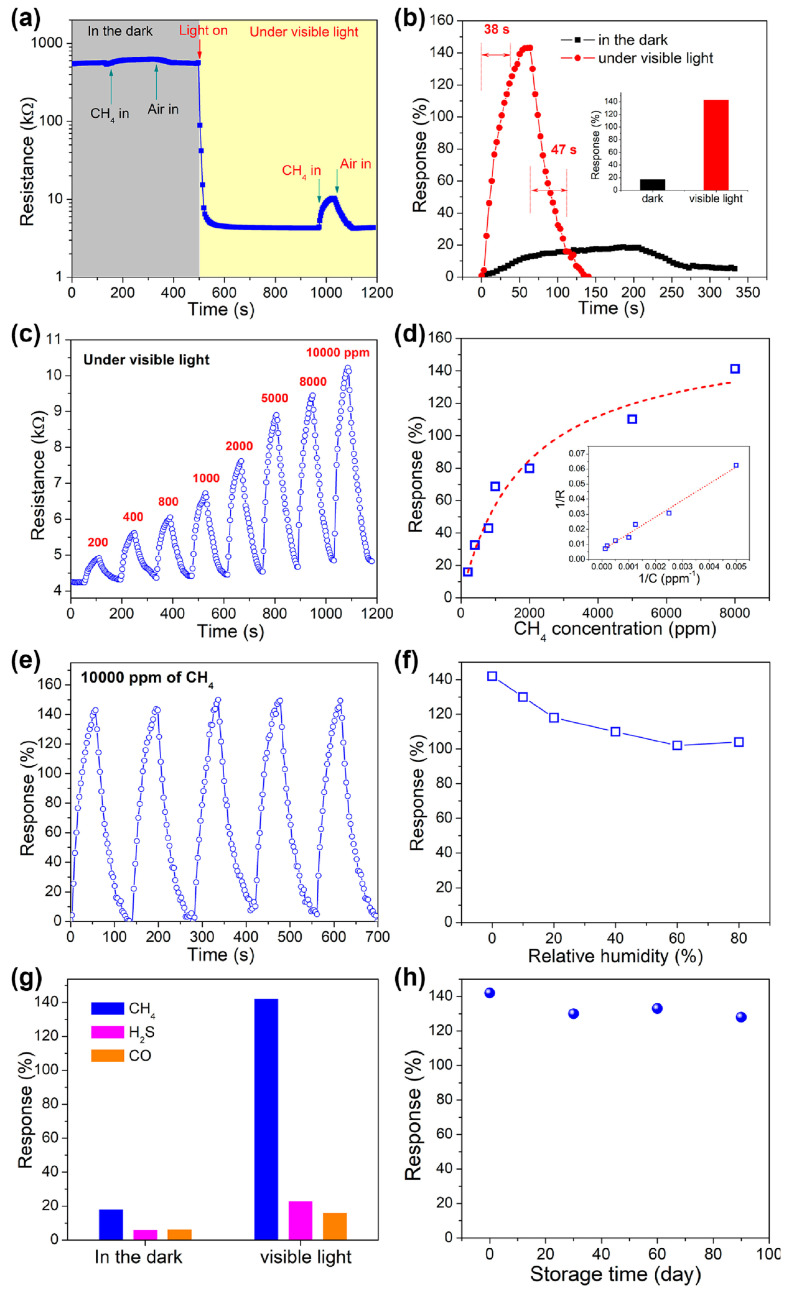
(**a**) Kinetics of resistance change of the Ti_2_CT_x_ sensor with 10,000 ppm of CH_4_ measured continuously with visible-light irradiation on/off; (**b**) dynamic response curves and response values of the Ti_2_CT_x_ sensor to 10,000 ppm of CH_4_ with and without visible-light irradiation; (**c**) dynamic response curve and (**d**) responses of the Ti_2_CT_x_ sensor when 200–10,000 ppm of CH_4_ was applied under visible-light irradiation; inset in (**d**)—linear fitting of 1/R versus 1/C; (**e**) reproducibility of the Ti_2_CT_x_ sensor with 10,000 ppm of CH_4_ (5 cycles); (**f**) effect of relative humidity on the sensor response; (**g**) selectivity of the Ti_2_CT_x_ sensor to CH_4_ over H_2_S and CO with visible-light irradiation; (**h**) long-term stability of the sensor after storage in air. Reprinted with permission from [100]. Copyright 2021: Elsevier B.V.

The application of multilayer Ti_2_CT_x_ MXene, obtained by selective etching of Ti_2_AlC layers with a mixture of LiF and hydrochloric acid without delamination, as a methane sensor operating at RT was demonstrated in a study by Wang and colleagues [100]. They discovered a photocatalytic activity of this compound in the oxidation reaction of CH_4_ under the influence of visible light, which helped them achieve their difficult goal. The authors showed that the rather low response to 1% CH_4_ in darkness, equal to ca. 18.2%, strongly enhances up to ca. 142% when the sensor is illuminated by a visible light from a Xe lamp with a cutoff filter (λ > 420 nm) with a power of 0.85 mW∙cm^−2^ (Figure 6). 

A respective reduction of response time from 136 s to 38 s has been noted, too. It is worth noting that here the humidity also plays an important interference role. When the relative humidity was increased to 60–80%, a decrease in the response to 1% CH_4_ down to ca. 111% was observed. Storage in the air for 90 days led to a decrease in response, down from 130% to 142%. Exposing this MXEne-based sensor to other gases, such as H_2_S and CO, which usually accompany methane in coal mines under their normal concentrations, showed [100] that the selectivity to CH_4_ also enhances under irradiation with visible light. The authors substantiate their idea of the observed photocatalytic activity of Ti_2_CT_x_ by visible light via the complete inactivity of this MXene with respect to methane oxidation in darkness, while CH_4_ is fully oxidized to CO_2_ under illumination. In situ IR spectroscopy measurements of the MXene prior and after viz-light illumination were indicated by the appearance of a characteristic absorption band of CO vibrations at ~2340–2360 cm^−1^, and gas chromatography data that identified CO_2_ at the sensor outlet have been supplied as a confirmation.

**Figure 7:**
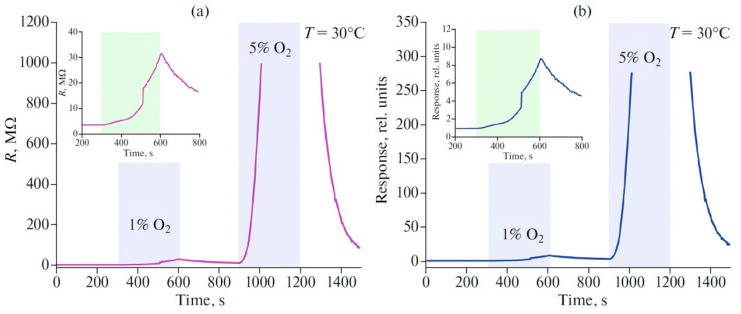
(**a**) Change in the resistance of the receptor material of the Ti_2_CTx coating when puffing gas mixture with 1 and 5% O_2_ and (**b**) the corresponding responses in rel. units /R; operating temperature 30 °C. Reprinted with permission from [101]. Copyright 2022: E.P. Simonenko et al.

In another study [101], a chemiresistive sensor based on few-layer Ti_2_CT_x_ synthesized by etching in NaF + HCl with a subsequent delamination with tetramethylammonium hydroxide and ultrasonic processing exhibited an increased sensitivity to 100 ppm of NO_2_, (a ca. 6% response) at 30 °C. With increasing temperature, however, the greatest response was observed toward 100 ppm ammonia; the response was equal to 13%, with a clear baseline drift. An interesting fact about the reversible detection of oxygen at 1% and 5% concentrations at 30 °C with high sensitivity was found; the corresponding chemiresistive responses were 8.6 and >270 (Figure 7). This opens the prospects for the application of MXene materials, in particular those based on Ti_2_CT_x_, as low-temperature sensors for oxygen.

### 3.3. Features of Gas-Detection Mechanisms in Pristine MXenes

Summarizing the studies presented above, it is necessary to consider the concepts formulated by their authors on a possible mechanism of detection of gaseous analytes, including water vapors, by receptor layers of individual MXenes.

The family of titanium carbide MXenes, such as Ti_3_C_2_T_x_ and Ti_2_CT_x_, is known to have a metallic conductivity while their outer surface is ordinarily coated with polar functional groups. This combination makes this class of compounds attractive for high signal-to-noise ratio (SNR) gas sensors. High SNR, i.e., the ratio of high signal intensity upon gas detection to low electrical noise intensity, is achieved due to the functionalization of the MXenes surface by various groups that provide a strong chemical binding to the analyte gases, as well as the metallic nature of the MXenes’ conductivity, which results in low noise levels [87].

The detection mechanism of MXenes differs significantly from that of semiconductor metal oxide (MOS) materials, which are considered the basic conventional receptors for the whole class of chemiresistive sensors. For MOS-gas sensors, a universal model can be employed to describe the interaction of the analyte gas with ion-adsorbed oxygen and the formation of an electron-depleted layer (EDL) in the near-surface layer of the sensing material [102]. At present, the question of the mechanism of gas detection by MXenes is debatable. Nevertheless, theoretical and experimental models that allow us to describe the appearance of the chemiresistive signal in the atmosphere of various gases have already been found. In general, two possible reasons for the change in resistance, which is positive in the vast majority of cases, are singled out upon gas intake:

(1) Transfer of charge from titanium atoms to adsorbed molecules, supporting a reduction in the transport of charge carriers along MXenes and sometimes their quantity;
(2) Intercalation of gas species into the interlayer space, which causes an enlargement of the distance between the layers and difficulties in the transport of electrons outside the plane of the MXenes.

At the same time, there is a fairly wide range of factors that can influence the response value and kinetic characteristics of MXene materials.

Primarily, the sensitivity of MXenes to various gases is strongly influenced by the functional groups on their surfaces, such as oxygen (–O), hydroxyl (–OH), fluoride (–F), etc., which are defined by a synthesis method. Due to their predisposition to the formation of hydrogen bonds through polar functional groups, MXenes are extremely sensitive to moisture, as noted in Section 3.2 and Section 3.3. Using FT-IR spectroscopy and the study of the wetting angle on the surface of MXenes, Zhang C. et al. found [94] that H_2_O molecules from the gas phase can chemically adsorb on the surface and in the interlayer space of Ti_3_C_2_T_x_ MXenes. The authors proposed a mechanism where the hydroxyl groups appearing on the surface create an electrostatic field and prevent a charge transfer, which explains the positive (*p*-type) response with increasing humidity. The nanoscale interlayer space of ca. 1.01 nm in Ti_3_C_2_T_x_ and the superhydrophilicity of the surface prevent capillary condensation of water and limit moving hydroxyl groups, which are characteristics for bulk nanomaterials. The 2D accordion-like structure that is characteristic for MXenes just after a selective etching of Al from the MAX-phase without a delamination, the metallic type of conductivity, and the fact that H_2_O molecules do not form covalent bonds with the surface of MXenes make them extremely sensitive to humidity and allow for a reproducible and reversible response.

Structural water plays an important role in reducing the overall interaction between the MXene layers and creating additional space for the adsorption and diffusion of gases with affinity to functional groups. This feature is an advantage over other 2D-layered materials, such as, for example, graphene, where strong Van der Waals interactions between 2D sheets and small interlayer distances prevent the intercalation of gas molecules. Koh H-J. et al. demonstrated [89] in situ using XRD that the interlayer space of MXenes contains OH-fragments, such as hydroxyl groups or water molecules, which can be removed when the material is exposed to dry nitrogen. The authors showed that ethanol molecules can chemically bind with Ti_3_C_2_T_x_ MXene in its interlayer space. As a result of these interactions, the MXene swells and its interlayer distance enlarges due to the steric effect. The 2D MXene swollen as a result of ethanol adsorption gets a lower number of electrons involved in a charge transfer between the layers, which leads to the growth of electrical resistance with a positive (*p*-type) chemiresistive response.

MXenes show the best sensitivity when detecting gases capable of forming hydrogen bonds. For instance, Majhi et al. showed [95] the positive (*p*-type) response in the detection of acetone vapors by Ti_3_C_2_T_x_ MXene. They proposed a model of the interaction of acetone molecules with various functional groups on the surface or in the interlayer space of Mxenes. As a result of these interactions, hydrogen bonds are formed. In the course of the reactions on the surface of the MXenes, a significant transfer of the charge carriers takes place, and in the interlayer space—inhibition of conductivity in channels occurs due to the steric effect. In both cases, it is assumed that there is a decrease in the electrical conductivity of the material, which allows a positive *p*-type response to be recorded.

A study by Kim et al. [87] devoted to Ti_3_C_2_T_x_ MXene also proved that the receptor material has the greatest response to gases with a composition and structure capable of forming hydrogen bonds with surface functional groups, such as acetone, ethanol, propanol, and ammonia. At the same time, for acid-forming gases such as NO_2_, SO_2_, and CO_2_, the response is much lower. The authors have demonstrated the ability to detect 50–1000 ppb of acetone, ethanol, and ammonia with a low SNR value. According to DFT calculations, the greatest contribution to the binding of acetone with functional groups on the surface of Ti_3_C_2_T_x_ MXene comes from hydroxyl groups (–OH), while oxygen (–O) and fluoride (–F) substituents are less active. For the –OH groups, the minimum binding energy is significantly lower compared with –O and –F, at −0.774 eV versus −0.317 eV and −0.311 eV, respectively. Thus, the hydroxyl terminal groups on the surface of MXenes likely play a key role in the detection of various gases capable of forming a hydrogen bond.

In an earlier work by Lee et al. [85], another mechanism of interaction of NH_3_ with the functional groups (–O) and (–OH) on the surface of Ti_3_C_2_T_x_ MXene was considered. To explain the positive (*p*-type) response, the interactions of ammonia molecules with the available oxygen-containing functional groups were proposed. As a result of these reactions, the released electrons recombine with holes, which results in increasing the resistance. Unfortunately, the authors do not experimentally confirm in their work the formation of the products of Reactions (12) and (13), which should be desorbed from the surfaces of MXene, N_2_, and NH_2_. Therefore, the described mechanism is not obvious at this time.

Wu et al. [79] studied the responses of Ti_3_C_2_T_x_ MXene to a wide group of gases. The largest positive (*p*-type) response was also recorded toward ammonia. The authors performed DFT modeling of the adsorption of various gas molecules on the surface of the MXenes. It was found that the gas molecules do not dissociate upon adsorption. Therefore, the sorption of gases is purely of a physical nature, which contradicts the model proposed by Lee et al. in [85]. It has been shown that the adsorption energy of acetone and ammonia molecules is approximately the same, to be 0.326 and −0.311 eV. However, taking into account the steric factor, because the volume of the ammonia molecule is much smaller than that of the acetone, additional calculations were performed using Bader charge analysis. It was found that the amount of charge transferred to the ammonia molecule was much greater than for all other test molecules. This can explain the pronounced selective sensitivity of Ti_3_C_2_T_x_ MXene with respect to NH_3_.

The sensory characteristics of Ti_3_C_2_T_x_ and Ti_2_CT_x_ in relation to the technique of their synthesis are summarized in Table 1. It can be stated that experimental and theoretical works have shown that modification of surface functional groups can most significantly affect not only the selectivity but also the value of the response upon the adsorption of gaseous analytes. In some studies, increased response to various gases in the case of non-delaminated, accordion-like MXenes due to their high specific surface area has been noted.

**Table 1:** Sensing characteristics of coatings based on individual Ti_3_C_2_T_x_ and Ti_2_CT_x_ MXenes.

Analyte Gas	Concentration	Operating Conditions	Response (ΔR·100%/R_0_), %	Detection Limit	Synthesis Features	Ref.
Ti_3_C_2_T_x_						
Ammonia	500 ppm	RT	+6.13	10 ppm	NaF + HCl (18–19 wt.%), 60 °C, 48 h, delamination: DMSO, RT, ultrasound for 6 h in nitrogen flow	[79]
Ethanol			+1.5	-		
Methane			+0.5	-		
NO			+0.4	-		
H_2_O			+0.37	-		
Acetone			+0.3	-		
Methanol			+0.2	-		
H_2_S			+0.16	-		
Ammonia	100 ppm	RT	+0.24	25 ppm	LiF + HCl(9M), 35 °C, 24 h, delamination: ultrasound for 30 min	[85]
Methanol			+0.143	-		
Ethanol			+0.115	-		
Acetone			+0.075	-		
Ethanol	100 ppm	RT	+1.7	100–1000 ppb	NaF + HCl (18–19 wt.%), 60 °C, 48 h, delamination: DMSO, RT, ultrasound for 6 h in nitrogen flow	[87]
Acetone			+0.97	50–1000 ppb		
Propanal			+0.88	-		
Ammonia			+0.8	100–1000 ppb		
NO_2_			+0.2	-		
SO_2_			+0.15	-		
CO_2_			+0.12	-		
Ammonia	100 ppm	RT	+17	10 ppm	HF (45 wt.%), 60 °C, 24 h	[88]
NO_2_			+8	-		
Ammonia			+28	-	+NaOH (5M), rinsing	
NO_2_			+11	-		
Ethanol	0.1%	RT	+0.8	-	LiF + HCl(9M), 35 °C, 24 h, delamination: ultrasound 1 h	[89]
Ethanol	0.1%		+10	-	+NaOH (0.3 mM)	
Ammonia	100 ppm	RT	+0.9	-	LiF + HCl(9M), 35 °C, 24 h, delamination: ultrasound 1 h	[90]
Ethanol			+0.8	-		
Ammonia			+3.8	10 ppm	+KOH (0.01M)	
Ethanol			+3.4	-		
Ammonia			+0.4	-	+KCl (0.01M)	
Ethanol			+<0.5	-		
Toluene	50 ppm	RT	+19.32	-	LiF + HCl(9M), 35 °C, 24 h, delamination: ultrasound 1 h	[91]
Toluene			−79.5	500 ppb	Ti_3_C_2_T_x_ + thiourea, 500 °C, Ar, 3 h	
Ethanol	120 ppm	RT	+6.6	-	LiF + HCl(9M), 35 °C, 24 h	[92]
Ethanol			+10.1	-	+(3-chloropropyl)-trimethoxysilane (5%), 24 h	
Ethanol			+14	5 ppm	+1H,1H,2H,2H-perfluorooctyltriethoxysilane (5%), 24 h	
Ethanol	40 ppm	RT	+5	-	LiF + HCl(9M), 40 °C, 24 h, delamination: ultrasound 30 min	[93]
Octane			+0.3	-		
Toluene			+0.1	-		
H_2_O			+2	-		
Ethanol			+6.2	20 ppm	+DMSO, trimethylacetic anhydride, 40 °C, 36 h	
Octane			+0.9	-		
Toluene			+0.8	-		
H_2_O			+1.3	-		
Ethanol			+8	-	+1) NaHCO_3_ (1M), +2) DMSO, trimethylacetic anhydride, 40 °C, 36 h	
Octane			+2.5	-		
Toluene			+1.4	-		
H_2_O			+0.6	-		
H_2_O	RH = 33%	RT	+7	-	HF (40 wt.%), 40 °C, 36 h	[94]
H_2_O	RH = 67%		+10	-		
H_2_O	RH = 95%		+15	-		
Acetone	100 ppm	23 °C	+99	-	HF (48 wt.%), 60 °C, 15 h	[95]
	250 ppb		+17.3	-		
Ti_2_CT_x_						
Ammonia	10 ppm	RT	+1.1	-	HF (20%), RT, 12 h, delamination: DMFA, ultrasound 12 h	[99]
Ammonia	10 ppm		+0.7	-	LiF + HCl(12 M), 40 °C, 24 h, rinsed with HF (10%)	
Methane	200 ppm	RT, visible light irradiation	+16	-	LiF + HCl(9M), 35 °C, 36 h	[100]
Methane	1000 ppm		+68	-		
Methane	10,000 ppm		+142	-		
NO_2_	100 ppm	30 °C	+6	-	NaF + HCl(6M), 40 °C, 24 h, delamination: tetramethylammonium hydroxide, ultrasound 30 min	[101]
Ammonia	100 ppm	50 °C	+13	-		
Oxygen	1%	30 °C	+8.6 *	-		
Oxygen	5%		>276 *	-		

* SO_2_ = R/R_0_ [101].

## 4. Nanocomposites Based on Ti_3_C_2_T_x_ and Ti_2_CT_x_ MXenes

While there are undeniable advantages of MXenes as receptor materials for gas sensors, primarily the possibility to operate at RT and to consume a lowered power, there are also serious negative factors that greatly complicate the application of Ti_3_C_2_T_x_ and Ti_2_CT_x_ layers. For instance, when applying coatings, it is inherent to MXenes to agglomerate, to self-assemble into fairly dense stacks of monolayers, which leads to a sharp reduction in the specific surface area and, consequently, to the deterioration of sensitivity. Furthermore, due to the large interaction energy of the Ti_3_C_2_T_x_/Ti_2_CT_x_ surface with molecules of gaseous analytes, as well as the need for diffusing gases into the interlayer space of MXenes, they have overly long sensor response and recovery times, up to tens of minutes, which often exceed these parameters for commercial MOS sensors.

High reactivity, in particular, when interacting with oxygen in a humid environment, typical for nano-dispersed carbides and metal nitrides, leads to constant and fairly fast degradation of MXenes. Therefore, there is a problem for sensors based on Ti_3_C_2_T_x_ and Ti_2_CT_x_ to perform steadily within even just a few days. Another experimental problem may be the non-reproducibility of the properties of the receptor materials from batch to batch due to contamination by various synthesis by-products and different storage times of the starting substance prior to applying the receptor layers. Furthermore, the high surface hydrophilicity of MXenes, on which electronegative substituents –F, –OH, and –O are localized, causes their high sorption capacity toward H_2_O molecules, which is associated with prospects to employ these layers as humidity sensors, as discussed in Section 3. However, this is a negative factor for the detection of other gaseous analytes, including VOCs, ammonia, and other inorganic gases, because a change in relative humidity in the vast majority of cases causes a significant modulation of the response value.

To overcome these drawbacks, it is proposed to apply approaches known from experience with MOS sensors. In particular, the most powerful one is designing nanocomposites involving semiconducting metal oxides and chalcogenides, nanoparticles of noble metals, carbon materials such as graphene and nanotubes, and polymeric materials. The modification of the surface of MXene sheets by such components creates steric difficulties for the formation of dense aggregates; at least, it dramatically enhances the interlayer distance. In some cases, it leads to the appearance of hierarchically organized mesoporous 3D structures with a much larger specific surface area compared with individual MXenes. In addition, more adsorption centers are induced. The heterojunctions yielded between semiconductor and/or metallic nanoparticles and MXene layers frequently stimulate and advance the chemiresistive response. The combination of the positive characteristics of components of different natures often causes a synergistic effect and a significant change in the gas-detection mechanism of MXenes. Still, we leave out here the finer approaches related to the replacement of various atoms in the MXene crystals by foreign ones [27].

Interestingly, only one publication involving the Ti_2_CT_x_ phase was found in the body of work devoted to the use of nanocomposites with carbide MXenes in chemiresistive gas sensors.

Table 2 provides a short summary of the sensory properties of nanocomposite materials based on Ti_3_C_2_T_x_ and Ti_2_CT_x_ in relation to the synthesis method.

### 4.1. Modification of Ti_3_C_2_T_x_ and Ti_2_CT_x_ by Metal Oxide Semiconductors

#### 4.1.1. Modification with *n*-Type Metal Oxide Semiconductors

Non-stoichiometric metal oxides of *n*-type conductivity are the most known ones to be employed in current commercial chemiresistors. Primarily, these are TiO_2_, SnO_2_, and ZnO to consider employing as additives to MXene structures. In this subsection, we look sequentially at how these metal oxide/MXene structures were elaborated.

**Figure 8:**
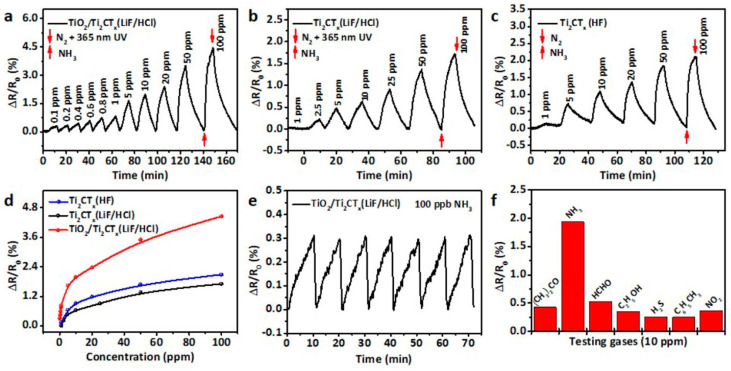
Dynamic response/recovery curves of sensors fabricated from (**a**) TiO_2_/Ti_2_CT_x_ (LiF/HCl) nanosheets, (**b**) Ti_2_CT_x_ (LiF/HCl) nanosheets, and (**c**) Ti_2_CT_x_ (HF) nanosheets in response to NH_3_ gas with increasing concentration at RT (e.g., 25 °C). (**d**) Normalized change of resistance of different sensors at various NH_3_ concentrations. (**e**) Response/recovery curve for seven successive cycles of exposure to 100 ppb NH_3_ gas. (**f**) Response of TiO_2_/Ti_2_CT_x_ (LiF/HCl) nanosheets upon exposure to 10 ppm of (CH_3_)_2_CO, NH_3_, HCHO, C_2_H_5_OH, H_2_S, C_6_H_5_CH_3_, and NO_2_ at RT. Reprinted with permission from [99]. Copyright 2020: The Royal Society of Chemistry.

**TiO_2_**. Modification of Ti_3_C_2_T_x_-based receptor materials with titanium dioxide is most common, primarily because the inevitable oxidation of these MXenes yields nano-dispersed, often amorphous, TiO_2_. Already in this case, the improved sensory properties of the resulting composite material are noted. Thus, a sample of Ti_2_CT_x_/TiO_2_ composite formed by incubation of MXenes obtained under the influence of LiF/HCl in an ethanol-water mixture at 4 °C for 16 h [99] was characterized by weak *n*-type semiconductor properties, close to rutile. The authors showed that the initial receptor material of Ti_2_CT_x_ possessed high sensitivity to NH_3_, while the sensor based on the Ti_2_CT_x_/TiO_2_ composite allowed one to detect even 100 ppb of NH_3_, as given in Figure 8. Moreover, the response value at 100 ppm ammonia for this sensor exceeds that of individual Ti_2_CT_x_ MXenes obtained by etching with HF acid and LiF/HCl by almost three and two times, respectively [99]. While studying Ti_2_CT_x_/TiO_2_ against a number of other VOCs, including hydrogen sulfide and nitrogen dioxide (10 ppm), it was found that the ratio of responses to ammonia and other gases was at least higher than ca. 3.8, which is characteristic for the second most sensitive gas, formaldehyde. It indicates rather high selectivity to NH_3_. Moreover, with enhancing the degree of Ti_2_CT_x_ oxidation by longer time soaking in an ethanol-water mixture, up to 12 h, and increasing the TiO_2_ nanoparticle content, the sample response to ammonia has been raised, but after 16 h of oxidation the response dropped due to a destruction of the original MXene sheet into porous and smaller fragments. The authors suggested that the appearance of TiO_2_ nanoparticles on Ti_2_CT_x_ increases the number of adsorption centers and adjusts the film resistance in the range of 10^4^–10^7^ Ohm, optimal for reading by electronic circuits.

**Figure 9:**
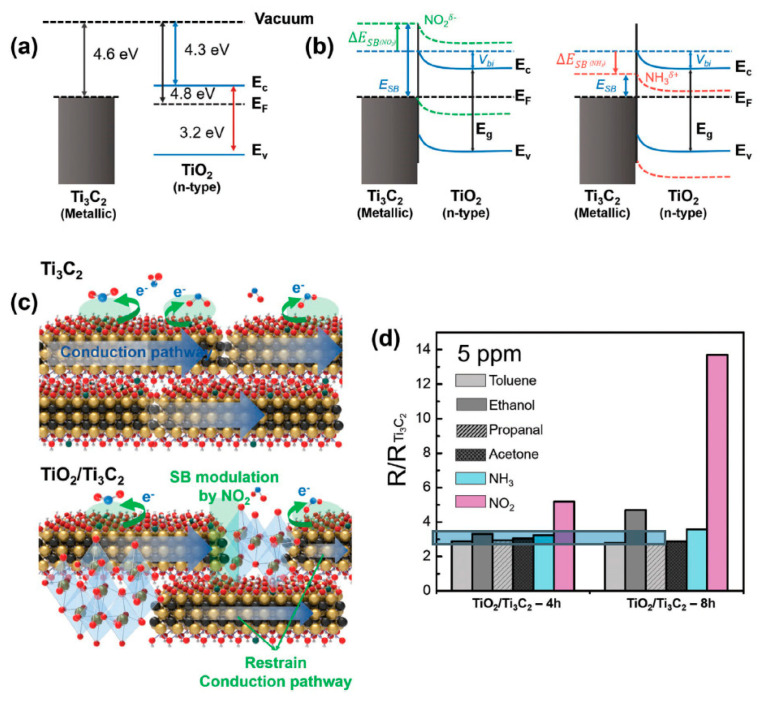
(**a**) A schematic illustration of work function and Fermi level position for Ti_3_C_2_ metallic channel and TiO_2_ semiconductor. (**b**) Schottky barrier modulation upon exposure to NO_2_ and NH_3_ gases. (**c**) Gas-sensing mechanism toward NO_2_ gas for Ti_3_C_2_ and TiO_2_/Ti_3_C_2_ films. (**d**) Enhancement factor of gas response for TiO_2_/Ti_3_C_2_ compared with pristine Ti_3_C_2_ at 5 ppm gas concentration. Reprinted with permission from [103]. Copyright 2020: Wiley-VCH GmbH.

Choi et al. studied [103] the gas-sensitive properties of the Schottky barrier that appeared at the interface between the metal-like MXene Ti_3_C_2_T_x_ and the semiconductor titanium oxide that was generated by holding the MXene in an aqueous dispersion at 80 °C for 4–48 h. This method of Ti_3_C_2_T_x_ oxidation was investigated to obtain the most uniform distribution of the oxide because the oxidation degree for the surface layers is higher than for the inner ones when oxidizing the films that have already been deposited. The authors have elucidated the maximum time for such an oxidation to be around 8 h; the longer times resulted in the oxide phase predominating over the MXene one. At this optimum oxidation time of 8 h, TiO_2_ nanoclusters have been grown on the edges of Ti_3_C_2_T_x_ sheets. Such a structure exhibited a 260-fold growth of the electrical resistance when compared with the pristine MXene, but also an advanced chemiresistive response of the *p*-type toward a few VOC analytes, 5 ppm of toluene, ethanol, propanol, acetone, and toxic gases, 5 ppm of NO_2_ and NH_3_, at RT. Such a hybrid Ti_3_C_2_T_x_/TiO_2_ structure sample yielded the enhanced chemiresistive response by 13.7 times to NO_2_, by 4.7 times to toluene, and ca. three times for the other gases. At the same time, a significant drift of the baseline was observed, which was most likely related to the long recovery time of the sensor. The authors suggested a possible detection mechanism for this heterostructure, taking into account the determined work functions and Fermi levels of their components and constructing the band structure of the nanocomposite (Figure 9), which affects mainly the NO_2_ detection mechanism. It was revealed that the response value to the analyte reducing gases does not almost change with varying the time and, accordingly, the degree of oxidation of Ti_3_C_2_T_x_, which has been explained by the formation of the interlayer structure of the Ti_3_C_2_T_x_/TiO_2_ composite [103].

**Figure 10:**
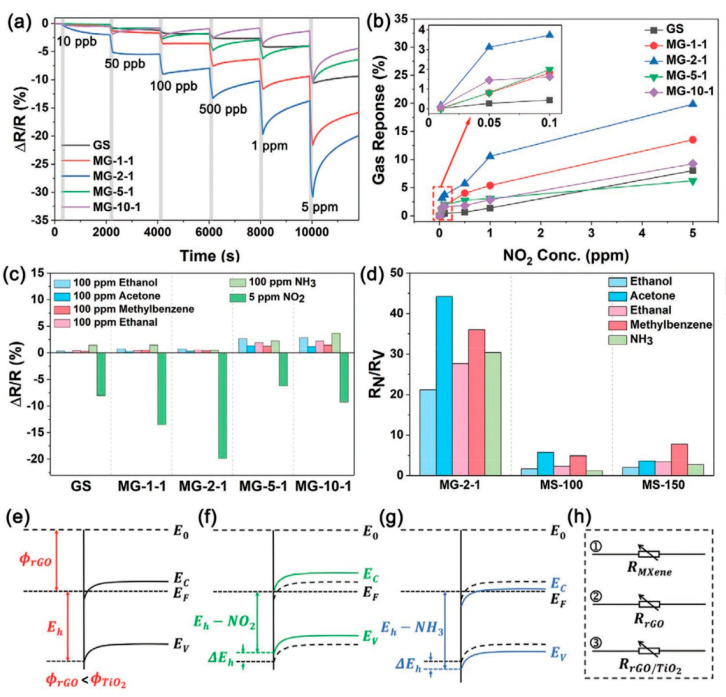
(**a**) The dynamic response/recovery curve of sensors based on GS, MG-1-1, MG-2-1, MG-5-1, and MG-10-1 in the different concentrations of dry NO_2_. (**b**) The gas response curve of sensors based on GS, MG-1-1, MG-2-1, MG-5-1, and MG-10-1 depending on dry NO_2_ concentration. (**c**) The maximum resistance change rate of sensors based on GS, MG-1-1, MG-2-1, MG-5-1, and MG-10-1 to 100 ppm of dry ethanol, acetone, ethanal, methylbenzene, NH_3_, and 5 ppm of dry NO_2_ within two minutes of exposure. (**d**) The response ratio (R_N_/R_V_ × 100%) of sensors based on MG-2-1, MS-100, and MS-150 to 5 ppm dry NO_2_ (R_N_) relative to various 100 ppm other dry gases (R_V_). (**e**) The schematic illustration of energy band of rGO/TiO_2_ contact. The change of energy band of rGO/TiO_2_ heterojunction after exposure to (**f**) NO_2_ and (**g**) NH_3_. (**h**) A schematic illustration of the mechanism of gas-sensing response of MGs. Reprinted with permission from [104]. Copyright 2021: Wiley-VCH GmbH.

Yang and colleagues [104] raised objections to the detection mechanism proposed in [103] based on Schottky barrier formation because there should be observed an ohmic transition at the Ti_3_C_2_T_x_/TiO_2_ heterojunction upon a certain work function for the *n*-type TiO_2_ semiconductor that exceeded that for Ti_3_C_2_T_x_. An additional argument by Yang et al. against the signal upon the NO_2_ detection amplification mechanism proposed by the authors of [103] is that the response is preserved with increasing resistance, regardless of whether the oxidizing gas or the reducing gas are adsorbed. It is likely that the resolution of this question directly relates to the processes involved in the formation of TiO_2_ nanoparticles. For sensor experiments, crumpled spheres containing atomic Ti defects and Ti_3_C_2_T_x_/TiO_2_ heterojunctions and samples of similar morphology containing only Ti_3_C_2_T_x_/TiO_2_ heterojunctions [104] were fabricated via ultrasonic sputtering pyrolysis technology. The temperature of heat treatment in air, in the 100–200 °C range, regulated the number of atomic defects of Ti and TiO_2_. To create a real Schottky heterojunction, graphene oxide was also introduced into the material composition, and its reduction was performed by in situ oxidation of Ti_3_C_2_T_x_, giving the appearance of a more complex composition and structure in the Ti_3_C_2_T_x_/TiO_2_/rGO composite. The performed gas sensitivity measurements enabled us to show that there was no increase in the NO_2_ response when only TiO_2_ appeared on the MXene surface. However, if titanium atoms from Ti_3_C_2_T_x_ layers have been involved in building titanium dioxide and, accordingly, atomic deficiencies have been formed, the response to NO_2_ goes up significantly, especially at the oxidation temperature of 150 °C, from ca. 11.8% to ca. 26.7%. Realization of the real Schottky heterojunction in the synthesis of Ti_3_C_2_T_x_/TiO_2_/rGO nanocomposite resulted in a negative response to NO_2_, with positive and relatively low responses to other gases, which is especially remarkable for the sample obtained with a 2:1 mass ratio of Ti_3_C_2_T_x_ to GO (Figure 10). The authors also formulated ideas about the detection mechanism of Ti_3_C_2_T_x_/TiO_2_/rGO composites involving the Schottky barrier at the rGO/TiO_2_ interface.

**Figure 11:**
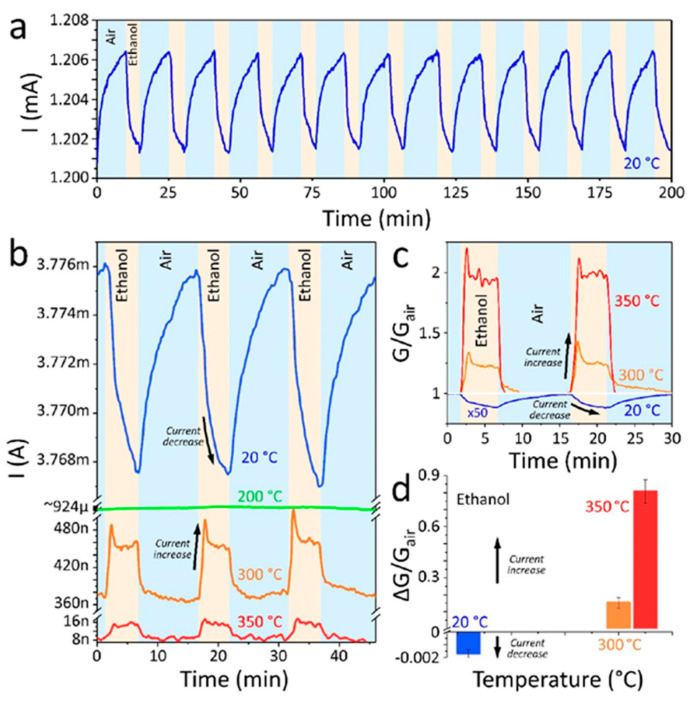
Sensor behavior of Ti_3_C_2_T_x_ MXene annealed at different temperatures. All panels show sensor responses to 250 ppm ethanol. The voltage applied between the device contacts was 1V. (**a**) Stability of sensor responses of pristine Ti_3_C_2_T_x_ MXene measured at RT (20 °C). (**b**) Responses of a MXene sensor element that was measured at RT (20 °C; blue curve) and then annealed at 200 °C (green curve), 300 °C (orange curve), and 350 °C (red curve). After each annealing, the sensor measurements were performed at the annealing temperature. (**c**) Normalized sensor responses at different temperatures for the same MXene sensor element. The responses for the pristine metallic MXene are multiplied by 50 to be visible on the figure scale. (**d**) Comparison of sensor responses at different temperatures for the same MXene sensor element. Reprinted with permission from [105]. Copyright 2020: American Chemical Society.

One of the most frequently used methods of partial oxidation of Ti_3_C_2_T_x_ in order to increase the response value due to the appearance of highly dispersed TiO_2_ particles is thermal oxidation, primarily in an oxygen-containing atmosphere [29,105,106]. It was shown [105] that a partial oxidation in air of the Ti_3_C_2_T_x_ MXene layer at 350 °C, to be conducted in situ on the sensor chip unit, resulted in high responses of ca. 40–180%, with fast response and recovery rates for a number of VOCs down to 2 ppm concentration at a fairly high temperature of 350 °C, which was significantly different from the behavior of the initial Ti_3_C_2_T_x_. The unoxidized material was characterized by responses to VOC vapor intake with an increase in resistance at an operating temperature of 20 °C, while the Ti_3_C_2_T_x_/TiO_2_ composite, which contained titanium dioxide in two modifications, anatase and rutile, exhibited a decrease in resistance, which is more typical for *n*-type MOS-based sensor behavior. The authors showed that the oxidation temperature significantly affects the sensor properties: at temperatures of 100–200 °C, a calcination of Ti_3_C_2_T_x_ film has not yielded any response even to 250 ppm of ethanol, while at temperatures of 300–350 °C, the coating resistance has shown a remarkable reduction in the presence of ethanol vapors (Figure 11). At the same time, for the Ti_3_C_2_T_x_/TiO_2_ nanocomposite at RT in the presence of alcohols, responses with resistance increases were still observed, which the authors attributed to reactions in internal regions of the multilayer coating with the preserved MXene structure.

Discussing the detection mechanism given in [105], and based on the measured yields for the original Ti_3_C_2_T_x_ and the Ti_3_C_2_T_x_/TiO_2_ nanocomposite, it is assumed that a potential barrier of ~1 eV is formed between the components. Electrons from TiO_2_ nanoparticles enter the metal channel of MXene, while the reverse diffusion is prevented by the potential barrier. Given the particle size and the Debye length of non-stoichiometric TiO_2_, the resulting clusters are expected to be completely depleted of electrons. Thus, the reduced conductivity of Ti_3_C_2_T_x_/TiO_2_ films is explained by the fact that electron-depleted TiO_2_ clusters form potential barriers between the electrically conductive MXene sheets. When analyte molecules are adsorbed, they supply electrons to TiO_2_ nanoparticles, which causes a decrease in the potential barrier and a corresponding increase in the conductivity of the material as a whole.

Because the response to all of the gaseous analytes under study occurred with decreasing resistance and was close in magnitude, the device was integrated in a multielectrode matrix equipped with 38 pairs of independent electrodes to improve selectivity. Processing the obtained vector signals from all the sensors on the chip using linear discriminant analysis (LDA) made it possible to reliably recognize even analytes that were close in chemical nature, including low-molecular-weight alcohols (methanol, ethanol, and isopropanol) [105].

The results of the surface oxidation of Ti_3_C_2_T_x_ by oxygen plasma at temperatures of 350–550 °C are presented in another work [107]. In order to obtain modified TiO_2_ samples, an accordion-like MXene coating was synthesized under the influence of a lithium fluoride solution in concentrated hydrochloric acid and placed into the MPCVD reactor, where plasma was generated in an O_2_ atmosphere at a residual pressure of 2 kPa using microwave radiation. At the same time, the substrate temperature was varied from 350 °C to 550 °C.

It was found that the Ti_3_C_2_T_x_ phase is preserved in all samples, in addition to the formation of anatase and defects, and that with increasing the activation temperature of the MXene, the number of surface F-groups reduces. It was also noted that a systematic growth of the film resistance from the kOhm to MOhm range is observed as the exposure temperature goes up. By varying the operating temperature in the range of 250–400 °C, the authors found that the sensitivity to ethanol is highest at 325 °C; the sensor being plasma-activated at 500 °C exhibited the greatest response, which was 34.7% higher than that of original Ti_3_C_2_T_x_. This result was explained by increasing the number of –O functional groups and defects in the MXene layers under exposure to O_2_ plasma, which, in turn, provided more oxygen adsorption centers. Moreover, the room-temperature response to ethanol for the oxidized sample was found to be almost twice as high as for the original MXene, at ca. 22.5% compared with ca. 13.3%. Still, the response was of the *n*-type and related to resistance reduction. The chemiresistive response was rather stable within 10 days of continuous testing. When explaining the mechanism of VOC detection in the material, the authors rely on noting the interaction of analyte molecules with ion-adsorbed oxygen, which yields an electron-depleted layer on the surface of the particles of the receptor material, an *n*-type Ti_3_C_2_T_x_ semiconductor. The treatment of MXene with oxygen plasma in an oxygen-rich gas atmosphere causes reactions between hydroxyl groups with the production of H_2_O and the removal of fluoride groups from the surface. These processes lead to an increase in the number of functional O-groups and promote the adsorption/desorption of gas molecules to improve sensitivity. In addition, the higher number of O-groups on the surface of MXene layers provides an opportunity to raise the work function.

In order to improve the response of Ti_3_C_2_T_x_ MXene to ethanol vapors at RT, the receptor material was also treated in another study [108] with oxygen plasma, which led to the appearance of titanium dioxide on the surface. The authors attributed the observed hundred-fold growth of response and improvement of the sensor kinetic characteristics to the lattice distortion, the formation of a mesoporous structure, and the presence of a Schottky barrier at the Ti_3_C_2_T_x_-TiO_2_ interface.

To further improve the moisture sensitivity on the surface of Ti_3_C_2_T_x_ layers, TiO_2_ nanowires were grown [109]. For this purpose, the Ti_3_C_2_T_x_ MXene powder obtained by treating the MAX-phase with concentrated HF after delamination in N-methylpyrrolidone was introduced into a KOH solution and heated at 50 °C. It was found that this resulted in the growth of Ti_3_C_2_T_x_ nanowires of 10–100 nm in diameter and 500–1000 µm in length, depending on KOH concentration and duration of exposure, by diminishing fluoride functional groups on the surface of Ti_3_C_2_T_x_ nanosheets. The whole structure was shown to have the specific surface area to increase from 10.8 m^2^/g to 52.6 m^2^/g. As a result, the sensitivity of the prepared Ti_3_C_2_T_x_/TiO_2_ nanocomposite to humidity was high, which was explained via complex impedance spectroscopy and Schottky transition theory.

**Figure 12:**
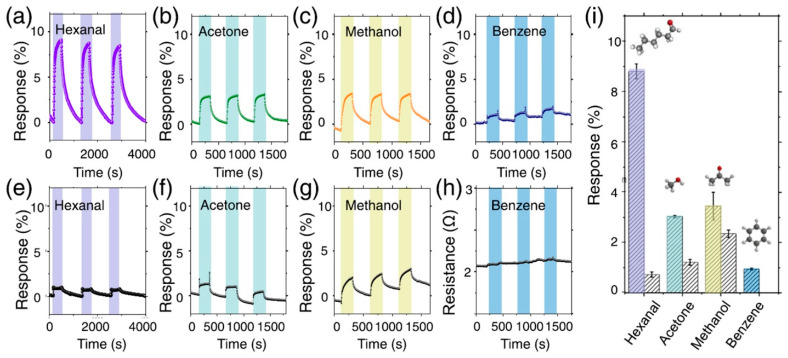
Normalized real-time responses of the Ti_3_C_2_T_x_-TiO_2_ nanocomposites sensor to 100 ppm (**a**) Hexanal, (**b**) Acetone, (**c**) Methanol, and (**d**) Benzene in 3 successive cycles. Normalized real-time responses of the Ti_3_C_2_T_x_ MXene sensor to 100 ppm (**e**) Hexanal, (**f**) Acetone, and (**g**) Methanol. (**h**) Resistance variation of the sensor upon exposure to 100 ppm of Benzene in 3 successive cycles. (**i**) Selectivity of the sensor to various gaseous VOCs at RT. Reprinted with permission from [110]. Copyright 2021: Elsevier B.V.

The application of Ti_3_C_2_T_x_ MXene as a source of titanium oxide in the hydrothermal synthesis of Ti_3_C_2_T_x_/TiO_2_ nanocomposites as a receptor material for the detection of VOCs has been considered by Kuang et al. [110]. For this purpose, Ti_3_C_2_T_x_ MXene powder synthesized by etching the Ti_3_AlC_2_ with concentrated hydrofluoric acid was dispersed in a 50% ethanol solution and then subjected to hydrothermal treatment at 200 °C for 25 h. An XRD study revealed that such processing resulted in (i) forming an additional anatase phase and (ii) enlarging the inter-planar distance as observed as a shift in the 002 reflex from 9.19° to 9.02° due to the appearance of oxide nanoparticles. The measurement of the specific surface area for the Ti_3_C_2_T_x_/TiO_2_ nanocomposite gave a value of 81.9 m^2^/g, which is an order of magnitude higher than that for the original Ti_3_C_2_T_x,_ of 8.6 m^2^/g. At the same time, the MXene layers were uniformly covered by globular nanoparticles with a diameter of approx. 30–50 nm. These composite samples were found sensitive to VOCs, particularly hexanal, in the 10–40 ppm concentration range, with the responses being 5–6 times higher than those of the original Ti_3_C_2_T_x_ (Figure 12). Moreover, both samples of the receptor materials exhibited *p*-type responses, indicating a decrease in the number of major carriers or blockage of carrier transport under the adsorption of gaseous analytes. It is noted that some baseline drift and high response and recovery times, 293 s and 461 s, respectively, were observed for Ti_3_C_2_T_x_/TiO_2_ in signal reproducibility experiments versus 10 ppm of hexanale. The promising application of the synthesized Ti_3_C_2_T_x_/TiO_2_ composite for disease detection by exhalation analysis has been demonstrated, since the very low sensitivity of the sensor to CO_2_ can reduce its negative effect on the detection of VOC markers. The increase in humidity in the background air from 0 to 80 rel.% forces a reduction in the response to hexanal, 10 ppm as an example, linearly from ca. 2.8% to ca. 0.7%, in accordance with observations about competition between H_2_O molecules and hexanal ones at the sensor surface for adsorption centers. The kinetic characteristics of Ti_3_C_2_T_x_/TiO_2_ are significantly improved at RH = 20%. The authors cited [110] a combination of three key factors as reasons for the advanced sensitivity of the Ti_3_C_2_T_x_/TiO_2_ nanocomposite: (1) the preservation of electrical properties of the multilayer 2D metal framework of MXene provides a fast charge carrier transfer at RT, which underlies the observed high SNR; (2) the advanced specific surface area significantly improves the adsorption of VOC molecules that, in turn, facilitates a greater response value; and (3) the strong electronic interaction between the surfaces of Ti_3_C_2_T_x_ and TiO_2_ due to heterojunctions can modulate the charge transfer upon test gas exposures.

**Figure 13:**
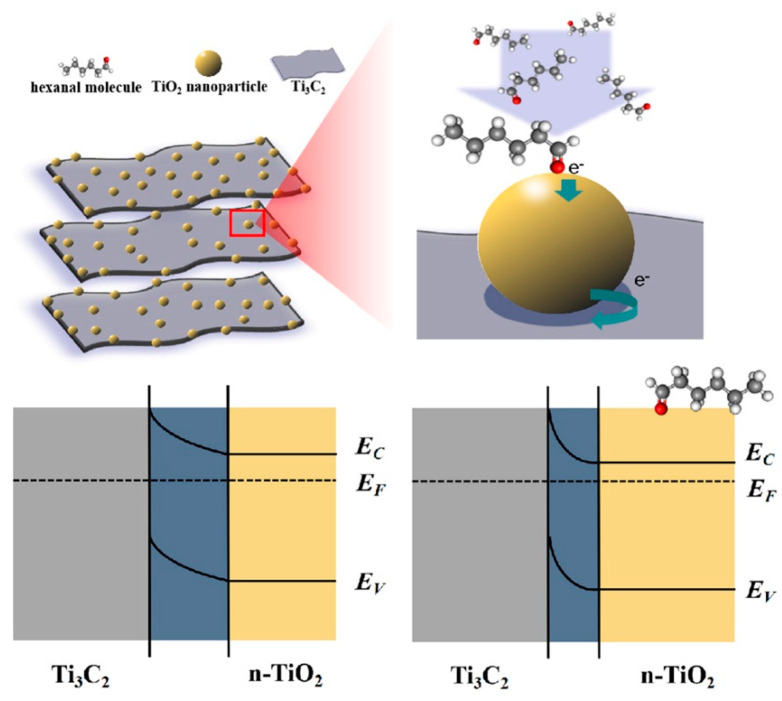
Schematic illustration of the sensing mechanism of the TiO_2_-Ti_3_C_2_ nanocomposite for hexanal and the proposed energy band structure diagram. Reprinted with permission from [110]. Copyright 2021: Elsevier B.V.

Thus, the formation of TiO_2_ nanoparticles will produce crystal defects in the anion positions, for each of which the two Ti^4+^ cations in the crystal lattice nodes are reduced to Ti^3+^ to maintain electrical neutrality, forming an oxygen vacancy with two positive charges [110]. When a hexanal molecule is adsorbed, these vacancies should tend to capture its oxygen atom and the corresponding electrons, i.e., TiO_2_ defects act as the major adsorption centers. The migration of electrons from the conduction zone of TiO_2_ to the Ti_3_C_2_T_x_ MXene leads to a depleted layer at their interface and a Schottky barrier, which is associated with the higher resistivity of the Ti_3_C_2_T_x_/TiO_2_ composite; see Figure 13. The absorption of hexanal molecules on the TiO_2_ surface containing oxygen vacancies and the transfer of electrons to it will lead to a change in the Fermi levels and a decrease in the thickness of the free carrier-depleted layer. These processes result in a reduction of the concentration of hole carriers in the basic planes of the MXene and force the material resistance to rise. In general, the authors confirm that the continuous heterogeneous Ti_3_C_2_T_x_/TiO_2_ interface and reduced charge carrier density are responsible for the high SNR and improved gas response [110].

In work by Liu et al. [111], it was found that the temperature of the hydrothermal synthesis of Ti_3_C_2_T_x_/TiO_2_ nanocomposite is a key factor in improving the sensory characteristics. The materials were synthesized by oxidation of the initial Ti_3_C_2_T_x_ MXene obtained using concentrated hydrofluoric acid under hydrothermal conditions at temperatures of 140–220 °C for 12 h. It is shown that the number of TiO_2_ nanoparticles growing in situ on the surface of multilayer MXene sheets increases as the processing temperature goes up, while their size enlarges until the temperature rises to 180 °C and is further stabilized. At the same time, the response to NO_2_, 100 ppm, has gradually increased from ca. 2.2 to ca. 4.5 with the advancement of the hydrothermal oxidation temperature from 140 °C to 180 °C; for comparison, the response of unoxidized Ti_3_C_2_T_x_ was equal to ca. 0.3. Still, no significant change in the response value and kinetic parameters occurred with further increases in the hydrothermal treatment temperature [111]. In this regard, the authors chose a temperature of 180 °C as the optimum one for the hydrothermal synthesis of Ti_3_C_2_T_x_/TiO_2_ nanocomposite. This material exhibited a response vs. NO_2_, 100 ppm, that was ca. 2.1 times greater than that for 100 ppm of SO_2_, 6.3 times greater than that for 100 ppm of NH_3_, and 6.7 times greater than that for 100 ppm of CO, which indicates quite good selectivity with respect to NO_2_.

**Figure 14:**
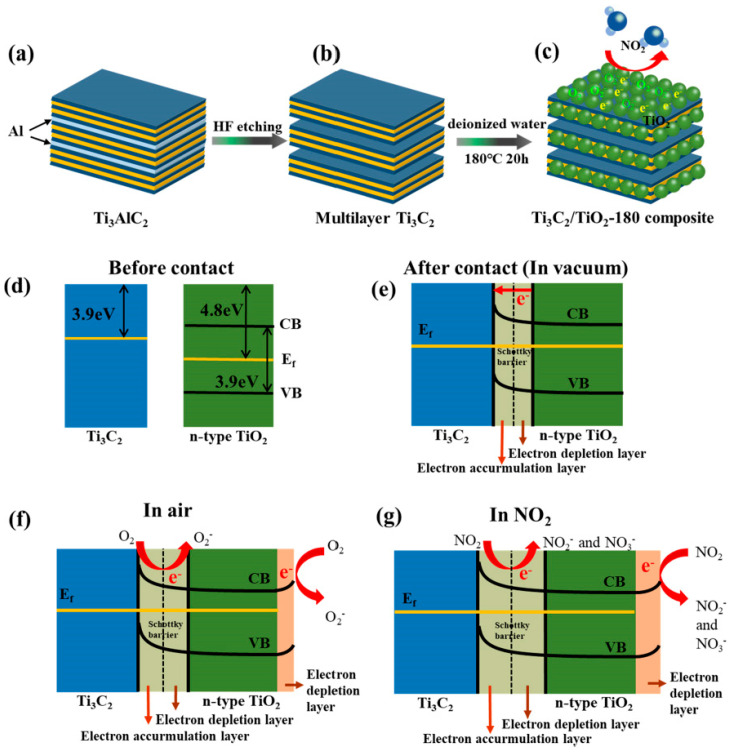
(**a**–**c**) Schematic illustration of the preparation process of Ti_3_C_2_/TiO_2_ composite; (**c**) Adsorption model of NO_2_ gas; (**d**) Band structures of Ti_3_C_2_ and TiO_2_ before contact; (**e**–**g**) Variation of Schottky barrier of Ti_3_C_2_/TiO_2_ composite under different conditions. Reprinted with permission from [111]. Copyright 2021: Elsevier B.V.

The higher operating temperatures allowed the sensor to advance the response to 100 ppm of NO_2_; at the optimal operating temperature of 175 °C, it was ca. 19.8 when compared with about 4.5 at RT. In addition, the expected decrease in response and recovery times was observed with higher detection temperatures. It is worth noting that in describing the mechanism of NO_2_ detection by the Ti_3_C_2_T_x_/TiO_2_ nanocomposite, the authors rely on data from the literature [103,112]. In contact with air, the adsorbed oxygen molecules on the surface of the MXenes can change into the anionic form (O_2_^−^), which leads to the growth of the Schottky barrier and the formation of an electron-depleted layer at the surface of TiO_2_ nanoparticles, as shown in Figure 14. When NO_2_ molecules are collected on the surface of Ti_3_C_2_T_x_/TiO_2_ composite, they accept electrons according to two possible Reactions (14) and (15) given below, which are accompanied by a deformation of the band structure and an increase in the resistance.

NO_2(g)_ + ē → NO_2_^−^ _(ads)_ (14)

2NO_2(g)_ + O_2_^−^ + ē → 2NO_3_^−^ _(ads)_ (15)

Overall, the research published in [111] shows that the choice of the optimal temperature not only for the hydrothermal oxidation of Ti_3_C_2_T_x_ but also for the sensor operating temperature makes it possible to improve the sensor characteristics of the MXene.

In a study by Zhang and coworkers [113], a (001)-oriented TiO_2_ was grown on 2D Ti_3_C_2_T_x_ carbide using a hydrothermal method to develop a highly sensitive and selective sensor for NH_3_ as a food spoilage degree marker. To form predominantly the (001) plane, Ti_3_C_2_T_x_ multilayer MXene powder obtained by HF in a 50% solution was mixed with an aqueous NaBF_4_ solution and hydrothermally treated at 160 °C for 8–32 h. With increasing the time of heat treatment, the number of TiO_2_ nanosheets raised to be located laterally to the MXene planes. At the same time, the 32 h treatment results in the destruction of the layered accordion-like structure of MXene and the formation of disordered aggregates consisting of TiO_2_ particles. Exposure to ammonia in a concentration range of 0.05–30 ppm forced the nanocomposites to reduce a resistance that is rather unusual for the pristine Ti_3_C_2_T_x_ MXene, similar to the response of *n*-type semiconductors.

The sample of Ti_3_C_2_T_x_/TiO_2_ derived after 16 h of hydrothermal treatment demonstrated the best sensitivity to ammonia; its sensitivity to 10 ppm of NH_3_ was seven times higher than that of Ti_3_C_2_T_x_. Its selectivity to ammonia compared with similar amounts of typical gaseous reducing analytes (formaldehyde, trimethylamine, methanol, ethanol, benzene, CO, H_2_S) and oxidants (NO_2_, SO_2_, CO_2_) was noted. 

An additional UV activation of the sensors has further increased the sensitivity of this Ti_3_C_2_T_x_/TiO_2_ nanocomposite to NH_3_ by 2–5 times and reduced its detection limit to 5 ppb. The sensor based on the Ti_3_C_2_T_x_/TiO_2_ nanocomposite obtained after 12 h of hydrothermal treatment exhibited a stable response at background humidity varying from 25 rel.% to 67 rel.%, which is the most frequently occurring in practice. Implemented into the alarm system designed by the authors, the material was found promising to indicate the beginning of decomposition of pork, fish, and shrimp [113].

The detection of trace amounts of NH_3_ at RT was also the goal of work by Zhou et al. [114]. For this purpose, the oxidation of multilayer Ti_3_C_2_T_x_ MXene produced using LiF solution in hydrochloric acid was performed simultaneously with its functionalization with nitrogen via a solvothermal treatment at 180 °C of its suspension in a mixture of ethanol-ethylene glycol solvent with dissolved urea. As the duration of treatment increased, anatase nanoparticles were observed to grow as a result of reactions of MXene-derived surface oxygen-containing groups with urea oxygen, which gradually yielded rather large particles of rutile. At the same time, there is a simultaneous alloying of MXene with nitrogen due to urea decomposition reactions with ammonia release (16) and (17), which was recorded during the prolonged treatment for 12 h and 18 h.

(16) $$\mathrm{CO}({\mathrm{NH}}_{2}{)}_{2}+({\mathrm{CH}}_{2}\mathrm{OH}{)}_{2}\overset{heating}{\to }C_{3}H_{4}O_{3}+2{\mathrm{NH}}_{3}$$

(17) $$\mathrm{CO}({\mathrm{NH}}_{2}{)}_{2}\overset{H_{2}O}{\to }\mathrm{HNCO}+{\mathrm{NH}}_{3}$$

For the sample prepared at the maximum duration of solvothermal synthesis, for 18 h, an increased sensitivity to low concentrations of ammonia was observed; at 20 °C, a response to 200 ppb of NH_3_ was equal to ca. 7.3% [114]. Since the material was highly sensitive to humidity, the authors proposed combining the sensitive element with a waterproof polytetrafluoroethylene membrane.

In order to manufacture sensitive humidity sensors capable of working in a wide concentration range, a method of growing potassium titanate nanofibers on the surface of MXene as a result of the interaction of accordion-like or delaminated Ti_3_C_2_T_x_ with a concentrated KOH solution was developed by Wu et al. [115]. Here, the adsorption of H_2_O molecules is promoted both by increasing the interlayer distance and by the formation of hydrophilic layered K_2_Ti_4_O_9_ nanowires, 10–50 nm thick, for which the ability to attract water molecules is assumed. It was shown that delamination of the original accordion-like MXene with its subsequent modification by K_2_Ti_4_O_9_ allows for an 8-fold increase in sensitivity when compared with the original MXene. The authors attribute this advancement in sensory properties to three aspects [115]:

(1) Enlarging the interlayer distance in Ti_3_C_2_T_x_ MXene caused by the intercalation of K^+^ cations and contributing to the intercalation of H_2_O molecules into it;
(2) The formation of a layered hydrophilic K_2_Ti_4_O_9_, which is thought to promote the adsorption and intercalation of H_2_O molecules;
(3) The filamentous and porous nanostructure of the material has an increased specific surface area, which provides more efficient contacts between the sensitive material and H_2_O molecules.

Thus, partial oxidation of both low-layer and accordion-like Ti_3_C_2_T_x_ MXenes allows us a significant increase in sensitivity, mainly to ammonia, moisture, VOCs, and NO_2_. The detection mechanism likely depends significantly on the degree of degradation of the original MXene and its transformation into highly dispersed and porous TiO_2_.

**SnO_2_**. Another oxide of interest, SnO_2_, is currently the most common receptor material used to design MOS sensors [116]. Therefore, quite a number of publications are devoted to the study of the possibilities of combining its positive properties with the advantages of 2D MXene nanomaterials.

For example, in a study published in [117], an electrostatic assembly of the Ti_3_C_2_T_x_/SnO_2_ nanocomposite was performed by mixing dispersions of SnO_2_ nanoparticles synthesized by the hydrothermal method with a few-layer Ti_3_C_2_T_x_ MXene obtained by the MILD process (Table 2). Analysis of the technique as well as XRD and SEM data indicate that SnO_2_ is predominant in the composition of the composite. The composite layers demonstrated a greater sensitivity compared with the individual components when detecting 0.5–100 ppm of NH_3_, as well as a significant decrease in response time.

Wang and colleagues [118] also studied the effect of modifying SnO_2_ microspheres with some amount of multilayer Ti_3_C_2_T_x_ MXene synthesized using concentrated hydrofluoric acid on the sensory properties. To obtain SnO_2_/Ti_3_C_2_T_x_ nanocomposites, 10–50 wt.% of accordion-like MXene particles were introduced into the reaction mixture for hydrothermal production of porous SnO_2_ microspheres. It was found that in the normal temperature range of the highest SnO_2_ sensitivity, 160–260 °C, the response to 10 ppm of ethanol for the composites was significantly higher than that for pure SnO_2_ and even more when compared with that of Ti_3_C_2_T_x_. The material containing 20 wt.% of Ti_3_C_2_T_x_ exhibited the highest response at the optimum temperature of 230 °C. It was found that the obtained SnO_2_ microspheres grafted onto accordion-like Ti_3_C_2_T_x_ particles were almost insensitive to inorganic analytes, NH_3_, NO_2_, but showed a relatively low response to 10 ppm H_2_S and high responses to VOCs, especially to ethanol, to equal 5% upon exposure to 10 ppm of this analyte. Increasing the background humidity from 40 rel.% to 90 rel.% reduces the response to 3.7%. The authors conclude that the main role in the adsorption of gaseous analytes over the nanocomposites is played by microspheres of semiconducting tin dioxide, while the introduced MXene plays the role of a sensitizer. In this work, the authors could clarify the background reasons for the positive effect of Ti_3_C_2_T_x_ addition on the gas-sensitive properties of SnO_2_ microspheres:

(1) The presence of a large number of functional groups (–O, –OH, and –F) on the Ti_3_C_2_T_x_ surface, which contribute to the nucleation and growth of SnO_2_ with a large number of active adsorption centers;
(2) Schottky barrier formation between the metal surface of MXene and semiconductor SnO_2_, which can lead to distortion of its band structure and reduce the thickness of the electron-depleted surface layer in contact with adsorbed molecules of ethanol;
(3) Acceleration of the mobility of charge carriers due to conductive Ti_3_C_2_T_x_ between SnO_2_ particles leads to improvements in kinetic characteristics such as response and recovery rates of the sensor.

**Figure 15:**
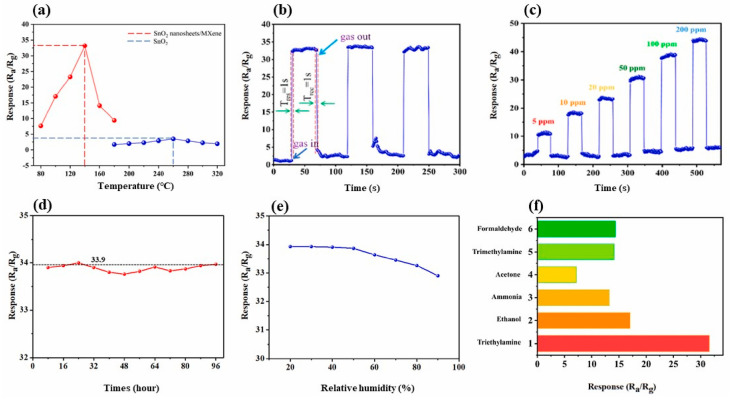
(**a**) Gas-sensing response of SNTM composites and pure SnO_2_ based sensor to 50 ppm of triethylamine (TEA) at diverse operating temperatures. (**b**) Reproducibility, response, and recovery characteristics of SNTM composites based on sensors upon exposure (3 cycles) to 50 ppm TEA gas at 140 °C. (**c**) The dynamic response and recovery curves of SNTM composites-based sensor to TEA with the increasing concentrations. (**d**) Stability of the sensor on successive exposure to 50 ppm TEA at 140 °C; (**e**) Sensor response of SNTM to 50 ppm TEA at various humidity conditions. (**f**) The selectivity of the SNTM composites-based sensor to different gases at 50 ppm TEA. Reprinted with permission from [119]. Copyright 2022: Elsevier B.V.

The hydrothermal method to synthesize SnO_2_/Ti_3_C_2_T_x_ (SNTM) nanocomposites was also employed in another work [119]. For this purpose, an aqueous solution of urea and cetriltrimethylammonium bromide was mixed with a dispersion containing tin tetrachloride monohydrate and ultrasound-delaminated Ti_3_C_2_T_x_ MXene, to be followed by hydrothermal treatment at 140 °C. Both the ratio of the initial reagents and the yellowish color of the output product indicate that the major phase in the synthesized nanocomposite is layered SnO_2_, which is oriented perpendicular to the plane of MXene. For the obtained hierarchical structure, the optimum operating temperature versus the detection of 50 ppm of triethylamine was found to be equal to 140 °C, which is significantly lower than that characteristic for pure SnO_2_ obtained by a similar technique, which is equal to 260 °C. Moreover, the response value, R_0_/R, for the SnO_2_/Ti_3_C_2_T_x_ nanocomposite was an order of magnitude higher than that for individual tin dioxide, being 33.9 when compared with 3.4 (Figure 15). 

It was found that the influence of humidity at RH < 60% for this material is practically absent. The authors justified the detection mechanism here by using the traditional MOS sensor notions of redox surface reactions between adsorbed gas molecules and ion-adsorbed oxygen species on the surface of SnO_2_ particles. The reason for the positive effect of the introduction of Ti_3_C_2_T_x_ MXene is believed to be the orienting role of its surface functional groups, –F, –O, and –OH, which set the growth direction of SnO_2_ nanosheets perpendicular to the Ti_3_C_2_T_x_ layers, which prevents their aggregation. A corresponding growth of the specific surface area leads to an increase in the number of active adsorption centers. The removal of F-groups from the surface of MXene under the hydrothermal process promotes the content of other functional groups, –O and –OH, due to the formation of hydrogen bonds that enhance the adsorption energy of the triethylamine molecule and the corresponding number of charge carriers. As the third cause of sensitization, the authors suggest Schottky barrier formation at the interface between SnO_2_ and MXene.

In order to design a highly sensitive NO_2_ sensor operable at RT, a hydrothermal synthesis method was used in a study published in [120] to synthesize SnO_2_/Ti_3_C_2_T_x_ nanocomposites. For this purpose, a water-alcohol solution containing multilayer Ti_3_C_2_T_x_ MXene, SnCl_4_-5H_2_O, polyvinylpyrrolidone, and hydrochloric acid was hydrothermally treated. The content of hydrochloric acid was varied from 4% to 11% in order to control the {221} and {110} SnO_2_ planes’ growth. During the treatment, the hydrolysis of SnCl_4_-5H_2_O and the appearance of SnO_2_ particles on the surface of accordion-like particles of MXene take place, where particle microstructure depends on the concentration of HCl. Thus, spindle-shaped particles with a predominance of {110} faces are grown at a minimum amount of acid.

**Figure 16:**
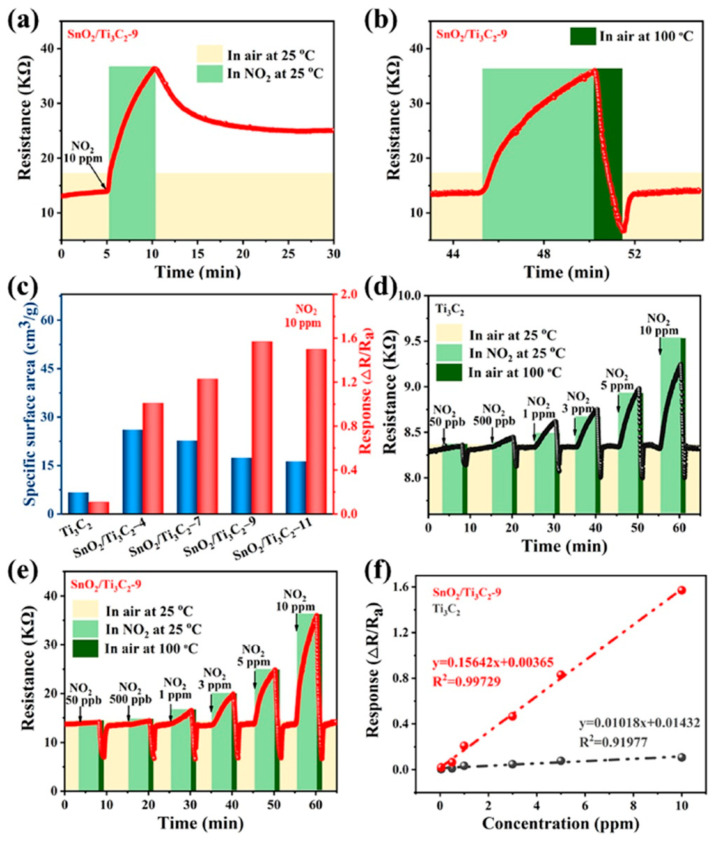
Response/recovery curves of SnO_2_/Ti_3_C_2_-9%HCL composite at RT (**a**) before and (**b**) after the pulse-heating treatment; (**c**) specific surface area and responses of pristine Ti_3_C_2_ and SnO_2_/Ti_3_C_2_ composites to 10 ppm NO_2_; (**d**,**e**) response/recovery curves; and (**f**) linear fitting curves of pristine Ti_3_C_2_ and SnO_2_/Ti_3_C_2_-9 composite to 0.5–10 ppm NO_2_. Reprinted with permission from [120]. Copyright 2022: Elsevier B.V.

Then, there occurs an octahedral formation of dodecahedral crystals with four {110} faces at 7% of HCl and eight {221} faces at 9% of HCl. For the samples with a combination of faces {110} and {221}, the sensing properties were observed at an operating temperature of 25 °C, followed by a pulse heating up to 100 °C for desorption. Under these conditions, the maximum response was recorded toward 10 ppm of NO_2_, equal to ca. 1.6%, which is an order of magnitude greater than the one observed in the case of just Ti_3_C_2_T_x_, equal to ca. 0.1%, as shown in Figure 16. At the same time, other test gases—10 ppm of CO, NH_3_, SO_2_, and H_2_S—yielded a lower sensor signal when compared with nitrogen dioxide. 

**Figure 17:**
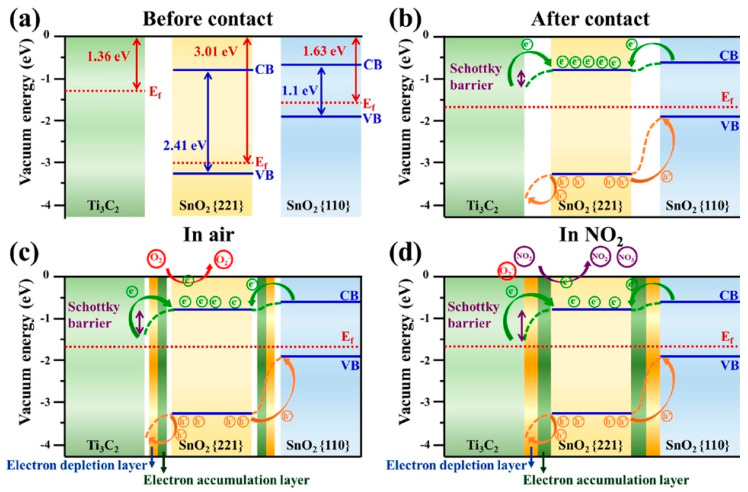
(**a**) Band structures of Ti_3_C_2_, SnO_2_ {221} and {110} facets before contact, and variations of Schottky barrier of SnO_2_/Ti_3_C_2_ composite under different conditions: (**b**) after contact, (**c**) in air, and (**d**) in NO_2_. Reprinted with permission from [120]. Copyright 2022: Elsevier B.V.

When analyzing the detection mechanism, the authors stated that for {221} faces, all the Sn cations are unsaturated and are characterized by a greater number of broken chemical bonds as compared with {110} faces. This may be the reason for the higher sensitivity of samples synthesized at higher acidity (9% HCl). In addition, a heterojunction can form on the surface of SnO_2_ particles (Figure 17) due to the difference in the band structure of these facets, contributing to the efficient electron transfer between the SnO_2_/Ti_3_C_2_T_x_ composite and the adsorbed NO_2_ molecules. The difference in Fermi levels leads to an electron overflow from the Ti_3_C_2_T_x_ and {110} SnO_2_ facets to the {221} SnO_2_ facets, resulting in curved energy bands and electron accumulation at the surface. These electrons can be localized at the surface while being captured by ion-adsorbed species of oxygen. When NO_2_ adsorbs, it can localize free electrons in accordance with Reactions (14) and (15), which additionally cause a distortion of energy zones by enhancing both the Schottky barrier and resistance of the SnO_2_/Ti_3_C_2_T_x_ composite. That is, the formation of Schottky heterojunctions and surface heterojunctions promotes accelerating a charge transfer, thereby improving the sensitivity of the SnO_2_/Ti_3_C_2_T_x_ composite to NO_2_ at low concentrations [120].

Yao et al. [121] found that the hydrothermal treatment of multilayer Ti_3_C_2_T_x_ MXene in an aqueous solution containing SnCl_2_-2H_2_O, urea, and hydrochloric acid resulted in the formation of a Ti_3_C_2_T_x_/SnO nanocomposite. XRD analysis of the material indicates a partial oxidation of SnO to SnO_2_, but SEM and TEM images did not allow one to find the particles whose interplanar spacing corresponded to the SnO_2_ phase among the disordered array of SnO nanosheets grown on undelaminated MXene. Sn 3D XPS spectra verify that there is a fairly large component from Sn(II), in addition to the Sn(IV) state, which was absent for individual SnO_x_ synthesized under the given conditions. This allows the authors to conclude that the presence of Ti_3_C_2_T_x_ inhibits the oxidation of SnO to SnO_2_, which occurs very easily in air. The study of exposing the material at RT to 200 ppm of various gases demonstrated its high *p*-type sensitivity to ammonia with an R_0_/R response equal to ca. 7.8. For the other studied VOCs, except for ethanol, the resistance has decreased under the *n*-type response but is still rather low, not exceeding 1.6. It indicates a decent selectivity toward NH_3_ vapors. Nevertheless, an aging of the material for more than 7 days was observed, which the authors attributed to SnO oxidation as well as a high sensitivity to background humidity.

The authors of [121] attributed the superior gas sensitivity of the Ti_3_C_2_T_x_/SnO composite material over that of the individual components to a number of factors. First, the –O, –OH, and –F functional groups existing on the surface of MXene layers promote the growth of ultrathin SnO sheets, which is promising for accelerating electron diffusion. The higher specific surface area contributes to more available adsorption centers for oxygen and analyte molecules from the surrounding environment. Accounting for the observed chemiresistive behavior of Ti_3_C_2_T_x_/SnO composites, the authors concluded that here SnO exhibits *n*-type semiconductor properties while Ti_3_C_2_T_x_ with metallic conductivity plays the role of a conductive channel, which reduces the resistance compared with pure SnO. In addition, MXene enforces the electron transfer rate and consequently improves the kinetic characteristics of the sensor. The p-n junctions that appeared between Ti_3_C_2_T_x_ and SnO enabled higher concentrations of charge carriers and the suppression of their recombination.

**Figure 18:**
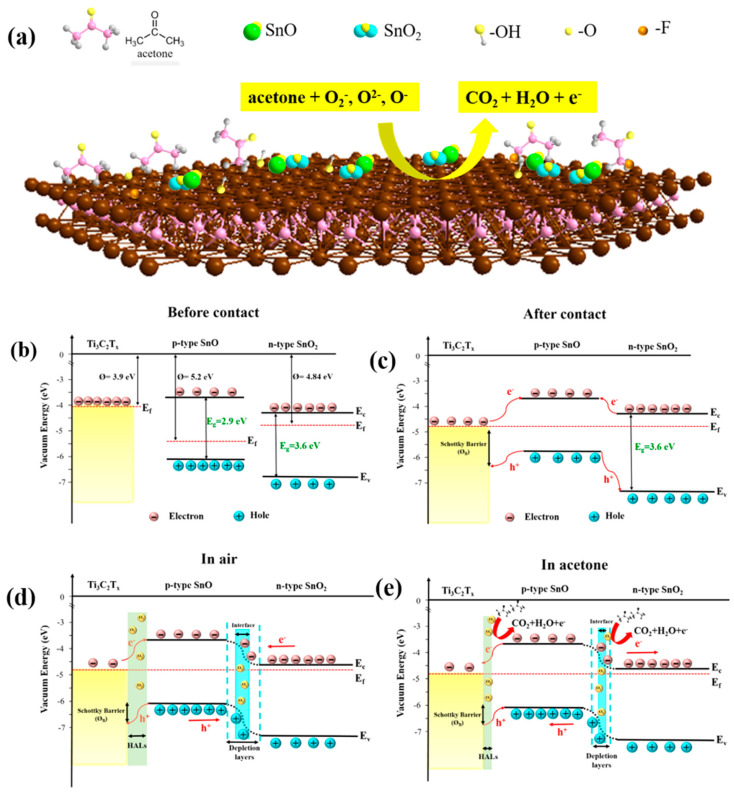
(**a**–**e**) Schematic diagram of the band structure of p-n hetero-junction and the contact of the SnO-SnO_2_/Ti_3_C_2_T_x_ sensor. Reprinted with permission from [112]. Copyright 2021: Elsevier B.V.

The synthesis of the nanocomposite, similar to that reported in [121], was performed by Wang et al. [112]. However, these authors emphasized observing two oxide phases, SnO and SnO_2_, as a result of Sn^2+^ oxidation by residual oxygen in the autoclave. The resulting SnO-SnO_2_/Ti_3_C_2_T_x_ nanocomposite displayed high sensitivity to acetone in a wide range of concentrations, 10–100 ppm. The R/R_0_ response to 100 ppm of acetone was ca. 12.1 at RT, which is almost eleven and four times higher than one of the original Ti_3_C_2_T_x_ MXene and SnO-SnO_2_ oxide composites, respectively. At the same time, the resistance gradually enhanced under the gas exposures for the SnO-SnO_2_ and SnO-SnO_2_/Ti_3_C_2_T_x_ composites, while an unusual drop in resistance was observed in the original multilayer MXene obtained by etching the MAX-phase with concentrated hydrofluoric acid. The synthesized composite material also showed good responses to toluene, ethanol, and methanol, the value of which, however, is significantly lower than that to acetone and is in the range of 2.9–4.3. The long-term stability of the SnO-SnO_2_/Ti_3_C_2_T_x_-based sensor was evidenced by recording the response value to acetone for 35 days. The authors considered the acetone detection by the obtained nanocomposite via the existence of p-n-heterojunctions in the SnO-SnO_2_ oxide particles formed on the MXene surface, which caused the increased concentration of charge carriers and the suppression of charge carrier recombination. This system seems to have a layered structure: SnO primarily appears on the surface of Ti_3_C_2_T_x_ from the solution, which is converted to SnO_2_ at the outer surface of the SnO/Ti_3_C_2_T_x_ composite via reacting with oxygen to be a *n*-type semiconductor. Therefore, a charge transfer occurs from SnO_2_ and Ti_3_C_2_T_x_ with metallic conductivity to the p-semiconducting SnO, which leads to a distortion of the band structure (Figure 18).

When oxygen molecules are adsorbed from air to induce primarily O_2_^−^ ions on the surface, a depleted layer is generated at the p-n junction boundary while a layer of hole accumulation (HALs) is formed at the SnO/Ti_3_C_2_T_x_ interface. This increases the mobility of charge carriers and reduces the Schottky barrier height, i.e., the resistance at RT has a relatively low value. Acetone reacts with ion-adsorbed oxygen following Reaction (18), releasing electrons that recombine with holes and cause a higher resistance.

CH_3_COCH_3_ + 4O_2_^−^ → 3CO_2_ + 3H_2_O + 4ē (18)

The authors of the study [112] highlighted the main contribution of Ti_3_C_2_T_x_ MXene to the yielding of the composite morphology in the form of a layered framework with a high specific surface area and, consequently, with a large number of active adsorption centers. In addition, due to the high electrical conductivity of Ti_3_C_2_T_x_, an electron transfer under the gas detection is accelerated, which leads to a reduction in the sensor’s response and recovery time.

To summarize, it should be emphasized that the modification of Ti_3_C_2_T_x_ MXene with *n*-type SnO_2_ semiconductors has not been considered in this section. Rather, the talk is about SnO_2_ sensitization by 2D MXene with metallic conductivity. In this regard, it was possible to reduce in some cases the operating temperature of the SnO_2_–based sensor down to RTRT. In addition, the increased sensitivity of the resulting nanocomposites is noted, primarily due to the appearance of Schottky junctions and the significantly increased specific surface area because the MXene plays a role as a framework template for the growth of oriented SnO_2_ nanoparticles. Nevertheless, a number of questions arise following the reported data. For instance, the synthesis of a number of SnO_2_/Ti_3_C_2_T_x_ nanocomposites is carried out in the framework of a hydrothermal approach, which promotes a significant degradation of MXene structures with growing TiO_2_ nanoclusters, whose appearance is frequently documented by XRD via corresponding reflections. Therefore, the reasoning about the detection mechanisms, at least for a number of experimental results, should most likely be complicated by adding the TiO_2_ phase, an *n*-type semiconductor, into consideration.

**ZnO**. Zinc oxide is the second most common semiconductor oxide used for the detection of gaseous analytes in chemiresistive sensors. Recently, the number of publications devoted to employing ZnO in gas sensors has almost equaled that on SnO_2_ [116]. Therefore, many scientific groups attempted to combine MXenes and ZnO [122,123,124,125,126].

**Figure 19:**
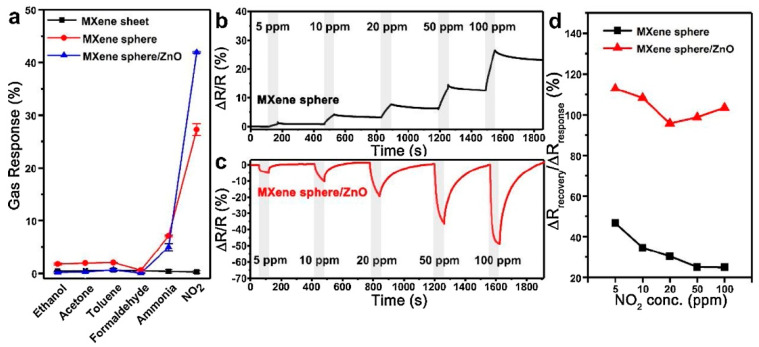
The selectivity, concentration sensitivity, and recovery characteristics of flexible gas sensors based on Ti_3_C_2_T_x_ MXene sheet, 3D crumpled MXene sphere, and 3D crumpled MXene sphere/ZnO. (**a**) The gas response of sensors based on MXene sheets, MXene spheres, and MXene spheres/ZnO to various target gases at a concentration of 100 ppm. (**b**,**c**) Dynamic response/recovery curve of sensors based on MXene spheres and MXene spheres/ZnO to NO_2_ at concentrations of 5, 10, 20, 50, and 100 ppm at 70% RH. (**d**) The recovery rate (ΔR_recovery_/ΔR_response_) of sensors based on MXene spheres and MXene spheres/ZnO for different concentrations of NO_2_. Reprinted with permission from [122]. Copyright 2021: Elsevier B.V.

In order to create a flexible NO_2_ sensor allowing a chemiresistive response at RT, the Ti_3_C_2_T_x_/ZnO nanocomposite was considered in the study by Yang et al. [122]. In addition to the polyvinylpyrrolidone, zinc acetate was also introduced into an initial solution for ultrasonic pyrolysis in the process of obtaining the crumpled MXene spheres. Thermal treatment of the aerogel in a N_2_ flow at 800 °C resulted in the formation of a nanocomposite with the morphology of a 3D crumpled MXene sphere, whose surface, especially on the protruding ribs, was covered with ZnO nanoparticles of ca. 10 nm diameter that were evenly distributed. Among a large number of analyte gases tested on the Ti_3_C_2_T_x_/ZnO sample, there was a sharp increase in the response value versus NO_2_ compared with pristine Ti_3_C_2_T_x_; the response to 100 ppm of NO_2_ at RT and humid air was ca. 41.9% in the case of the Ti_3_C_2_T_x_/ZnO sample compared with ca. 27.3% in the case of Ti_3_C_2_T_x_, respectively. A significant improvement in the kinetic characteristics of the obtained nanocomposite was also noted, including a relatively fast recovery of resistance to the baseline, as shown in Figure 19. The authors showed that the sensor signal is stable under repeated bending, including an angle of up to 120°, which is important for portable electronics devices on flexible substrates. It was shown that the signal magnitude decreased by 31% even after performing 1000 bends at 90°, together with some loss of film conductivity. There was a tendency for the response value toward 100 ppm of NO_2_ to advance from ca. 28.9% to ca. 51.4% when the humidity in the background air increased from 20 rel.% to 90 rel.%.

An unusual reduction of resistance was observed [122] in Ti_3_C_2_T_x_/ZnO upon NO_2_ exposure (in contrast to ammonia, which led to a resistance enhancement). This behavior differed from that of crumpled, pristine Ti_3_C_2_T_x_ spheres, which exhibited an increase in resistance versus both analytes. The authors considered that the relative content of oxidized titanium, TiO_2_, as verified by XPS, is higher in the Ti_3_C_2_T_x_/ZnO nanocomposite. As a result, the metal conductivity of the MXene is converted to a *p*-type semiconducting one. Thus, these composite structures contain heterogeneous p-n junctions with ZnO nanoparticles synthesized on the Ti_3_C_2_T_x_ surface, yielding an electron-depleted layer at this interface. The adsorption of NO_2_ molecules leads to the expansion of this layer, which improves charge transfer efficiency. The adsorbed water molecules, as stated by the authors, also lead to the effect of n-alloying and additionally promote the sensor response to NO_2_, with which they are able to form hydrogen bonds.

**Figure 20:**
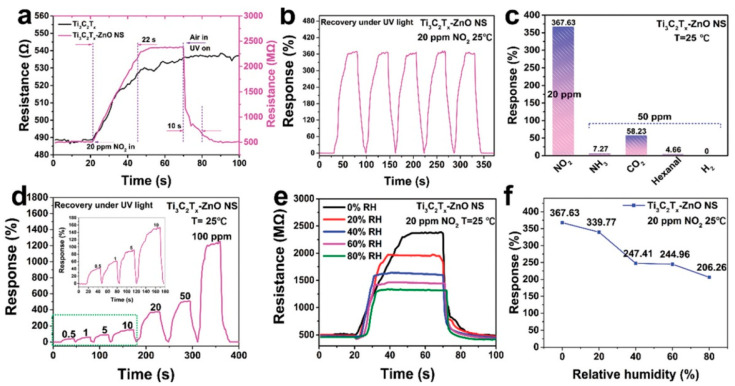
(**a**) Dynamic response and recovery curves under UV irradiation for Ti_3_C_2_T_x_ MXene and Ti_3_C_2_T_x_–ZnO NS sensors to 20 ppm NO_2_ at RT. (**b**) Repeatability tests of Ti_3_C_2_T_x_–ZnO NS sensor to 20 ppm NO_2_ at RT. (**c**) Selectivity of Ti_3_C_2_T_x_–ZnO NS sensor to 20 ppm NO_2_ and various target gases with a concentration of 50 ppm at RT. (**d**) Dynamic response and recovery curves of Ti_3_C_2_T_x_–ZnO NS sensor under UV illumination to 0.5–100 ppm NO_2_ at RT. (**e**) Dynamic response and recovery tests for the Ti_3_C_2_T_x_–ZnO NS sensor in the RH range of 0–80% RH. (**f**) The response values of the Ti_3_C_2_T_x_–ZnO NS sensor at different humidity conditions ranging from 0% to 80% RH. Reprinted with permission from [123]. Copyright 2022: The Royal Society of Chemistry.

The work by Fan et al. [123] is also devoted to obtaining highly sensitive nitrogen dioxide sensors using the Ti_3_C_2_T_x_/ZnO composite. To obtain the receptor material, a Ti_3_C_2_T_x_ dispersion synthesized by etching aluminum from the MAX-phase structure was mixed with hydrothermally synthesized porous layered ZnO nano-powder calcined at 450 °C. After ultrasonic treatment of the combined suspension, a receptor layer was applied onto an electrode structure to serve as a sensor. The hybrid material obtained in this manner showed high sensitivity toward NO_2_, a response to 20 ppm of ca. 367.6%, and some selectivity (Figure 20). The authors believe that the porous morphology of the layers and the oxygen vacancies in ZnO contribute to the favorable adsorption of gaseous NO_2_, as does the presence of surface functional groups (–F, –OH, –O) on the surface of the Ti_3_C_2_T_x_ MXene layers. Furthermore, the authors found the UV irradiation useful to substantially reduce the sensor recovery time, down to 22 s.

**Figure 21:**
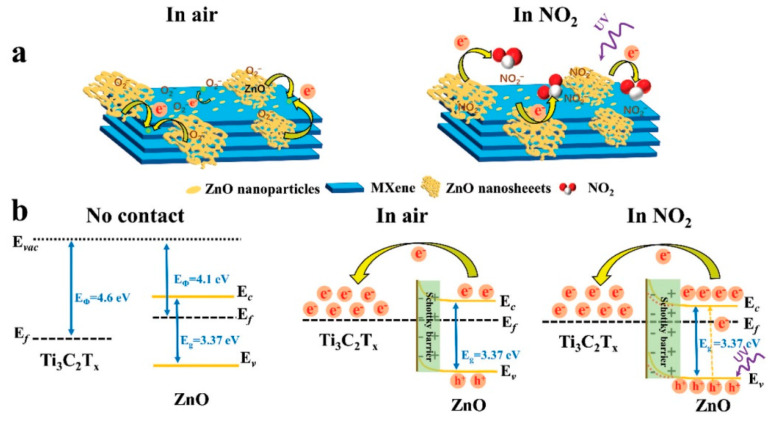
(**a**) Schematic diagram of the possible gas-sensing mechanism of Ti_3_C_2_T_x_/ZnO NS. (**b**) Energy band structure diagram of Ti_3_C_2_T_x_/ZnO NS Schottky barriers in air and NO_2_. Reprinted with permission from [123]. Copyright 2022: The Royal Society of Chemistry.

This effect is attributed to the photogeneration of charge carriers in ZnO under UV (Figure 21), which release chemically adsorbed NO_2_ molecules according to Reactions (19)–(21).

NO_2(gas)_ + ē → NO_2_^−^_(ads)_ (19)

Hν → h^+^(hν) + ē(hν) (20)

NO_2_^−^_(ads)_ + h^+^(hν) → NO_2(gas)_ (21)

DFT calculations indicate that the main adsorption centers belong to ZnO nanosheets, while Ti_3_C_2_T_x_ acts as a conductive channel to accelerate charge transfer [123].

**Figure 22:**
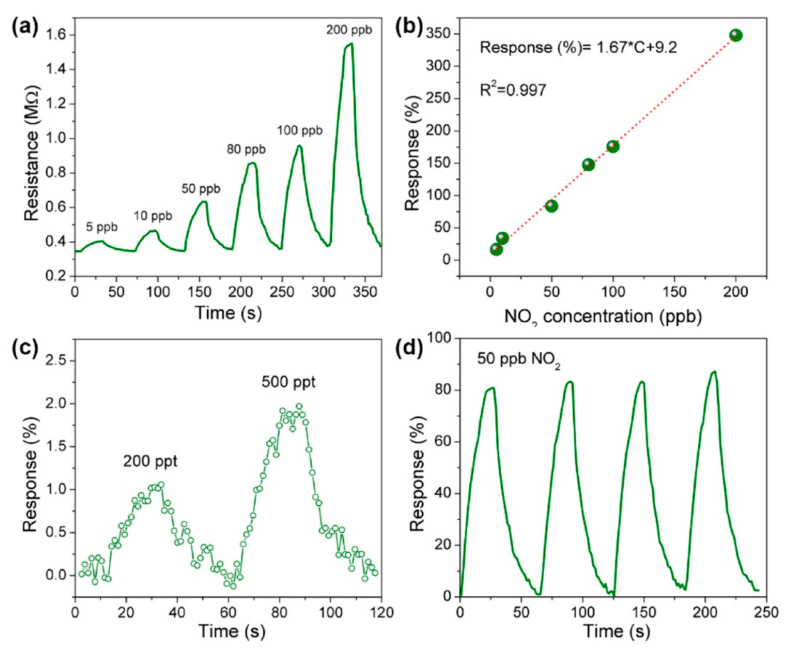
Dynamic response curves (**a**) and responses with linear line fitting (**b**) of the Ti_3_C_2_T_x_/ZnO-NR-based sensor upon the exposure to NO_2_ (5–200 ppb) under UV illumination; (**c**) dynamic response curves of the Ti_3_C_2_T_x_/ZnO-NR-based sensor upon the exposure to NO_2_ (200 and 500 ppt) under UV illumination; (**d**) reproducibility and reversibility of the Ti_3_C_2_T_x_/ZnO-NR-based sensor upon the exposure to NO_2_ (50 ppb) under UV illumination (for 4 successive cycles). Reprinted with permission from [124]. Copyright 2022: Elsevier B.V.

A more complex multistep synthesis was employed to obtain a mesoporous Ti_3_C_2_T_x_/ZnO composite as part of an efficient NO_2_ optoelectronic sensor [124], where ZnO nanorods were grown on the surface of MXene from seed ZnO nanoparticles. The oxide seed nanoparticles were deposited on the surface of delaminated Ti_3_C_2_T_x_ from a methanol–zinc acetate solution by interaction with NaOH. The specific surface area of the material was higher than 145 m^2^/g. The resulting composite powder was further dispersed in an aqueous NaOH solution together with the previously synthesized ε-Zn(OH)_2_ and held at 80 °C for the aging and growth of ZnO nanorods. The EDS analysis data revealed that the ZnO content was ca. 63.5 wt.%. The comparison of responses of the composite to 50 ppb of NO_2_ in the dark and under UV irradiation showed that UV forces the sensor signal to grow from ca. 6.5% up to ca. 81% with low response, 17 s, and recovery, 24 s, times. Such a high sensitivity gives an option to measure ultra-low NO_2_ concentrations down to a detection limit of 0.2 ppm (Figure 22). The response to NO_2_ was quite selective when compared with other gaseous pollutants under test such as SO_2_, NH_3_, H_2_S, and CO. In particular, the response to 50 ppb of NH_3_, second in value to that for NO_2_, was almost four times lower. At a higher relative humidity of RH = 80%, the response decreased to approx. 50%, which the authors attribute to the competing adsorption of water molecules.

The effect of the component ratios in the ZnO/Ti_3_C_2_T_x_ nanocomposite on the gas-sensing properties was investigated by Zhu et al. [125]. To produce the receptor materials in two types, containing multilayer and few-layer Ti_3_C_2_T_x_, the dispersion of the corresponding MXene in zinc acetate solution and CTAB (cetyltrimethylammonium bromide) was hydrothermally treated at a relatively low temperature of 120 °C. The content of Ti_3_C_2_T_x_ in the different samples varied from 1 wt.% to 3 wt.%.

It was found that the composites containing delaminated Ti_3_C_2_T_x_ MXene exhibit greater sensitivity to acetone, with an R_0_/R response to a 100 ppm concentration equal to 14.1 at 320 °C of operating temperature for the 2 wt.% Ti_3_C_2_T_x_ when compared with samples based on multilayer Ti_3_C_2_T_x_.

**Figure 23:**
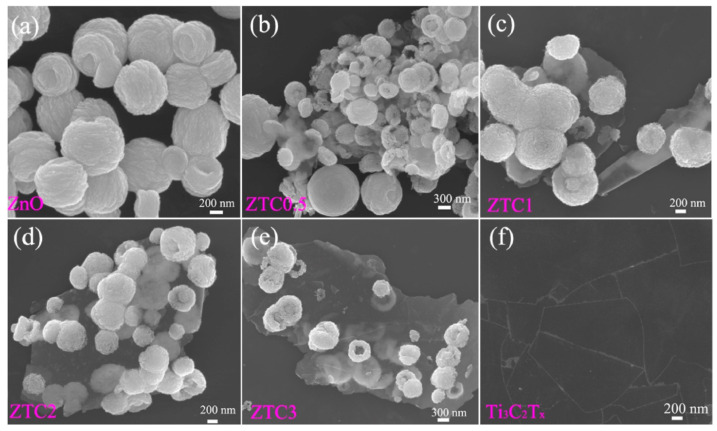
Morphology of the as-synthesized ZnO (**a**), ZnO/Ti_3_C_2_T_x_ nanocomposites (**b**–**e**), and pure Ti_3_C_2_T_x_ (**f**). Reprinted with permission from [126]. Copyright 2022: Elsevier B.V.

The study by Liu et al. [126] was also dedicated to the effect of doping zinc oxide with small amounts of delaminated Ti_3_C_2_T_x_ MXene on its sensitivity to nitrogen dioxide. To obtain the ZnO/Ti_3_C_2_T_x_ composite in the form of spherical porous particles, zinc chloride, sodium acetate, and sodium citrate were added to the dispersion of MXene in ethylene glycol, followed by solvothermal synthesis at 200 °C. For the composite material containing 2% of Ti_3_C_2_T_x_ with the maximum specific surface area, the dependence of the response value toward 10 ppm of NO_2_ on the operating temperature in the range of 150–180 °C was studied. It was found that the response at 160 °C is 1.4–2.9 times higher as compared with that at other temperatures. Measurement of response to various gases (CO, H_2_S, NO_2_, C_2_H_5_OH, NH_3_, CH_2_OH) at 10 ppm concentration showed that the ZnO/Ti_3_C_2_T_x_ material obtained by the method is more selective to NO_2_. The authors explain the advancement of the sensory characteristics of ZnO in terms of reducing the operating temperature from 220 °C to 160 °C and increasing the response when it is modified with some amount of few-layer Ti_3_C_2_T_x_ due to the higher specific surface area and appearance of mesoporous structure, which facilitates more active sorption centers as compared with pristine ZnO. Another factor is the high electrical conductivity of metallic Ti_3_C_2_T_x_ layers, which enables fast transport of charge carriers. Because the porous ZnO spheres are anchored on both sides of the Ti_3_C_2_T_x_ layers (Figure 23), they can act as channels for free carriers and effectively reduce the potential barriers at the grain boundary between the ZnO spheres.

**Figure 24:**
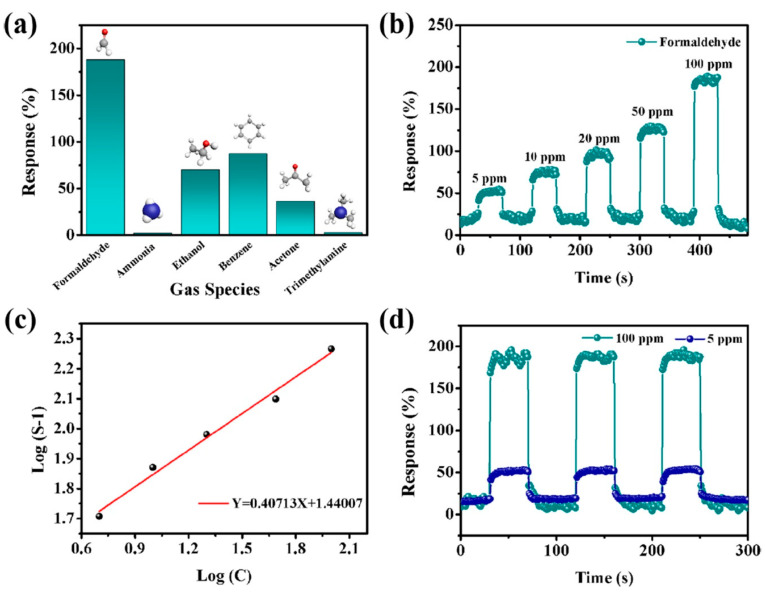
(**a**) Selective curve of the ZnSnO_3_/Ti_3_C_2_T_x_ composite to 100 ppm of various gases at RT. (**b**) Dynamic response curves for 5–100 ppm formaldehyde, and (**c**) corresponding function fitting plots of sensors at RT. (**d**) cyclic response of ZnSnO_3_/Ti_3_C_2_T_x_ composites to 5 and 100 ppm formaldehyde at RT. Reprinted with permission from [127]. Copyright 2022: Elsevier B.V.

In order to modify nanoporous cubic tin-zinc oxide (ZnSnO_3_) aggregates with delaminated Ti_3_C_2_T_x_ sheets, Sima and colleagues [127] performed a hydrothermal treatment of a pre-synthesized suspension of MXene and ZnSnO_3_, with its surface modified with the cationic surfactant CTAB to electrostatically assemble the individual components. The measured CVC curves demonstrated that the ZnSnO_3_/Ti_3_C_2_T_x_ hybrid material has a higher electrical conductivity compared with the initial ZnSnO_3_. Furthermore, the high sensitivity of the obtained composite to 100 ppm of formaldehyde with a response equal to 194.7% at RT and short response and recovery times of 6 s and 5 s, respectively, was found out. Measuring the responses to 100 ppm of other toxic and practically significant VOCs (ammonia, alcohol, benzene, acetone, and trimethylamine) revealed that the sensitivity to formaldehyde was at least 2.1 times higher than that to each of the above test analytes (Figure 24). The authors discussed this as advancing to the higher enthalpy of formaldehyde adsorption. The detecting mechanism of ZnSnO_3_-based semiconductor materials is based on the well-known redox interaction between formaldehyde molecules and chemisorbed oxygen (Reactions (22) and (23)), resulting in a free electron release and thinning of the electron-depleted semiconductor layer [127].

HCHO_(gas)_ → HCHO_(ads)_ (22)

HCHO_(ads)_ + O_2_^−^_(ads)_ → CO_2_+H_2_O+ē (23)

The authors see the contribution of impurities in delaminated MXene to improving the sensory properties of the composite material via:

(1) Preventing the aggregation of ZnSnO_3_ cubes with Ti_3_C_2_T_x_ sheets that advance the specific surface area of the material;
(2) Promoting the adsorption of organic molecules due to the presence of polar functional groups (–F, –OH, –O) on the surface of Ti_3_C_2_T_x_;
(3) Increasing the free carrier mobility due to the enhanced electrical conductivity of Ti_3_C_2_T_x_;
(4) Appearing as a heterojunction at the interface between ZnSnO_3_ and Ti_3_C_2_T_x_.

**WO_3_**. Another oxide, tungsten(VI), draws significant attention as an *n*-type semiconductor characterized by high sensitivity to ammonia. In order to achieve a response to NH_3_ at RT, Guo et al. [128] studied the potential of Ti_3_C_2_T_x_-WO_3_ composites by varying the WO_3_ content from 17 wt.% to 67 wt.%. The authors mixed ultrasonically treated Ti_3_C_2_T_x_ dispersions, obtained with HF, and hydrothermally synthesized WO_3_ to obtain the composite samples. Using the Ti_3_C_2_T_x_-50 wt.% WO_3_ sample as an example, XPS spectroscopy data revealed that this method of nanocomposite fabrication does enhance the Ti–X bond due to the oxygen atoms transferring from the WO_3_ nanoparticles to the MXene surface. The research on the Ti_3_C_2_T_x_/WO_3_ nanocomposites exposed to 1 ppm NH_3_ at RT indicated that an optimal phase ratio exists, which refers to the Ti_3_C_2_T_x_-50 wt.%WO_3_ composition. In this case, the observed response was ca. 22.3%, compared with the ca. 1.5% characteristic for pristine Ti_3_C_2_T_x_ [128]. At the same time, all samples except pure WO_3_ demonstrated a *p*-type response related to enhancing resistance upon analyte introduction, indicating that the chemiresistive response of the nanocomposites is determined by the Ti_3_C_2_T_x_ MXene. The reproducibility study on the example of four detection cycles for samples of Ti_3_C_2_T_x_-50 wt.% WO_3_ and Ti_3_C_2_T_x_ confirmed that the composite-based chemiresistor has better repeatability while the SNR matches that of a pure MXene with its metallic conductivity. The kinetic properties of the nanocomposite, alternatively, improve insignificantly compared with Ti_3_C_2_T_x_. The responses of the nanocomposite to other analytes (H_2_S, NO, CH_3_CH_2_OH, CH_3_CHO, and CH_3_COCH_3_) were ca. 2.4–17.3 times lower than those to NH_3_. The humidity suppressed the signal to NH_3_ and deteriorated the sensor baseline, which the authors attributed to the antagonism between H_2_O and NH_3_ molecules for adsorption centers [128]. The suggested mechanism of the *p*-type response to ammonia primarily relates to reducing carrier transport due to the adsorption of the gas molecules as surface defects. The increased sensitivity for Ti_3_C_2_T_x_-WO_3_ nanocomposites, similar to that of other *n*-type metal semiconducting oxides, is explained by the higher porosity and number of active centers provided by WO_3_ as well as by the heterojunction existing at the boundary between the metal Ti_3_C_2_T_x_ and the *n*-type semiconductor WO_3_.

**Figure 25:**
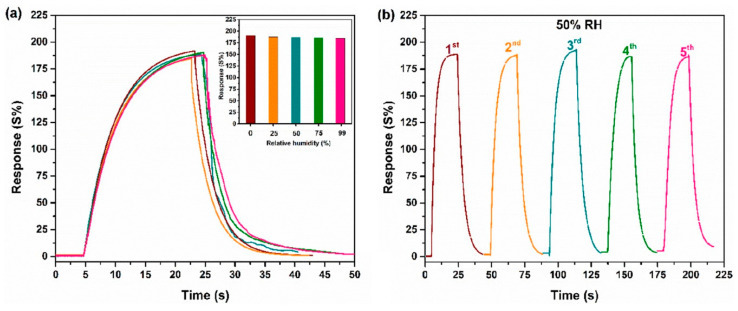
(**a**) Sensor response of sodium ascorbate-treated Ti_3_C_2_T_x_/WO_3_ nanocomposite at different humidity levels (RH= 0–99%) and (**b**) its signal reproducibility at 50% relative humidity versus 200 ppb NO_2_ at RT. Reprinted with permission from [129]. Copyright 2022: American Chemical Society.

Still, it is worth noting that the negative effect of humidity is observed in the study of [128] in all the sensors based on the pristine metal oxide, MXene, and Ti_3_C_2_T_x_/WO_3_ nanocomposites. In contrast, the work by Gasso et al. [129] dealing with the design of a NO_2_ sensor showed preserving the response value over a wide range of humidity in the background air (0–99 rel.%). In this work, the hydrothermal synthesis of WO_3_ nanorods in the presence of dispersion of Ti_3_C_2_T_x_, to be synthesized with concentrated HF, was performed to obtain a Ti_3_C_2_T_x_/WO_3_ nanocomposite. It was demonstrated that MXene plays in this case the role of an anchoring platform for WO_3_ nanorods, which limits the aggregation of the latter and increases the specific surface area [129]. The chemosensory properties of the nanocomposite tested at RT in a dry atmosphere toward a number of gases of oxidizing nature (NO_2_, I_2_, Br_2_, Cl_2_) and reducing nature (ammonia, acetone, ethanol, isopropanol) showed that it has the highest sensitivity to NO_2_ at a 30–1000 ppb concentration range. However, it is noted that the response to this analyte, at 0.2 ppm, goes from ca. 197% to ca. 145% while the humidity increases from 0 rel.% to 99 rel.%. Aiming to improve the stability over time and reduce the sensitivity of the material to humidity, the MXene dispersion was pre-treated with sodium L-ascorbate under the Ti_3_C_2_T_x_/WO_3_ nanocomposite synthesis [129], because there is evidence in the literature [126,130] that this approach is effective due to the screening of titanium atoms in Ti_3_C_2_T_x_ sheets from oxygen and water attack by C_6_H_7_O_6_ anions. These modified Ti_3_C_2_T_x_/WO_3_ composites exhibited a response to 200 ppb of NO_2_, equal to ca. 184% of the total range of RH = 0–99 (Figure 25).

Furthermore, Gasso’s group [131] designed a sensor for even lower NO_2_ concentrations, down to 15 ppb of concentration, considering the WO_3_/Ti_3_C_2_T_x_ nanocomposite synthesized by a stepwise hydrothermal method. In particular, the mixture of thiourea and nitric acid with Na_2_WO_4_ solution containing 10–40 wt.% of Ti_3_C_2_T_x_, up to pH = 3, was stirred for 1.5 h prior to hydrothermal treating. The gas-sensing properties of the sensor placed over a flexible polyamide substrate were studied only for the WO_3_-20 wt.% Ti_3_C_2_T_x_ sample. This composition exhibited high sensitivity and selectivity versus NO_2_, as it was documented in the previous work on WO_3_/Ti_3_C_2_T_x_ nanocomposites [129] obtained by another technique. The response to 200 ppb of NO_2_ at RT was found to be 78%, while for the same concentration of ammonia, it was 20%, SO_2_—13%, ethanol—11%, and for other gases under test, it did not exceed 10% [131].

At the same time, the 20 wt% Ti_3_C_2_T_x_-decorated tungsten oxide showed a reduced sensor response and recovery time compared with both pristine Ti_3_C_2_T_x_ MXene and pure WO_3_. The WO_3_/Ti_3_C_2_T_x_ material maintained sensor properties for 30 days. However, when the humidity in the background air varied from 0 rel.% to 99 rel.%, the response diminished from 78% to 56%, accompanying a rise in the sample resistance both in humid air and upon analyte injection [131].

A solvothermal growth of non-stoichiometric tungsten oxide (W_18_O_49_) nanorods on the surface of Ti_3_C_2_T_x_ MXene sheets was reported by Sun and colleagues [132] to fabricate a sensor for low concentrations of acetone. The choice of W_18_O_49_ as the major phase of the nanocomposite was due to the presence of a large number of oxygen vacancies, which can serve as additional active adsorption centers for the target gases. To synthesize the W_18_O_49_/(1–2.5 wt.%)Ti_3_C_2_T_x_ composite material in an acetylacetone-modified tungsten ethoxide solution, multilayer MXene powder was dispersed by ultrasound prior to placing it in an autoclave for solvothermal processing. The gas-sensing properties of the material were analyzed using VOCs and ammonia, at 20 ppm concentration as an example, at 200–400 °C temperatures. The measurements ensured an improved selectivity for acetone detection with W_18_O_49_/Ti_3_C_2_T_x_ structures characterized by a resistance reduction, or *n*-type response, which is characteristic for n-semiconducting oxides, including WO_x≤3_. It was shown that the operating temperature, optimal in terms of the response value and the response/recovery rates, is 300–350 °C, again similar to those observed in oxides. Moreover, the material decorated with 2 wt.% Ti_3_C_2_T_x_ stands out with improved chemiresistive properties: the response, R_0_/R, to 200 ppm of acetone at 300 °C was ca. 12.1, while in the case of the pristine W_18_O_49_ it was ca. 3.8. The reason for this phenomenon, according to the authors, is (i) advancing the specific surface area when W_18_O_49_ is placed on the Ti_3_C_2_T_x_ sheets surface, (ii) removing the fluoride surface groups in the MXene under the solvothermal synthesis and their replacement with OH– and O–groups, as well as (iii) an inter-phase interaction between the components having semiconductor and metallic conductivity characteristics, or the appearance of heterojunctions sensitive to environment.

**Figure 26:**
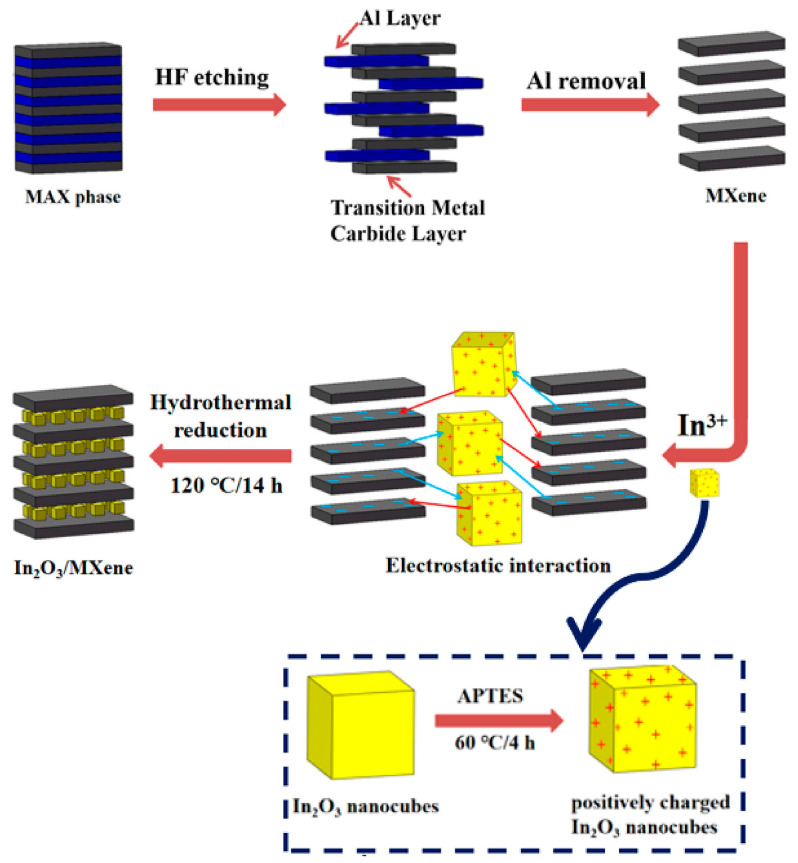
Scheme of In_2_O_3_/Ti_3_C_2_T_x_ composites formation. Reprinted with permission from [133]. Copyright 2021: Elsevier B.V.

**In_2_O_3__._** Another appropriate oxide that is frequently employed to develop chemiresistors is non-stoichiometric In_2_O_3_. Therefore, Liu et al. [133] tried to develop In_2_O_3_/Ti_3_C_2_T_x_ composite materials fabricated from the interaction of hydrothermally pre-synthesized In_2_O_3_ nanocubes and Ti_3_C_2_T_x_ MXene via an additional hydrothermal treatment. In order to anchor and evenly distribute these porous cubes on the MXene sheet surface for producing multiple heterostructures, the authors modified the indium oxide surface with a cationic surfactant, (3-aminopropyl)triethoxysilane (Figure 26).

While exposing such a sensor to VOCs (acetone, xylene, triethylamine, methanol, trimethylamine, and toluene) of 5 ppm concentration at RT, it was found that the nanocomposite exhibits an enhanced sensitivity to methanol with the response of the *n*-type, which can be explained by the minor Ti_3_C_2_T_x_ addition. Still, the kinetic properties were found promising for practice; the response/recovery times were 6.5 s and 3.5 s, respectively, although some baseline drift has been observed too [133].

**Figure 27:**
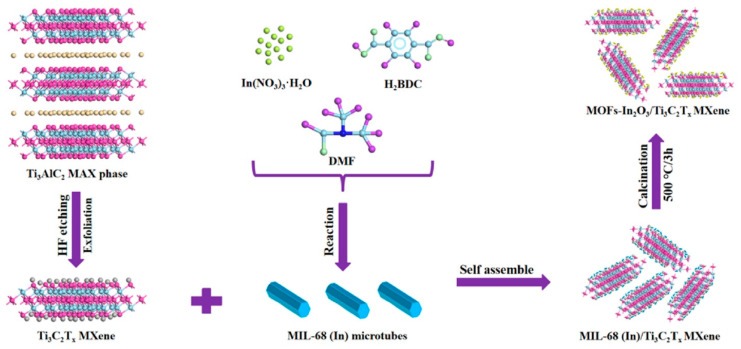
The synthetic illustration of the MOFs–derived In_2_O_3_/Ti_3_C_2_T_x_ MXene composites. Reprinted with permission from [134]. Copyright 2022: Elsevier B.V.

A self-assembly of metal organic frameworks (MOFs) was employed in study [134] to prepare an In_2_O_3_/Ti_3_C_2_T_x_ composite with indium oxide having a highly developed cage-like porous structure. For this purpose, an aqueous dispersion of Ti_3_C_2_T_x_ MXenes was introduced into a solution containing indium nitrate and terephthalic acid in dimethylformamide, followed by heat treatment at 120 °C (Figure 27). As a result, MOFs of the MIL-68 (In) type were formed, whose crystals have a hexagonal microtube shape with a small amount of MXene sheets distributed in their volume. To remove the organic moieties, the precursors were calcined in air at 500 °C to design the In_2_O_3_/Ti_3_C_2_T_x_ composite with a giant specific surface area of 447 m^2^/g. It was established [134] that the detection of NH_3_ at a 5 ppm concentration for such a sensor follows the *n*-type response characterized by a resistance reduction. Increasing the operating temperature from 25 °C to 100 °C facilitates some raising of the response from ca. 60.6% to ca. 67.5% but has a negative effect on the response/recovery times. Under heating up to 200 °C, there is a sharp deterioration in kinetics accompanying the reduction of the response to ca. 41.2%. The In_2_O_3_/Ti_3_C_2_T_x_ porous sample also yielded a good response to methanol (equal to ca. 20.2%) and ethanol (equal to ca. 12.8%) at 5 ppm concentration; the response to other VOCs, CO and NO_2_, at the same concentration, did not exceed ca. 9%. It is interesting that the sensor response has not changed significantly in a moderately humid atmosphere, RH ≤ 60%, although the higher humidity level resulted in decreasing both the resistance and the response. Obviously, such an effect relates to filling adsorption centers with H_2_O molecules, as discussed in other works.

In order to understand the effect of varying ratios of components in the Ti_3_C_2_T_x_/In_2_O_3_ nanocomposite on its gas-sensing performance at RT, Zhou and colleagues [135] obtained a series of samples containing In_2_O_3_ in a range of 15 wt.% to 80 wt.%. For this purpose, In_2_O_3_ nanopowder synthesized by the solvothermal method in glycerol and isopropyl alcohol to be calcined at 500 °C was introduced into the dispersion of multilayer Ti_3_C_2_T_x_ MXene synthesized under the NaF–HCl system.

This reaction system was subjected to ultrasonic processing for 5 h, which additionally led to some delamination of Ti_3_C_2_T_x_, as confirmed by the obvious shift of the (002) reflection to the low-angle region when compared with the original multilayer MXene. Testing the Ti_3_C_2_T_x_/In_2_O_3_ samples by introducing 30 ppm of NH_3_ allowed authors to select the composition containing 50 wt.% In_2_O_3_ [135] because its response value was the highest, equal to ca. 63.8% at RH = 40%, and was almost two times greater than that for the second gas-sensitive sample of Ti_3_C_2_T_x_–30 wt.% In_2_O_3_. The introduction of even minor amounts of In_2_O_3_, such as 15 wt.%, promoted the sensitivity towards ammonia as compared with pristine In_2_O_3_ and MXene by 2.7 and 12.5 times, respectively. At the same time, the authors note that the humidity level caused an almost linear reduction of the response value down to ca. 36.5% observed at RH = 85% in the case of the Ti_3_C_2_T_x_–50 wt.% In_2_O_3_ sample. The measured responses to 30 ppm of other gases (methanol, nitrogen monoxide, hydrogen sulfide, ethanol, and acetone) were in the range of 3.2–8.9%, which indicates an elevated selectivity of this sensor specifically versus ammonia, at least in the 2–100 ppm concentration range. When discussing the mechanism of ammonia detection by the Ti_3_C_2_T_x_/In_2_O_3_ nanocomposite, the authors considered that the NH_3_ injection resulted in higher resistance, while the individual In_2_O_3_, as a typical *n*-type semiconductor, should exhibit in such a case an opposite change in resistance. Therefore, it is concluded that the sensitivity of Ti_3_C_2_T_x_/In_2_O_3_-based sensors is mainly dependent on Ti_3_C_2_T_x_. Summarizing, the authors attributed the improved sensing performance of the composite material when compared with the individual components to two aspects:

1. Owing to the deposition of nanoparticles on the surface and in the interlayers of multilayer MXenes, a larger specific surface area appears to be 39.06 m^2^/g compared with the 6.95 m^2^/g characterizing one for pristine Ti_3_C_2_T_x_. This promotes the adsorption and diffusion of gas molecules into the volume of the receptor material.
2. When mixing semiconducting In_2_O_3_ and Ti_3_C_2_T_x_, which is considered a metallic-like crystal because of its high conductivity and charge carrier mobility, a Schottky barrier and an electron-depleted layer are formed at the interface. When exposed to ammonia, its molecules are predominantly adsorbed on the In_2_O_3_ surface, where ion-adsorbed forms of oxygen from air (O_2_^−^) are present due to their higher adsorption energy. These species interact according to the Reaction Scheme (24):

4NH_3_ + O_2_^−^ → 4NO + 6H_2_O + 5ē (24)

As a result, a large number of free electrons are released to be recombined with holes on the Ti_3_C_2_T_x_ surface, which increases the integral resistivity of the Ti_3_C_2_T_x_–50 wt.% In_2_O_3_ material.

**Figure 28:**
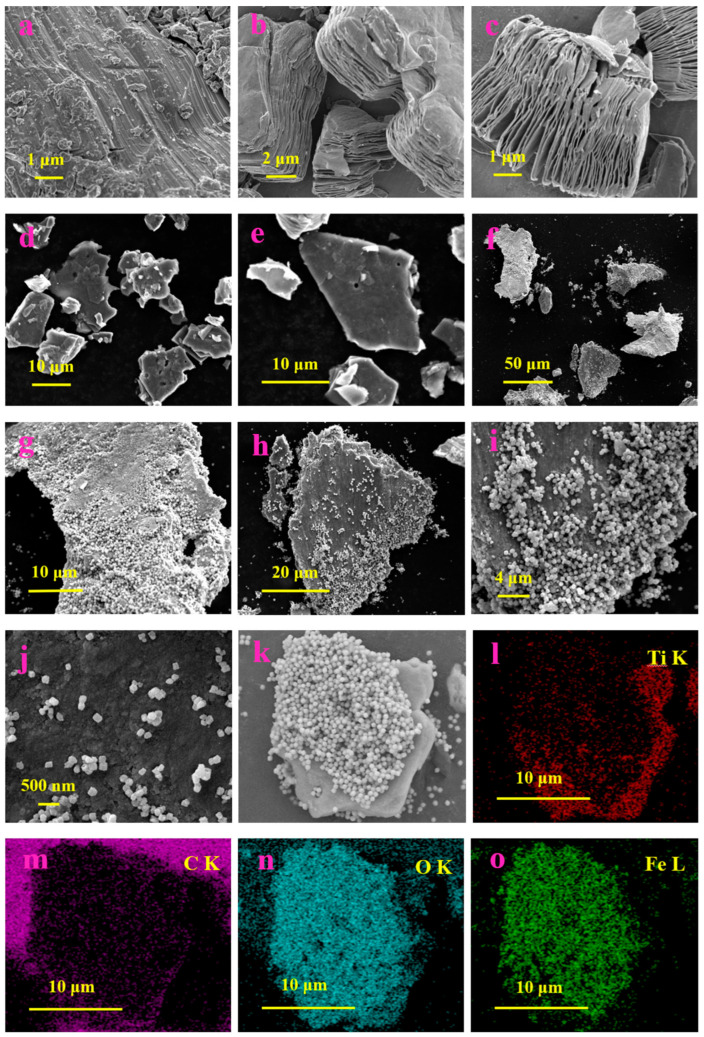
SEM images of (**a**) pristine MAX phase, (**b**,**c**) multilayer Ti_3_C_2_T_x_ MXene, (**d**,**e**) sheet-like Ti_3_C_2_T_x_ MXene, and (**f**–**j**) α-Fe_2_O_3_/Ti_3_C_2_T_x_ MXene composites; (**k**–**o**) EDS elemental mapping micrographs of α-Fe_2_O_3_/Ti_3_C_2_T_x_ MXene composites. Reprinted with permission from [136]. Copyright 2021: Elsevier B.V.

The next metal oxide worth testing in combination with MXene to develop a sensor is Fe_2_O_3_. The method employed earlier to compose In_2_O_3_/Ti_3_C_2_T_x_ [133] was applied in the study described in Ref. [136] to compose composites consisting of α-Fe_2_O_3_ nanocubes anchored to the surface of the multilayer Ti_3_C_2_T_x_ MXene as a result of electrostatic self-assembly (Figure 28). When examining the gas-sensing properties of such α-Fe_2_O_3_/Ti_3_C_2_T_x_ composite at RT and a relative humidity of approx. 22.3%, taking 5 ppm of some analytes (toluene, methanol, formaldehyde, acetone, xylene, ethanol, SO_2_, and NO_2_), the authors revealed its higher sensitivity to acetone with a ca. 16.6% response, while the second largest response was to xylene, at ca. 3.5%. The reducing character of the resistance change when detecting acetone vapors indicates that α-Fe_2_O_3_ as an *n*-type semiconductor plays the most significant role in this process. High rates of response/recovery were also observed.

**Fe_2_O_3_**. Liu et al. [137] reported on the hydrothermal synthesis of α-Fe_2_O_3_/Ti_3_C_2_T_x_ composites, where the rose-like α-Fe_2_O_3_ particles were grown on the surface of MXene sheets. In this case, isopropanol and a dispersion of delaminated Ti_3_C_2_T_x_ were added to a solution of iron(III) chloride and urea in ethylene glycol, followed by a heat treatment at 120 °C. Because such mild conditions do not facilitate the appearance of iron oxide but rather α-FeOOH metahydroxide, the produced and purified product was calcined in an Ar atmosphere at 500 °C, which excluded a significant oxidation of the 2D nanomaterial. Measuring the response of the material to 5 ppm of several VOCs (acetone, methanol, trimethylamine, ethanol, toluene, NH_3_, SO_2_, and NO_2_) at RT and 22 rel.% of humidity revealed an increased sensitivity to ammonia, with the response equal to ca. 18.3%. Fast response and recovery, as well as the fact that the resistance decreased rather than increased upon the analyte intake, also indicate the predominance of α-Fe_2_O_3_ in the detection mechanism. The role of Ti_3_C_2_T_x_ is limited to forcing an enlarged nanocomposite specific surface area due to spatially-restricted aggregation of FeOOH nanoparticles, as well as enhanced charge carrier mobility and the formation of n-p-heterojunction at the interphase boundaries [137].

A similar approach to the synthesis of porous semiconductor oxide on Ti_3_C_2_T_x_ layer surfaces via intermediate MOF self-assembly, which was described for the In_2_O_3_/Ti_3_C_2_T_x_ structure in [134], was also employed in another research [138] to synthesize the Fe_2_O_3_/Ti_3_C_2_T_x_ composite, where the iron oxide was present in two phases, α- and γ-Fe_2_O_3_. The MXene content in the output compositions ranged from 2 to 6 wt.%. The gas-sensing properties of the material were measured by applying the receptor layer to the ceramic tube with a heater confined in the inner space. For the composite material containing 4 wt.% of Ti_3_C_2_T_x_, the increase in the specific surface area fixed by the BET method according to XPS data was accompanied by a significant growth in the relative percentage of oxygen vacancies, equal to ca. 19.8%, as compared with pristine α-/γ-Fe_2_O_3_, of ca. 12.6%. Testing these materials at 100 ppm of acetone in the temperature range of 160–310 °C showed [138] that the optimal temperature for all samples, except for pristine Ti_3_C_2_T_x_, is 255 °C. However, for the sample containing 4 wt.% of the MXene, the signal value exceeds that for α-/γ-Fe_2_O_3_ by eight times at close response and recovery rates. This sample also showed maximum selectivity with respect to acetone detection; its response exceeded the results for other VOCs, including ethanol, methanol, formaldehyde, and xylene, by 3–6 times, which the authors attributed to the differences in bond dissociation energies of these molecules.

Discussing the detection mechanism and, therefore, the reasons for the improved sensory properties of the Fe_2_O_3_-4 wt.% Ti_3_C_2_T_x_ composite, the authors summarize [138] that there is a large number of oxygen vacancies at the γ-Fe_2_O_3_ surface, such as titanium defects in MXene, which can serve as adsorption centers. MXene surface groups (–OH, –O, and –F) can interact with acetone molecules by forming hydrogen bonds, while the Ti_3_C_2_T_x_ core has a high conductivity, which contributes to the transport of charge carriers. Additionally, the Schottky barrier formed at the interface contributes to the transition of oxygen molecules adsorbed on the surface into the ionic state. The authors also noted that a partial Fe^3+^ reduction of the γ-Fe_2_O_3_ phase may occur in a reducing atmosphere, leading to the formation of Fe_3_O_4_ clusters with lower resistance, which are oxidized back to Fe_2_O_3_ in the air (Reaction Scheme (25)).

The presence of iron ions with different degrees of oxidation facilitates electron transport between defects.

(25) $$\gamma -Fe_{2}^{3+}O_{3}^{2–}\overset{Reduction}{\to }\underset{Oxidation}{\leftarrow }\;Fe^{2+}O^{2–}+Fe_{2}^{3+}O_{3}^{2–}$$

**Figure 29:**
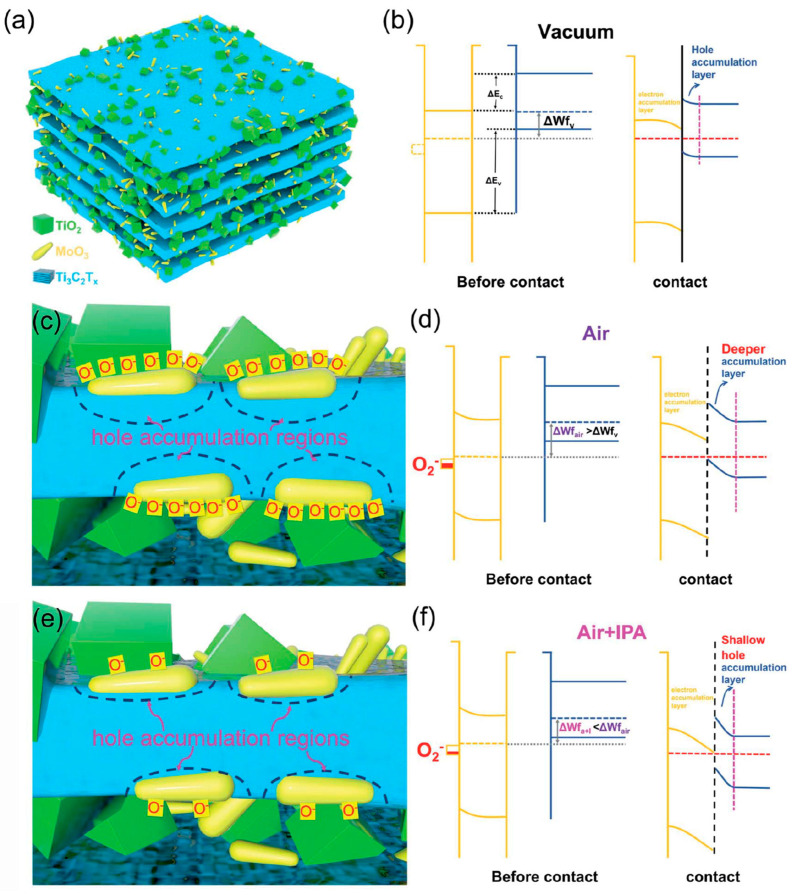
(**a**) Cartoon illustration of the MoO_3_/TiO_2_/Ti_3_C_2_T_x_ sensing layer units, (**b**) the energy band depiction of the situation before and after contact between MXene and MoO_3_ in the case of the flat band situation, (**c**) cartoon illustration of the effect of heterojunction formation on the MXene plates, in air, (**d**) the energy band depiction of the situation before and after contact between MXene and MoO_3_ in air, (**e**) cartoon illustration of the effect of heterojunction formation on the MXene plates, under isopropanol exposure, and (**f**) the energy band depiction of the situation before and after contact between MXene and MoO_3_ under isopropanol exposure. Reprinted with permission from [139]. Copyright 2022: The Royal Society of Chemistry.

**Combination of Two *n*-Type Semiconductor Oxides**. Some publications draw quite reasonable attention not only to the role of an added semiconducting metal oxide but also to the possibility of Ti_3_C_2_T_x_ partial oxidation resulting in TiO_2_ nanoparticle formation at some stages of the composite material production process, e.g., hydrothermal/solvothermal synthesis of MO_x_ in the presence of MXene or thermal treating samples in air, which is not mentioned in most papers on the preparation and study of gas-sensitive properties of nanocomposites. For instance, Yao et al. highlight [139] the formation of the TiO_2_ anatase phase during a hydrothermal treatment of the “(NH_4_)_6_Mo_7_O_24_·4H_2_O–H_2_O–HCl–Ti_3_C_2_T” reaction system at 180 °C. This is due to the fact that the MXene oxidation leads not only to sensory property changes caused by appearing heterojunctions at the interface but often results in a significant increase in the resistance of the Ti_3_C_2_T_x_/TiO_2_/MoO_3_ composite, complicating or blocking the possibility of chemiresistive response measurement. In this work, the effect of MoO_3_ content was also examined by varying the initial reagent content ((NH_4_)_6_Mo_7_O_24_: 0–50 wt.%) with respect to the Ti_3_C_2_T_x_ MXene. It was found that the introduction of MoO_3_ with a large number of vacancies reduces the Ti_3_C_2_T_x_/TiO_2_ electrical conductivity and enhances the responses to various VOCs (acetone, isopropanol, toluene, methanol, ethanol, and ammonia) at RT by 20–40 times. The higher resistance of the receptor materials upon interaction with these gases indicates that Ti_3_C_2_T_x_ dominates in their detection. At the same time, there is an enhanced sensitivity to isopropanol, especially for the material with an initial ammonium molybdate content of 30 wt.%; the response to 50 ppm of C_3_H_9_OH at RH = 50% is 245%. The authors propose that this feature belongs to the optimal microstructure of the composite, with robust electronic bonding between MXene sheets and surface dopants, while MoO_3_ excess wraps the MXene, causing a loss of its layered structure and weakening the interaction between the gas molecules and the sensor. It is noted [139] that since the resistance increases upon reducing gas adsorption, the charge carriers are holes, which are frequently characteristic for MXenes, whose number goes down as additional electrons appear due to the interaction of the material with gas molecules. The presence of defective MoO_3_ on the nanocrystal surface simplifies the access of analytes to Ti_3_C_2_T_x_ layers, while the large number of oxygen-deficient sites, centers of interaction with atmospheric oxygen, also improves the sensitivity. Relying on the experimental data, the authors believe [139] that the Ti_3_C_2_T_x_-MoO_3_ heterojunction (Figure 29) plays a determining role in improving the sensory properties, while the influence of the Ti_3_C_2_T_x_-TiO_2_ heterojunction is rather limited. The authors attribute the latter effect to the low reactivity of TiO_2_ and the fact that the crystallite size of this phase exceeds the Debye length of titanium dioxide. Due to the difference between the values of the work function of MoO_3_ and Ti_3_C_2_T_x_, 5.1 eV and 3.4 eV, respectively, a near-surface layer accumulating electrons in the case of MoO_3_ and holes in the case of Ti_3_C_2_T_x_ is formed as a result of electron transfer from MXene to MoO_3_. A negative charge growth at the MoO_3_ surface due to the ion sorption of oxygen molecules leads to an evolution of the difference between its work function and that of Ti_3_C_2_T_x_, as well as the corresponding energy band bending and broadening of the charge carrier accumulation layers. When the nanocomposite comes in contact with the gas, the negative charge of the surface decreases and the hole accumulation layer becomes narrower, i.e., the resistance of the material grows.

Wu and colleagues [140] synthesized the composite material of Ti_3_C_2_T_x_/TiO_2_/SnO_2_ by mixing aqueous suspensions of previously synthesized Ti_3_C_2_T_x_ and SnO_2_ nanofibers, followed by a long exposure of the resulting complex dispersion at 80 °C, which aimed at partial oxidation of MXene. The oxidation was confirmed experimentally by XRD, HRTEM, and XPS data. The mass ratio of Ti_3_C_2_T_x_ to SnO_2_ was 2:1. It was found that the introduction of electrospun SnO_2_ nanofibers into the composite resulted in an enhanced response to all the gases under test (NH_3_, acetone, ethanol, and H_2_, NO_2_), not only compared with the individual components but also with respect to the Ti_3_C_2_T_x_/TiO_2_ composite, which the authors explain by the suitable energy band structure of the three phases. A higher sensitivity towards nitrogen dioxide was revealed: the response to 10 ppm of NO_2_ was ca. 32.4%. It was observed that the response pattern to oxidizing and reducing gases of various chemical structures with increasing resistance indicates the detection mechanism is mainly related to the surface charge transfer model [140]. In other words, the surface of the Ti_3_C_2_T_x_/TiO_2_/SnO_2_ heterostructure retains the functional groups characteristic of the Ti_3_C_2_T_x_ MXene, which provide multiple active adsorption centers and fast electron transport channels at RT.

Additionally, several types of heterojunctions, SnO_2_–TiO_2_ and Ti_3_C_2_T_x_–TiO_2_, are generated and can regulate the energy band structure of Ti_3_C_2_T_x_.

Among *n*-type semiconductor metal oxides, indium and iron (III) oxides can also be highlighted, as they have been used to produce nanocomposites that are promising to employ in chemiresistive gas sensors.

Summarizing the data analyzed on the sensory properties of nanocomposites based on Ti_3_C_2_T_x_ and *n*-type semiconductor metal oxides (MO_x_), we might distinguish two cases:

(1) Mxene doped with nanosized metal oxide particles;
(2) Semiconductor metal oxide modified with MXene.

There is an improvement in sensor properties in both cases due to an enhancement of the specific surface area because MO_x_ nanoparticles in the MXene interlayer space prevent their spontaneous agglomeration during coating application while MXene sheets limit the formation of large MO_x_ aggregates during their synthesis. Owing to the presence of polar functional groups on the Ti_3_C_2_T_x_ surface, new phase nucleation occurs on them, whose coalescence is further prevented by the MXene planes. This leads to a higher number of adsorption centers and, consequently, more sensitivity.

The formation of a heterojunction between MO_x_ semiconductor particles and Ti_3_C_2_T_x_ having metallic conductivity makes it possible to implement a charge separation at the interface and, therefore, to promote the response upon gas analyte intake.

In the case of the prevailing sensing mechanism of *n*-type semiconducting metal oxide, the introduction of MXene, which has high conductivity, promotes charge carrier transport, resulting in decreased sensor response and recovery times as well as a lower detection temperature. Moreover, the presence of –O, –OH, and –F functional groups on the surface of MXene sheets, as well as defects, improves analyte gas sorption, especially for those capable of forming hydrogen bonds.

#### 4.1.2. Modification with *p*-Type Metal Oxide Semiconductors

**CuO and Cu_2_O.** In contrast to the huge amount of scientific research on MXene composites containing *n*-type semiconducting metal oxides, the number of papers where Ti_3_C_2_T_x_ or Ti_2_CT_x_ MXenes are modified with *p*-type semiconductor oxide is drastically smaller. For this review, we managed to collect articles describing the sensor properties of composite materials based on Ti_3_C_2_T_x_ and CuO, Co_3_O_4_, lithium, sodium, and potassium tungstate, as well as defective WO_3_:Cr.

To improve primarily the sensitivity and kinetic characteristics of CuO–based sensors, which were found to be highly sensitive to H_2_S and VOCs, including BTEX, Hermawan and colleagues [141] fabricated CuO/Ti_3_C_2_T_x_ composites with a MXene content ranging from 10 wt.% to 40 wt.% by electrostatic assembly under ultrasound treatment of the joint dispersion of components. Studies of gas-sensing properties toward toluene (10–50 ppm) showed that the CuO—30 wt.% Ti_3_C_2_T_x_ composition has the best performance due to the highest specific surface area. It was also highlighted that advancing the operating temperature from 100 °C to 250 °C, facilitates the response value, R/R_0_, growing from ca. 1.2 to ca. 11.4 in the case of a 50 ppm concentration of toluene. This value exceeded the one characteristic for pristine CuO by almost five times. The described CuO/Ti_3_C_2_T_x_ composite material showed slightly lower sensitivity to other VOCs; the responses to ethanol, acetone, methanol, and hydrogen at 50 ppm concentration were 7.3, 7.2, 5.4, and 2.8, respectively. The authors have demonstrated experimentally the advantage of CuO hybridization with accordion-like Ti_3_C_2_T_x_ compared with other 2D materials, in particular MoS_2_ and rGO [141]. The humidity, however, still dramatically deteriorates the CuO—30 wt.% Ti_3_C_2_T_x_-based sensor performance. When the RH value increased from 0% to 70%, the response to 50 ppm toluene got lower, from ca. 11.4 to ca. 2.1. This is again explained by the competition between adsorbing toluene and water molecules for the active centers of the receptor material.

**Figure 30:**
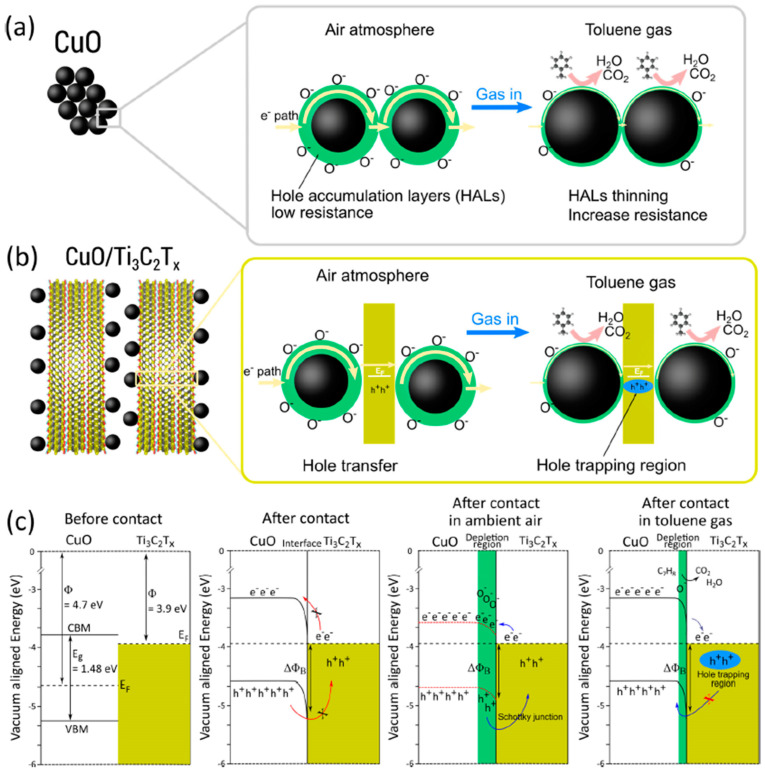
Gas-sensing mechanism of (**a**) pristine CuO nanoparticles and (**b**) CuO nanoparticles/Ti_3_C_2_T_x_ MXene hybrid heterostructures. (**c**) Band structure alignment of CuO/Ti_3_C_2_T_x_ before contact, after contact, in ambient air, and in toluene gas. Reprinted with permission from [141]. Copyright 2020: American Chemical Society.

The improvement of CuO/Ti_3_C_2_T_x_ sensor performance is explained by the authors [141] via the appearance of a Schottky barrier at the interface due to the difference in work functions, which are 3.9 eV and 4.7 eV for Ti_3_C_2_T_x_ and CuO, respectively. Such a barrier facilitates a hole trapping region (HTR) on the MXene side (Figure 30), characterized by a higher amount of ion-sorbed oxygen species (O^−^, specific for the chosen temperature). The interaction of toluene molecules with the O^−^ ions on the CuO surface leads to a reduction in their surface concentration and, consequently, a narrowing the depletion region (hole accumulation layers) and a diminishing of sensor resistance. The authors also highlight the supporting role of MXene as a conductive layer for the faster charge carrier mobility through a network of CuO nanoparticles, which contributes to a shorter response/recovery time [141].

In addition, the research [142] showed the promising prospects for developing a self-powered ammonia sensor for pork quality control based on CuO obtained as a result of the thermal destruction of Cu-containing MOF modified with a minor amount of accordion-like Ti_3_C_2_T_x_.

**Figure 31:**
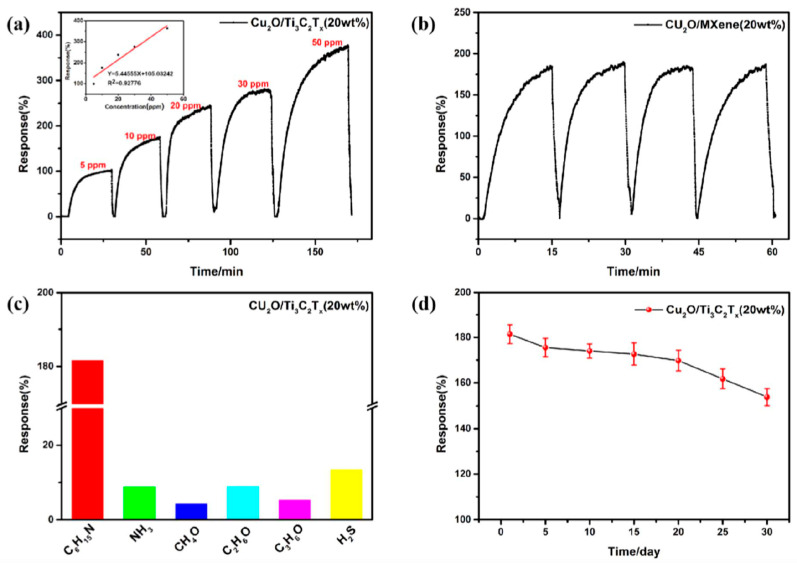
(**a**) Dynamic response/recovery curve of the Cu_2_O/Ti_3_C_2_T_x_ (20 wt.%) gas sensor to different concentrations of TEA gas, (**b**) Reproducibility of Cu_2_O/Ti_3_C_2_T_x_ (20 wt.%) gas sensor on successive exposures (four cycles) to 10 ppm TEA at RT, (**c**) Selectivity to 10 ppm different gases of the Cu_2_O/Ti_3_C_2_T_x_ (20 wt.%)-based gas sensor at RT, (**d**) long-term stability of the Cu_2_O/Ti_3_C_2_T_x_ (20 w.t%)-based gas sensor. Reprinted with permission from [143]. Copyright 2022: IOP Publishing Ltd.

Zhou and colleagues [143] described the modification of the *p*-type semiconducting Cu_2_O, with an energy band gap of 2.17 eV, by Ti_3_C_2_T_x_ MXene, 10–50 wt.%, in order to increase its sensitivity to triethylamine. For this purpose, hollow Cu_2_O nanospheres produced by reducing Cu^2+^ with NH_2_OH·HCl in the presence of sodium dodecyl sulfate (SDS) while adding sodium hydroxide were dispersed under ultrasound exposure along with accordion-like Ti_3_C_2_T_x_. Detecting 10 ppm of trimethylamine (TEA) at RT and 50 rel.% humidity showed that the composition containing 20 wt.% MXene exhibited the highest sensitivity to TEA, with a response equal to ca. 181.4%, which was almost 3.5 times higher than that of pristine Cu_2_O. At the same time, it was noted that doping allowed one to reduce the sensor response time by three times. It was found that a change in humidity from 30 rel.% to 90 rel.% decreased the response from 220% to 109%. Measuring the response of Cu_2_O-20 wt.% Ti_3_C_2_T_x_ material to 10 ppm of other gas analytes (ammonia, methanol, ethanol, acetone, and hydrogen sulfide) exhibited that even the largest response to H_2_S was 13.4%, while the response to other gases did not exceed 9%, which indicates a high selectivity toward triethylamine (Figure 31).

However, when testing the material for 30 days, the response decreased by 15%, displaying a continued degradation of the functional properties of the receptor material. Still, the authors explain advancing the Cu_2_O sensing properties in the presence of Ti_3_C_2_T_x_ by the growth of a specific surface area, as well as the acceleration of carrier transport because of their high mobility in MXene and the formation of a Schottky barrier at the composite interfaces. 

**Figure 32:**
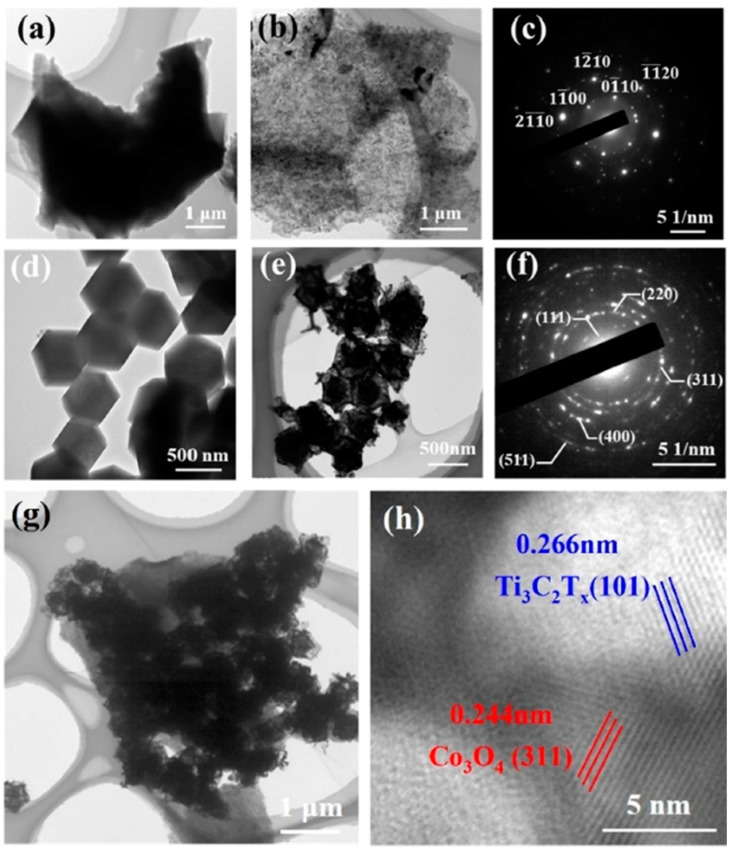
TEM images of (**a**) multi-layer Ti_3_C_2_T_x_ and (**b**) Ti_3_C_2_T_x_ nanosheets, (**c**) SAED image of Ti_3_C_2_T_x_, TEM image of (**d**) ZIF-67 and (**e**) Co_3_O_4_, (**f**) SAED image of Co_3_O_4_, and (**g**) TEM image of Co_3_O_4_/Ti_3_C_2_T_x_-2% nanocomposite. (**h**) HRTEM image of Co_3_O_4_/Ti_3_C_2_T_x_-2%. Reprinted with permission from [144]. Copyright 2022: Elsevier B.V.

**Co_3_O_4_**. Doping with MXene, but at a much lower content of Ti_3_C_2_T_x_, was also performed by Bu et al. [144] to synthesize highly dispersed Co_3_O_4_/Ti_3_C_2_T_x_ (1, 2, or 5%) composites. Similar to other works [134,138,142], the approach to obtain highly porous metal oxide semiconductors via MOFs’ decomposition/oxidation was applied. In the first step, rhombohedral dodecahedrons of organometallic frameworks (ZIF-67) are grown on the surface of delaminated MXene sheets during the interaction of cobalt(III) nitrate and 2-methylimidazole in an ethanol medium in the presence of Ti_3_C_2_T_x_ (Figure 32). After separation and drying, the product was calcined in air at 350 °C for 1 h to form a Co_3_O_4_/Ti_3_C_2_T_x_ nanocomposite. Gas-sensitivity screening of the materials on the example of 50 ppm ethanol in the temperature range of 150–275 °C allowed (1) to establish the optimal operating temperature of 200 °C and (2) to select Co_3_O_4_–2 wt.% Ti_3_C_2_T_x_ as the most sensitive composite; the response, R/R_0_, was equal to 190 at RH = 30%. It was also found that there was an 18-fold improvement in response and recovery times compared with pristine Co_3_O_4_. A long-term stability test of the composite material over 60 days showed a gradual decrease in sensitivity over this time by approx. 10%. The authors point out that Co_3_O_4_–2 wt.% Ti_3_C_2_T_x_ also has a higher sensitivity than Co_3_O_4_ with respect to other gases, such as methanol, isopropanol, and acetone; however, the response value to a 50 ppm concentration of these analytes does not exceed 71. At the same time, when the humidity increases from 30 rel.% to 90 rel.%, the response to 50 ppm of ethanol decreases from ca. 190 to ca. 160. The background of the gas detection mechanism seems to follow the concept of the interaction of adsorbed analyte molecules with ion-adsorbed species of oxygen on Co_3_O_4_ particle surfaces as a typical *p*-type semiconductor. The authors attribute improving the sensor performance of cobalt oxide with 2 wt.% Ti_3_C_2_T_x_ to (1) the formation of a heterojunction between Co_3_O_4_, a *p*-type semiconductor, and MXene, which has metallic conductivity, and (2) the role of Ti_3_C_2_T_x_ as a platform for uniform nucleation and formation of nanoporous Co_3_O_4_, which promotes an increased specific surface area of nanocomposites and a higher number of adsorption centers.

Sun et al. [145] used a hydrothermal method to develop a composite material composed of layered Ti_3_C_2_T_x_ and cobalt-aluminum layered double hydroxide (*n*(Co^3+^):*n*(Al^3+^) = 2:1) in a 3D hierarchical structure, which was further calcined in a N_2_ to grow Co_3_O_4_/Al_2_O_3_@Ti_3_C_2_T_x_ compositions. The XRD pattern of the latter displayed only Co_3_O_4_ reflections; the Raman spectra also yielded exclusively A_1g_, E_g_, and F_2g_ modes of the Co_3_O_4_ phase, while HRTEM images supplied Al_2_O_3_ interplanar distances as well, and the XPS spectrum of Ti2p was found to be similar to that of Ti_3_C_2_T_x_. While measuring 100 ppm of NO_x_ at RT and RH = 26%, the sensitivity was noted not only for the Co_3_O_4_/Al_2_O_3_@Ti_3_C_2_T_x_ composite but also for the precursor containing cobalt-aluminum layered hydroxide. However, the response value of the composite is found to be significantly higher. Raising the humidity from 26 rel.% to 75 rel.% leads to a chemiresistive signal, R_0_/R, lowering from ca. 40.3 to ca. 10.7. The improved sensing properties of Co_3_O_4_/Al_2_O_3_@Ti_3_C_2_T_x_ are explained by the synergy of three factors:

− Interaction of Co_3_O_4_ and Ti_3_C_2_T_x_ phases, promoting electron transfer, and increasing the diffusion rate of charge carriers;
− Three-dimensional structure of the receptor material, allowing channels for gas inflow that facilitates reducing the response/recovery time;
− High specific surface area and porous structure, leading to the formation of adsorption centers; the Al_2_O_3_ addition also promotes the appearance of defects, thus improving the adsorption capacity of the composite.

**Modification with *n*-Type Metal Oxide Semiconductors**. Ama et al. [146] studied the features of gas-sensing properties of composite materials in the K_2_W_7_O_22_–Ti_3_C_2_T_x_ system based on their previous findings on employing potassium tungstate (K_2_W_7_O_22_) toward RT acetone detection. Thus, to obtain the receptor material, they mixed dispersions of hydrothermally pre-synthesized potassium tungstate nanorods with MXene obtained by etching Ti_3_AlC_2_ MAX-phase with 5% HF under hydrothermal conditions. After dwell time while stirring the reaction system, which supported an electrostatic assembly, the composites, K_2_W_7_O_22_:Ti_3_C_2_T_x_, with different component ratios ranging from 1:5 to 9:1, were separated from the mother liquor, washed, and dried. Measurement of responses to 2.86 ppm of ethanol at RT under RH = 20% showed that the 2K_2_W_7_O_22_–1Ti_3_C_2_T_x_ composite exhibited the greatest sensitivity; the response was nearly 10 times higher than for pristine potassium tungstate. The authors suggested that this effect be facilitated by Ti_3_C_2_T_x_’s contribution to boosting electrical conductivity and charge transfer. In addition, the interaction between the components raises the contribution of the (002) face, which provides enhanced sensitivity of the *p*-type semiconductor K_2_W_7_O_22_ to ammonia at RT, as previously established elsewhere [147]. For the 2K_2_W_7_O_22_–1Ti_3_C_2_T_x_ composition, a high signal stability in a wide range of humidity, 10–86 rel.%, was also found.

The M_2_W_7_O_22_ nanowires of lithium, sodium, potassium, and chromium tungstate, as well as MXene-containing 2M_2_W_7_O_22_–1Ti_3_C_2_T_x_ nanocomposites, were synthesized by a similar method [148]. Testing of the receptor materials at 2.86 ppm of acetone allowed authors to identify hybrid MXene/Cr-doped tungsten oxide as the most promising material. The favorable properties of these structures are explained by the large number of active centers available for acetone molecule adsorption as well as by the stronger ferroelectric characteristics of the synthesized *p*-type oxide semiconductor. Moreover, the response of this receptor material is ca. 4.7 times higher than that of the 2K_2_W_7_O_22_–1Ti_3_C_2_T_x_ material described earlier [146]. A higher selectivity to acetone was noticed when compared with some other VOC vapors such as ethanol, methanol, toluene, and even water, whose response is almost two orders of magnitude lower.

The authors explain the improvement of sensory characteristics by a combination of several factors, including (i) the Schottky barrier formation at the interface between the semiconductor and MXene, (ii) the separation of charge carriers, (iii) chemical effects in terms of lower activation energy, catalytic activity, and surface reactions, and (iv) an increase in the specific surface area of the nanocomposite. Additionally, the characteristics of the underlying semiconductor material dominate as well.

**Table 2:** Sensory characteristics of nanocomposite coatings containing Ti_3_C_2_T_x_/Ti_2_CT_x_ MXenes and semiconducting metal oxides.

Gas Analyte	Concentration	Detection Conditions	Response (ΔR·100%/R_0_), %	Detection Limit	Receptor Material	Synthetic Features	Ref.
Doping component is an *n*-type semiconductor metal oxide							
	10 ppm	RT	+1.9	100 ppb	Ti_2_CT_x_/TiO_2_	LiF + HCl (12 M), 40 °C, 24 h, washing with HF (10%), +TiO_2_: Ti_2_CT_x_ incubation in ethanol-water mixture, 4 °C, 16 h	[99]
NO_2_	5 ppm	RT	+16.05	–	Ti_3_C_2_T_x_/TiO_2_	LiF + HCl (9 M), 35 °C, 24 h, +TiO_2_: Ti_3_C_2_T_x_ dispersion was maintained at 80 °C for 8 h	[103]
NO_2_	5 ppm	RT	−19.854	10 ppb	Ti_3_C_2_T_x_/TiO_2_/rGO	Mixing of Ti_3_C_2_T_x_ and GO dispersions (mass ratio 2:1).	[104]
Acetone	2 ppm	350 °C	−180	20 ppb	Ti_3_C_2_T_x_/TiO_2_	LiF + HCl (6 M), 35 °C, 24 h, +TiO_2_: Heat treatment of the coating in air at 350 °C, 24 h	[105]
Methanol			−110	35 ppb			
Isopropanol			−100	40 ppb			
Ethanol			−40	150 ppb			
Ethanol	100 ppm	RT	−22.47	10 ppm	Ti_3_C_2_T_x_/TiO_2_	LiF + HCl (38 wt.%), 60 °C, 24 h, US 20 min +TiO_2_: exposure to oxygen plasma in the MPCVD reactor at 500 °C, 1 h	[107]
Hexanal	100 ppm	RT	+8.8	217 ppb	Ti_3_C_2_T_x_/TiO_2_	HF (40 wt.%), 40 °C, 36 h, +TiO_2_: hydrothermal treatment of Ti_3_C_2_T_x_ water-ethanol suspension at 200 °C, 24 h	[110]
Acetone			+3.0	-			
Methanol			+3.5	-			
Benzene			+0.9	-			
NO_2_	100 ppm	RT	+4.455	-	Ti_3_C_2_T_x_/TiO_2_	HF (40 wt.%), RT, 24 h, +TiO_2_: hydrothermal treatment of Ti_3_C_2_T_x_ water-ethanol suspension at 180 °C, 20 h	[111]
SO_2_			+2.111	-			
NH_3_			+0.703	-			
CO			+0.667	-			
NO_2_		175 °C	+19.76	1 ppm			
Ammonia	30 ppm	RT, UV activation (365 nm)	~−41 *	5 ppb	Ti_3_C_2_T_x_/TiO_2_	HF (50 wt.%), RT, 2 h,+TiO_2_: hydrothermal treatment of Ti_3_C_2_T_x_ di spersion in 0.1M NaBF_4_ solution at 160 °C, 12 h	[113]
Ammonia	200 ppb	20 °C	+7.3	-	Ti_3_C_2_T_x_ (N-doped)/TiO_2_	LiF + HCl (6 M), 35 °C, 24 h, +TiO_2_: solvothermal treatment of Ti_3_C_2_T_x_ dispersion in urea-containing ethanol-ethylene glycol mixture at 200 °C, 18 h	[114]
Ethanol	10 ppm	230 °C	−5	0.5 ppm	SnO_2_/Ti_3_C_2_T_x_ (20 wt.%)	HF (40 wt.%), 60 °C, 48 h, +SnO_2_: hydrothermal treatment of K_2_SnO_3_·3H_2_O solution in the presence of urea and polyvinylpyrrolidone with the addition of Ti_3_C_2_T_x_ at 200 °C, 18 h	[118]
Trimethylamine	50 ppm	140 °C	−33.9 **	5 ppm	SnO_2_/Ti_3_C_2_T_x_	HF (40 wt.%), 40 °C, 24 h, +SnO_2_: hydrothermal treatment of a mixture of SnCl_4_·H_2_O solution with Ti_3_C_2_T_x_ and urea- and CTAB-containing solution at 140 °C, 24 h	[119]
NO_2_	10 ppm	25 °C, desorption—pulse heating up to 100 °C	+1.57	0.5 ppm	SnO_2_/Ti_3_C_2_T_x_	HF (40 wt.%), 35 °C, 24 h, +SnO_2_: hydrothermal treatment of Ti_3_C_2_T_x_ dispersion in water-ethanol medium containing SnCl_4_·H_2_O and 9% HCl, at 180 °C, 24 h	[120]
Ammonia	200 ppm	RT	+7.8 **	1 ppm	SnO/Ti_3_C_2_T_x_	HF_conc_, RT, 24 h, +SnO: hydrothermal treatment of US-activated Ti_3_C_2_T_x_ dispersion in SnCl_2_·2H_2_O aqueous solution containing urea and hydrochloric acid at 120 °C, 24 h	[121]
Acetone	100 ppm	RT	+12.1 *	10 ppm	SnO-SnO_2_/Ti_3_C_2_T_x_	HF_conc_, RT, 24 h, +SnO: hydrothermal treatment of US-activated Ti_3_C_2_T_x_ dispersion in SnCl_2_·2H_2_O aqueous solution containing urea and hydrochloric acid at 120 °C, 8 h	[112]
NO_2_	100 ppm	RT, RH = 70%	−41.6	-	Ti_3_C_2_T_x_ (crumpled spheres)/ZnO	Ultrasonic pyrolysis: ultrasonic spraying/drying of few-layered Ti_3_C_2_T_x_ dispersion in which polyvinylpyrrolidone and zinc acetate are dissolved, heat treatment in N_2_ at 800 °C	[122]
Ammonia			+4.2	-			
NO_2_	20 ppm	RT, desorbtion—UV	+367.63	0.5 ppm	Ti_3_C_2_T_x_/ZnO	HF, RT, 24 h, +ZnO: mixing and ultrasonic treatment of Ti_3_C_2_T_x_ dispersion with porous, layered ZnO nanopowder	[123]
NO_2_	20 ppb	RT, UV	+81	200 ppt	Ti_3_C_2_T_x_/ZnO	LiF + HCl (9 M), 40 °C, 24 h, +ZnO: (1) dispersion of Ti_3_C_2_T_x_ in Zn(CH_3_COO)_2_ methanol solution, addition of NaOH methanol solution, (2) the powder obtained in step 1 was dispersed and maintained in an aqueous NaOH solution along with ε-Zn(OH)_2_, 80 °C, 0.5 h	[124]
Acetone	100 ppm	320 °C	−14.1 *	20 ppm	ZnO/Ti_3_C_2_T_x_	HF, 30 °C, 24 h, delamination: TMAOH +ZnO: hydrothermal synthesis of ZnO from zinc acetate and CTAB with 2 wt% Ti_3_C_2_T_x_ MXene added	[125]
NO_2_	8 ppm	160 °C	+3.4 *	-	ZnO/Ti_3_C_2_T_x_	LiF + HCl (12 M), 38 °C, 48 h, +ZnO: solvothermal treatment of a system containing ZnCl_2_, sodium acetate, sodium citrate, and dispersed Ti_3_C_2_T_x_, in ethylene glycol	[126]
Formaldehyde	100 ppm	RT	+194.7	5 ppm	ZnSnO_3_/Ti_3_C_2_T_x_	HF (10 wt.%), 50 °C, 8 h, Delamination: DMSO, RT, 24 h, + ZnSnO_3_: hydrothermal treatment of a suspension containing pre-synthesized CTAB-treated ZnSnO_3_ with the addition of Ti_3_C_2_T_x_, 150 °C, 24 h	[127]
Ammonia	1 ppm	RT	+22.3	-	Ti_3_C_2_T_x_/WO_3_ (50 wt.%)	HF (40%), RT, 24 h, +WO_3_: Ultrasonic treatment of suspension obtained by mixing Ti_3_C_2_T_x_ and WO_3_ dispersions	[128]
NO_2_	200 ppb	RT, RH = 0–99%	+184	30 ppb	Ti_3_C_2_T_x_/WO_3_	HF (38%), RT, 24 h, sodium L-ascorbate treatment, +WO_3_: hydrothermal synthesis of WO_3_ from Na_2_WO_4_·2H_2_O, thioacetamide, and HCl in the presence of Ti_3_C_2_T_x_, 200 °C, 20 h	[129]
NO_2_	200 ppb	RT	+78%	15 ppb	WO_3_/Ti_3_C_2_T_x_ (20 wt.%)	HF (38%), RT, 24 h, delamination: DMSO, US, +WO_3_: hydrothermal synthesis of WO_3_ from Na_2_WO_4_·2H_2_O, thiourea, and HNO_3_ in the presence of Ti_3_C_2_T_x_, 180 °C, 24 h	[131]
Acetone	20 ppm	300 °C	−12.1 **	0.17 ppm	W_18_O_49_/Ti_3_C_2_T_x_ (2 wt.%)	HF, RT, 18 h, delamination: DMSO, US, +W_18_O_49_: solvothermal synthesis by heat treatment of WCl_6_ solution in ethanol with the addition of acetylacetone in the presence of Ti_3_C_2_T_x_, 150 °C, 24 h	[132]
Methanol	5 ppm	RT	−29.6	-	In_2_O_3_/Ti_3_C_2_T_x_	HF (40 wt.%), 35 °C, 6 h, delamination: DMSO, RT, 24 h, + In_2_O_3_: hydrothermal synthesis of In_2_O_3_ nanocubes from a solution of InCl_3_, sodium citrate, and urea, 140 °C, 24 h, In_2_O_3_ surface modification with surfactant (APTES), subsequent hydrothermal treatment after mixing with Ti_3_C_2_T_x_, 120 °C, 14 h	[133]
Ammonia	5 ppm	RT	−60.6	-	In_2_O_3_/Ti_3_C_2_T_x_	HF, 6 h, 35 °C, delamination: US,1 h, + In_2_O_3_: MOFs self-assembly upon thermal treatment of In(NO_3_)_3_ and terephthalic acid solution in DMF, 120 °C, 30 min, followed by annealing in air at 500 °C, 3 h	[134]
Ammonia	30 ppm	RT, RH = 40%	+63.8%	2 ppm	Ti_3_C_2_T_x_/In_2_O_3_ (50 wt.%)	NaF + HCl, 60 °C, 24 h, +In_2_O_3_: ultrasonic treatment (RT, 5 h) of a mixed aqueous suspension of Ti_3_C_2_T_x_ and In_2_O_3_ obtained by the solvothermal method from In(NO_3_)_3_ in glycerol-isopropanol solution	[135]
Acetone	5 ppm	RT, RH = 22.3%	−16.6	-	α-Fe_2_O_3_/Ti_3_C_2_T_x_	HF, 60 °C, 6 h, delamination: US, 80 min, +Fe_2_O_3_: Fe_2_O_3_ nanocubes synthesis by FeCl_3_ and NaOH mixing followed by precipitate aging, 100 °C, 4 days, modification of Fe_2_O_3_ surface with surfactant (APTES), subsequent hydrothermal treatment after mixing with Ti_3_C_2_T_x_, 120 °C, 14 h	[136]
Ammonia	5 ppm	RT, RH = 22%	−18.3	-	α-Fe_2_O_3_/Ti_3_C_2_T_x_	HF, 60 °C, 8 h, delamination: US, 1 h, +Fe_2_O_3_: solvothermal synthesis using a solution of FeCl_3_ and urea in ethylene glycol and isopropanol with Ti_3_C_2_T_x_ dispersion addition, 120 °C, 14 h, subsequent heat treatment at 500 °C, 2 h, Ar	[137]
Acetone	100 ppm	255 °C	−215.2 **	-	α-/γ-Fe_2_O_3_/Ti_3_C_2_T_x_ (4 wt.%)	LiF+HCl (6M), 35 °C, 24 h, DMF, US, 3 h, +α-/γ-Fe_2_O_3_: solvothermal treatment of Ti_3_C_2_T_x_ dispersion containing Fe(NO_3_)_3_ and terephthalic acid, 150 °C, 24 h, subsequent calcination, 450 °C, 4 h	[138]
Isopropanol	50 ppm	RT, RH = 50%	+245%	5 ppm	Ti_3_C_2_T_x_/TiO_2_/MoO_3_	NaF + HCl (9M), 35 °C, 24 h, US, Ar, 1 h, +TiO_2_/MoO_3_: hydrothermal treatment of (NH_4_)_6_Mo_7_O_24_·4H_2_O–H_2_O–HCl–Ti_3_C_2_T_x_” system, 180 °C, 24 h; the initial (NH_4_)_6_Mo_7_O_24_·4H_2_O content is 30% of Ti_3_C_2_T_x_	[139]
NO_2_	10 ppm	RT	+32.4%	0.1 ppm	Ti_3_C_2_T_x_/TiO_2_/SnO_2_	LiF + HCl, 70 °C, 36 h, US, 30 min, +TiO_2_/SnO_2_: aging of SnO_2_ nanofibers and Ti_3_C_2_T_x_ mixed dispersion, 80 °C, 10 h; mass ratio is 1SnO_2_-2Ti_3_C_2_T_x_	[140]
Doping component is an *p*-type semiconductor metal oxide							
Toluene	50 ppm	250 °C	+11.4 *	10 ppm	CuO/Ti_3_C_2_T_x_ (30 wt.%)	HF (40%), RT, 24 h, +CuO: ultrasonic treatment of CuO ethanol dispersion obtained by solvothermal method from copper acetate in anhydrous ethanol medium (150 °C, 12 h), with the addition of freshly prepared Ti_3_C_2_T_x_ powder, RT, 20 min	[141]
Triethylamine	10 pmm	RT, RH = 50%	+181.4	5 ppm	Cu_2_O/Ti_3_C_2_T_x_ (20 wt.%)	NaF+HCl, 60 °C, 48 h, +Cu_2_O: ultrasonic treatment of ethanol dispersion containing accordion-like Ti_3_C_2_T_x_ and Cu_2_O nanospheres derived from CuCl_2_ in the presence of SDS, NH_2_OH·HCl and NaOH, RT, 3 h	[143]
Ethanol	50 ppm	200 °C, RH = 30%	+190 *	1 ppm	Co_3_O_4_/Ti_3_C_2_T_x_ (2 wt.%)	HF (40%), 35 °C, 24 h, delamination: DMSO, 24 h, US, 1 h, +Co_3_O_4_: MOFs self-assembly on the Ti_3_C_2_T_x_ sheet surface out of Co(NO_3_)_3_ and 2-methylimidazole methanol solution, ultrasound, RT, 30 min, 24 h, subsequent calcination in air at 350 °C, 1 h	[144]
NO_x_	100 ppm	RT, RH = 26%	−40 **	0.01 ppm	Co_3_O_4_-Al_2_O_3_/Ti_3_C_2_T_x_	LiF, HCl(9M), 35 °C, 48 h, delamination: US, 30 min, Ar, +Co_3_O_4_-Al_2_O_3_: hydrothermal synthesis of cobalt-aluminum layered double hydroxide on Ti_3_C_2_T_x_ surface from dispersion containing Co(NO_3_)_3_, Al(NO_3_)_3_, urea, and polyvinylpyrrolidone, 90 °C, 6 h, subsequent heat treatment at 450 °C, 3 h, N_2_	[145]
Acetone	2.86 ppm	RT, RH = 20%	+250	-	2K_2_W_7_O_22_–1Ti_3_C_2_T_x_	HF (5 wt.%), hydrothermal treatment, 150 °C, 5 h, US, 1 h +K_2_W_7_O_22_: hydrothermal synthesis of K_2_W_7_O_22_ from Na_2_WO_4_·2H_2_O, oxalic acid, K_2_SO_4_, and HCl solution, 225 °C, 24 h, stirring of K_2_W_7_O_22_ + Ti_3_C_2_T_x_ dispersions, 12 h	[146]

* S = R/R_0_ [112,113,125,126,141,144]; ** S = R_0_/R [119,121,132,138].

### 4.2. Modification of Ti_3_C_2_T_x_ and Ti_2_CT_x_ by Semiconducting Metal Chalcogenides

A Ti_3_C_2_T_x_/MoS_2_–based sensor for detecting low NO_2_ concentrations at RT was developed by the authors in their study [149]. To obtain this composite, a dispersion of multilayer Ti_3_C_2_T_x_ MXene and MoS_2_ nanopowder synthesized from MoO_3_, KSCN, and HCl was hydrothermally treated [150]; the MoS_2_ content varied from 10 to 30 wt.%. SEM and TEM visualizations indicated that the MoS_2_ particles were distributed both on the Ti_3_C_2_T_x_ surface and in its interlayer space, which was confirmed by the broadening and shift of the (002) reflection towards smaller diffraction angles, from 9.7° to 8.6°. The increase in the interlayer distance of the MXene should have a positive effect on the generated electron transfer during gas detection. The gas-sensing properties of the obtained products were studied for NO_2_ in the concentration range of 10–100 ppm at RT (Table 3). It was established [149] that the optimum amount of dopant that advances the composite sensor performance is 20 wt.% MoS_2_. The response of Ti_3_C_2_T_x_–20 wt.% MoS_2_-structure to 10–100 ppm of NO_2_ is observed in the range of 35.8–72.5%, which is still affected by a significant baseline drift. The selectivity of the obtained coatings was justified with respect to methanol, ammonia, and NO_2_, at 50 ppm concentration. It was found that the response to NO_2_ was 60%, while for the other gases it did not exceed 10%. The authors do not explain the reason for such a high selectivity of the Ti_3_C_2_T_x_/MoS_2_-based composite to NO_2_, rather giving a conventional mechanism of oxidizing gas detection through the chemical adsorption of oxygen molecules with their further dissociation on the surface as well as physical adsorption of the analyte gas. Due to such processes, dissociated oxygen ions migrate easily from MXene to MoS_2_ and act as electron donors for NO_2_ binding.

**Figure 33:**
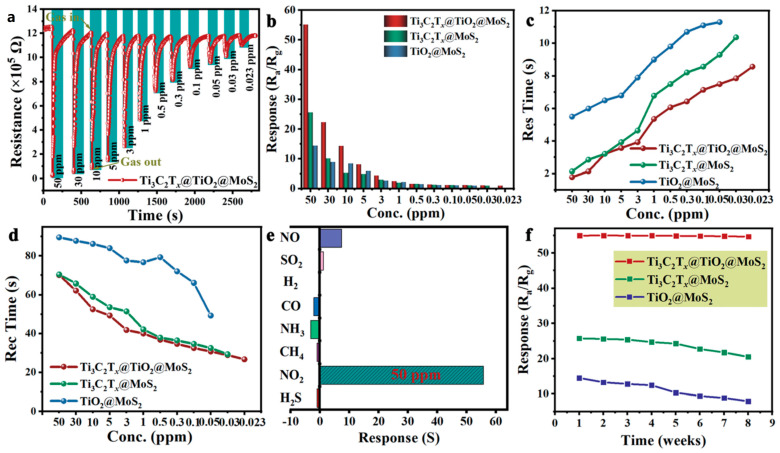
(**a**) Dynamic response curves of the Ti_3_C_2_T_x_/TiO_2_/MoS_2_ sensor to NO_2_ at RT (RH = 23.4%). (**b**) Response histogram of different sensors to NO_2_. (**c**,**d**) Response and recovery time curves of sensors. (**e**) Selectivity of Ti_3_C_2_T_x_/TiO_2_/MoS_2_ toward various gases under the same conditions. (**f**) Stability of different sensors at 50 ppm NO_2_. Reprinted with permission from [151]. Copyright 2022: The Royal Society of Chemistry.

The one-step in situ hydrothermal synthesis of 2D Ti_3_C_2_T_x_@TiO_2_@MoS_2_ composite was proposed in [151] to be employed for nitrogen dioxide detection in a wide concentration range, 0.023–100 ppm. For this purpose, a hydrothermal synthesis of MoS_2_ on the surface of delaminated Ti_3_C_2_T_x_ was carried out in the Na_2_MoO_4_·2H_2_O–CN_2_H_4_S reaction system. XRD data confirmed the formation of a complex Ti_3_C_2_T_x_/TiO_2_/MoS_2_ composite. TEM micrographs indicated the vertical growth of MoS_2_ particles on the surface of partially oxidized MXene, and the EDX analysis demonstrated the uniform distribution of molybdenum sulfide particles without obvious aggregation, which, according to the authors, is prevented by the formed TiO_2_-anatase particles. The integral area of the Ti-O peaks in the XPS spectra revealed the degree of Ti_3_C_2_T_x_ MXene oxidation to be around 86.7%. The impedance spectroscopy established that among the rows of Ti_3_C_2_T_x_/MoS_2_, MoS_2_, and Ti_3_C_2_T_x_/TiO_2_/MoS_2_, the highest conductivity is observed in the latter, which is associated with a high conductivity of MXene and with the presence of a large number of heterojunctions that contribute to the charge transfer. The negative slope of the Mott-Schottky plot determined the *p*-type conductivity of this nanocomposite. When examining the gas-sensing properties of the obtained coatings, the significant response to 50 ppm of NO_2_ (55%), the short response time (1.8 s), and the average recovery time (70 s) were observed, which the authors reasoned were due to (i) the high conductivity of the MXene component of the nanocomposite; (ii) the large number of heterojunctions at the grain boundaries, which accelerate electron transfer throughout the composite; and (iii) the vertical morphology of molybdenum sulfide growth and the introduction of defective structures, achieved by excess urea during synthesis. The experiments on finding the selectivity of the receptor material to NO_2_ versus H_2_, CO, CH_4_, NH_3_, H_2_S, and NO showed that the Ti_3_C_2_T_x_/TiO_2_/MoS_2_ nanocomposite exhibited, at high concentrations of 5–50 ppm, a considerably greater response to NO_2_; the closest signal corresponds to NO of 7.5% (Figure 33). The material was found to have good reproducibility and long-term stability over 8 weeks in combination with a linear response to a wide range of NO_2_ concentrations (R > 0.977).

The authors proposed an electron-hole mechanism of gaseous analyte detection for the composite [151]: when the sensor is exposed to air, O_2_ molecules capture electrons from the TiO_2_ electron accumulation layer to form chemically adsorbed O_x_^−^ oxygen forms. The adsorbed NO_2_ electrophilic molecule captures electrons at the nanocomposite surface and reacts with adsorbed oxygen species to form NO_3_^−^ particles, whose existence has been proven by IR spectroscopy. In this case, the electric field balance at the heterojunction is disturbed, leading to hole overflow into MoS_2_ to reach Fermi level equilibrium and decreasing the width of the heterojunction barrier.

**Figure 34:**
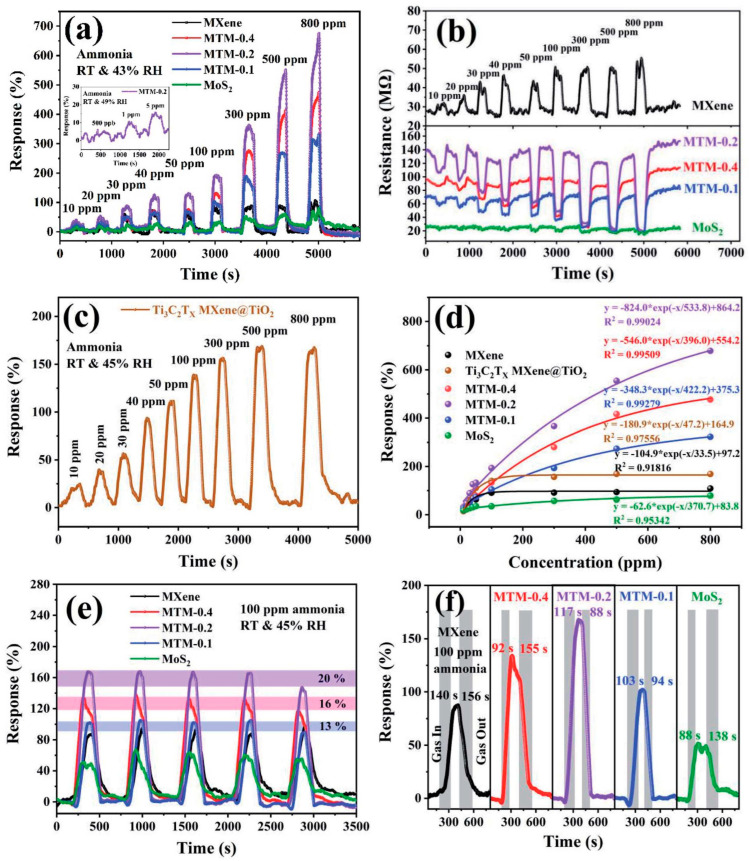
(**a**) Dynamic sensing characteristics of the prepared sensors to ammonia vapor at RT of 27 °C and RH of 43%; (**b**) resistance of the prepared sensors to different concentrations of ammonia vapor; (**c**) dynamic sensing characteristics of the Ti_3_C_2_T_x_ MXene@TiO_2_ sensor to ammonia vapor at RT of 26 °C and RH of 48%; (**d**) fitting equations of all prepared sensors between the ammonia response and gas concentration; (**e**) the reproducibility of the sensors to 100 ppm ammonia at RT of 27 °C and RH of 45%; (**f**) the response and recovery times for 100 ppm ammonia at RT of 27 °C and RH of 45%. Reprinted with permission from [152]. Copyright 2022: The Royal Society of Chemistry.

To detect ammonia in a wide concentration range, 10–800 ppm, Tian et al. synthesized the Ti_3_C_2_T_x_/TiO_2_/MoS_2_ composite material by the hydrothermal method described in [151], with the extra addition of citric acid hydrate to the reaction mixture as a surfactant [152]. Using a set of physicochemical analysis methods, the qualitative composition and morphology of the Ti_3_C_2_T_x_/TiO_2_/MoS_2_ nanocomposite were studied. MoS_2_ nanosheets and a small amount of TiO_2_ particles were grown on the surface of the Ti_3_C_2_T_x_ MXene. The authors observed that the TiO_2_ diffraction peak is significantly weaker in the MoS_2_-containing nanocomposite than in the Ti_3_C_2_T_x_/TiO_2_. They explain it by the MoS_2_ growth on the MXene surface, which inhibits TiO_2_ appearance under the hydrothermal synthesis. The Ti_3_C_2_T_x_/TiO_2_/MoS_2_ nanocomposite was found to have a higher response to NH_3_ at RT compared with the pristine components; a detection limit of 500 ppb was defined (Figure 34).

**Figure 35:**
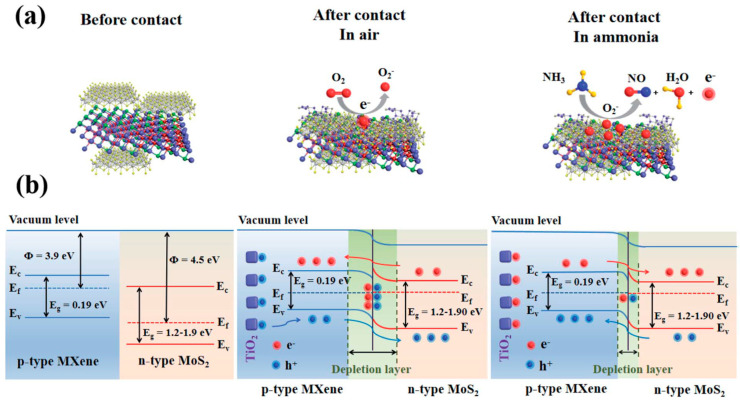
(**a**) Schematic illustration of the proposed gas-sensing mechanism; (**b**) energy band diagrams of the sensor comprosed of Ti_3_C_2_T_x_MXene@TiO_2_/MoS_2_. Reprinted with permission from [152]. Copyright 2022: The Royal Society of Chemistry.

It was noted [152] that the Ti_3_C_2_T_x_ MXene has *p*-type conductivity, while MoS_2_ is an *n*-type semiconductor. The resistance of the Ti_3_C_2_T_x_/TiO_2_/MoS_2_-based sensor is significantly higher compared with the initial components due to the newly formed p-n-heterojunctions. The high reproducibility within five cycles of the target gas and the long-term stability within 60 days supported the material’s ability to deliver a highly stable sensing signal. The selectivity tests carried out with ammonia, triethylamine, trimethylamine, n-butanol, acetone, and formaldehyde, all of 100 ppm concentration, and nitrogen dioxide, of 800 ppm concentration, supported the Ti_3_C_2_T_x_/TiO_2_/MoS_2_ nanocomposite’s ability to exhibit an exceptional selectivity to ammonia with a response equal to 173.3%, while that to other reducing gases did not exceed 15%. The response to the NO_2_ was 84%, but in another direction, *n*-type, maturing from the oxidizing nature of this analyte. Experiments on the humidity effect, where it was varied in the range of 43–75%, revealed a typical problem for MXene-based sendors as a drop in response values with increasing RH. The authors explained this by H_2_O molecules’ adsorption on the active centers and suggested that the interaction between adsorbed water molecules and oxygen with H^+^ and OH^−^ formation releases electrons into the conduction band of the sensing material, which suppresses the resistance. The ammonia detection for the Ti_3_C_2_T_x_/TiO_2_/MoS_2_ composite material is described via an electron-hole model by the creation of a surface depletion layer due to atmospheric oxygen adsorption [152]. It was found that MoS_2_ acts as the major phase material in the nanocomposite, as it exhibits the same resistance change trend as the pristine molybdenum chalcogenide. The energy band diagram of Ti_3_C_2_T_x_/TiO_2_/MoS_2_ nanocomposite shows (Figure 35) the energy band changes between Ti_3_C_2_T_x_ MXene and MoS_2_ when forming heterojunction accompanied by Fermi level alignment, while TiO_2_ mainly increases the distance between MXenes’ layers and provides more adsorption centers. The DFT method demonstrated that the ammonia molecules provide more charge transfer at the heterojunction of the nanocomposite than in the case of individual nanocomposite components, which leads to a greater change in resistance.

Chen et al. [153] synthesized a Ti_3_C_2_T_x_/WSe_2_ (2 and 4 wt.%) nanohybrid combining the properties of a semiconductor (WSe_2_) and MXene with a metallic conductivity. They were electrostatically assembled by mixing the dispersions of delaminated Ti_3_C_2_T_x_ and WSe_2_ nanoflakes, whose surfaces were modified with a cetyltrimethylammonium cation at elevated temperatures. This Ti_3_C_2_T_x_/WSe_2_ composite was studied upon exposure to 1–40 ppm of ethanol at RT. It was found that the 2 wt.% WSe_2_-containing nanocomposite exhibits *n*-type conductivity, has the highest and fastest response to the gas analyte, and has low electrical noise compared with the pristine compounds. In contrast, the individual MXene exhibits *p*-type conductivity resulting from an increase in resistance when exposed to the analyte gas. The long-term stability and reproducibility of the signal of the composite material over a month when exposed to 40 ppm of ethanol as well as the ultra-fast response (9.7 s) and recovery (6.6 s) at RT were revealed, which the authors ascribe to the presence of multiple heterojunctions in Ti_3_C_2_T_x_/WSe_2_. The stability of the receptor material to elevated humidity was studied in the RH range from 5% to 80%: the response to 40 ppm of ethanol at 80% RH was shown to drop by half from −12% (at 5% RH) to −6.1% (at 80% RH). Gas-sensing tests with ethanol, methanol, acetone, hexane, benzene, and toluene, all in a 40 ppm concentration, were carried out to reveal the advantages of the obtained nanohybrid in VOC detection [153]. A Ti_3_C_2_T_x_–2 wt.% WSe_2_-based coating was shown to exhibit high selectivity toward oxygen-containing VOCs. The authors do not provide a specific explanation for this effect, relying on the conventional detection mechanism of n-semiconductor materials involving an electron overflow due to surface redox reactions, which does not explain in this case the dominant interaction of oxygen-containing VOCs with the nanohybrid surface compared with hydrocarbon VOCs.

In another study [154], in situ synthesis of vertically oriented SnS nanoflakes has been reported in the form of inflorescence on the surface of conductive Ti_3_C_2_T_x_ followed by alkaline treatment with 0.05 M NaOH to modify the MXene surface functional groups. Solvothermal synthesis of SnS/Ti_3_C_2_T_x_ composite material was performed in the SnCl_2_·2H_2_O–thiourea–citric acid–ethylene glycol system at different component ratios and at different alkaline treatment times. Based on SEM and TEM analysis data, the optimal amount of MXene was determined to obtain a sample with a large specific surface area and a uniform distribution of SnS nanoflakes on the Ti_3_C_2_T_x_ surface. The authors explain the vertical orientation of sulfide nanoparticles during synthesis by the fact that surface titanium atoms with vacant orbitals tend to be coordinated by electron donor atoms, in this case sulfur. XRD data confirm the formation of SnS/Ti_3_C_2_T_x_ nanocomposite; however, it was found that alkaline treatment reduces the crystallinity of the material and increases the number of lattice defects (dislocations and microstrains). According to the XPS analysis, it was found that the alkaline treatment enhances the Sn^4+^ percentage from ca. 69.9% (before treatment) to ca. 83.8% (after treatment), which can indirectly indicate the successful modification of the surface with oxygen-containing functional groups. The obtained coatings were studied versus 1−10 ppm of NH_3_ at 25 °C and 20 rel.% of humidity [154]. The receptor material was found to exhibit typical *n*-type semiconductor conductivity based on the decrease in resistance when exposed to NH_3_. For the SnS/Ti_3_C_2_T_x_ nanocomposite-based sensor, a significant increase in sensitivity compared with pristine SnS and Ti_3_C_2_T_x_ was observed due to the larger specific surface area of vertically oriented sulfide particles and high N-S bond energy. In particular, the response value of the sample synthesized by additional alkaline treatment to 250 ppb of NH_3_ exceeded that of the sample without such a treatment even to 10 ppm of NH_3_. A selectivity test of the obtained coatings allowed the authors to distinguish ammonia: the response to 5 ppm of NH_3_ was significantly higher than that to 5 ppm of methanol, ethanol, acetone, isopropanol, NO_2_, and CO. Still, increasing the humidity level to 40 rel.% has not affected the gas-sensing performance of the coatings, although with a further rise in humidity, the stability of the signal and its magnitude are notably reduced. This effect was explained, firstly, by a lower effective concentration of NH_3_ due to its dissolution in water vapors and, secondly, by a decrease in the number of vacant adsorption centers for the target gas [154].

Thus, the researchers attribute in general the higher sensitivity of gas sensors based on MXene/transition metal chalcogenides nanocomposites to both the appearance of heterojunctions at the interface and the oriented growth of chalcogenides on the MXene sheet surface, which contributes to the specific surface area growth. All the above mechanisms of gas-analyte detection are mainly reduced to the electron-hole model of *p*- or *n*-type semiconductors, which demonstrates the predominant role of chalcogenides when examining the sensory properties.

**Table 3:** Sensory characteristics of nanocomposite coatings containing Ti_3_C_2_T_x_/Ti_2_CT_x_ MXenes and semiconducting metal chalcogenides (MoS_2_, SnS, WSe_2_).

Gas Analyte	Concentration	Detection Conditions	Response (ΔR·100%/R_0_), %	Detection Limit	Receptor Material	Synthetic Features	Ref.
NO_2_	50 ppm	RT	60	10 ppm	Ti_3_C_2_T_x_/MoS_2_ (20 wt.%)	HF (25%), RT, 48 h, + MoS_2_: hydrothermal treatment of dispersion containing multilayer Ti_3_C_2_T_x_ MXene and MoS_2_ synthesized from MoO_3_, KSCN, and HCl, 170 °C, 8 h	[149]
NO_2_	50 ppm	RT	55	0.023 ppm	Ti_3_C_2_T_x_/TiO_2_/MoS_2_	LiF + HCl, RT, 48 h, delamination: US, 1.5 h, N_2_, +TiO_2_/MoS_2_: hydrothermal synthesis of MoS_2_ in the Na_2_MoO_4_·2H_2_O–CN_2_H_4_S reaction system on the surface of delaminated Ti_3_C_2_T_x_, 200 °C, 24 h	[151]
NO_2_	100 ppm	27 °C, RH = 43%	−173.3	500 ppb	MoS_2_/Ti_3_C_2_T_x_/TiO_2_	LiF + HCl, RT, 48 h, delamination: US, 1.5 h, N_2_, +TiO_2_/MoS_2_: hydrothermal synthesis of MoS_2_ in the Na_2_MoO_4_·2H_2_O–CN_2_H_4_S–citric acid reaction system on the surface of delaminated Ti_3_C_2_T_x_, 200 °C, 24 h	[152]
Ethanol	40 ppm	RT, RH = 5%	−12	1 ppm	Ti_3_C_2_T_x_/WSe_2_ (2 wt.%)	HF (30%), RT, 24 h, + WSe_2_: electrostatic assembly upon mixing dispersions of delaminated Ti_3_C_2_T_x_ and WSe_2_ nanoflakes having surface modified with cetyltrimethylammonium cation, 60 °C, 2 h	[153]

### 4.3. Modification of Ti_3_C_2_T_x_ and Ti_2_CT_x_ by Carbon Nanomaterials

Modification of semiconductor metal oxide-based receptor materials with carbon nanomaterials is quite common [155,156,157,158,159], primarily to reduce the detection temperature and improve the specific surface area. Studies on the efficiency of different 2D nanomaterial combinations, including MXenes (Ti_3_C_2_T_x_ or Ti_2_CT_x_) and reduced graphene oxide, for gas analyte detection with chemiresistive sensors are much less known, and the introduction of carbon nanotubes into composite materials is currently only covered for liquid sensors [160,161,162,163,164,165]. Table 4 briefly summarizes the data on the sensing properties of the composite materials, which include reduced graphene oxide (rGO) and titanium-containing MXenes.

**Figure 36:**
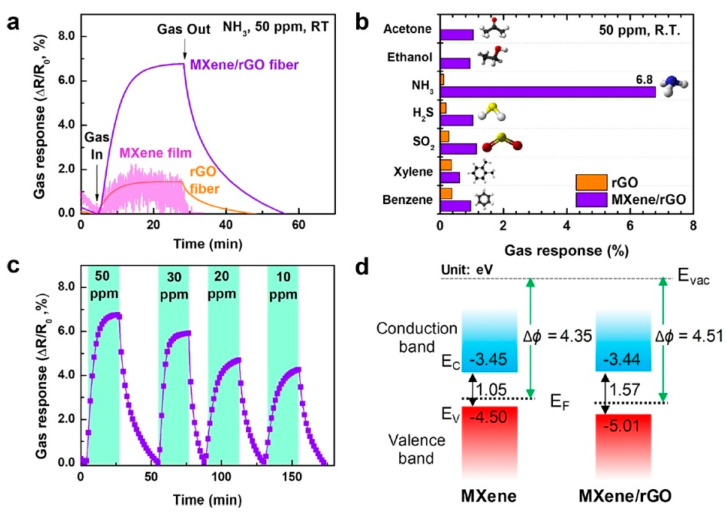
(**a**) Comparison of the gas response of MXene film, rGO fiber, and MXene/rGO hybrid fiber (40 wt.% MXene); (**b**) gas selectivity comparison of rGO fiber and MXene/rGO hybrid fiber (40 wt.% MXene) to various testing gases at concentrations of 50 ppm; (**c**) dynamic resistance response of the sensor based on MXene/rGO hybrid fiber (40 wt.% MXene) to different NH_3_ concentrations in the range of 10–50 ppm at room temperature; and (**d**) schematic energy-level diagram of MXene, and MXene/rGO (40 wt.% MXene). Reprinted with permission from [166]. Copyright 2020: American Chemical Society.

The authors of the paper [166] attempted to expand the Ti_3_C_2_T_x_ MXene band gap by the introduction of rGO with the goal of obtaining hybrid fibers of 1.2 m, excluding metal cations (Ca^2+^, Al^3+^, etc.), containing binders. The fibers were obtained by wet-spinning Ti_3_C_2_T_x_ synthesized under the influence of the LiF + HCl system and graphene oxide prepared by a modified Hummers method. For this purpose, MXene and graphene oxide dispersions in dimethylformamide were injected through a needle into a coagulating solution of n-hexane-dichloromethane to produce gel fibers, followed by thermal treatment at 200 °C. According to Raman spectroscopy data, the ratio of D-peak, at 1350 cm^−1^, corresponding to defective carbon sites, and G-peak, at 1580 cm^−1^, corresponding to sp^2^-domains, intensities was found to increase after the nanocomposite heating, confirming the conversion of GO to rGO. XPS analysis revealed that oxygen was transferred from GO to Ti_3_C_2_T_x_ MXene under thermal reduction of the composite due to the apparent potential difference. Based on the relationship between the photon energy and the optical absorption coefficient, the authors found that the nanohybrid’s band gap width has expanded from 1.05 eV to 1.57 eV. The sensing performance of the obtained nanocomposite was evaluated for ammonia, acetone, hydrogen sulfide, sulfur dioxide, xylene, and benzene at 50 ppm concentration [166]. It was found that the response to ammonia at RT was ca. 6.8%, while the response to other gases did not exceed 1%. At the same time, the nanocomposite demonstrated a ca. 4.3% response at a relatively low ammonia concentration of 10 ppm (Figure 36). The authors ascribed the ammonia selectivity to the expansion of the MXene band gap width as well as to the significant adsorption energy compared with other gases, which was confirmed by a computational DFT study referring to [87,167]. The desorption rate in this case has not been discussed.

Tran et al. synthesized a reduced graphene oxide-based nanocomposite (rGO/Ti_3_C_2_T_x_) using the conventional route of MAX-phase etching with concentrated hydrofluoric acid and the introduction of hydrazine-reduced graphene oxide under ultrasound exposure in an ice bath [168]. The derived, highly concentrated suspensions of the nanocomposite were cast onto Si substrates by dropping and dried at 60 °C. According to SEM analysis, the rGO flakes in the obtained composite cover most of the MXene sides and even enter the interlayer space; at the same time, local rGO aggregates are also found. TEM data confirmed the conclusion on the close contact of the reduced graphene oxide with the MXene plane, while SAED data indicated the Ti_3_C_2_T_x_ hexagonal structure. Based on the XPS data, the qualitative composition of the nanocomposite was confirmed, while a significant decrease in the concentration of fluoride ions on the MXene surface during the rGO introduction was observed. The gas-sensing properties of the composite-based sensor were investigated for 10–100 ppm of NO_2_. For instance, a high response of 37% at 50 ppm of NO_2_ was found. The sensor exhibited excellent repeatability of signal when exposed to five cycles of NO_2_ with no drop in response and complete recovery. NO_2_ selectivity was established when exposed to 10% methane and 100 ppm of toluene. Given that the kinetic response curves for these gases were quite poor, the authors report responses equal to 20% in the case of CH_4_ and 25% in the case of C_7_H_8_. The sensitivity to NO_2_ has been again explained by the high specific surface area of the nanocomposite, its heavy functionalization, and the efficient process of electron transfer across the rGO/Ti_3_C_2_T_x_ boundaries [168].

As already mentioned in Section 4.1.1 [104], the introduction of rGO into the Ti_3_C_2_T_x_/TiO_2_ nanocomposite, having MXene as crumpled spheres oxidized under specified conditions with a controlled number of titanium defects and derived by ultrasonic pyrolysis, leads to an increase in the NO_2_ response with the detection limit down to 10 ppb at RT. The authors show that the real reason for gas-sensing improvement is the large number of titanium atom defects caused by oxidation and the creation of Ti_3_C_2_T_x_/rGO heterojunctions. DFT calculations were performed to confirm their hypothesis. It was shown that, compared with the original Ti_3_C_2_T_x_, the adsorption energy of Ti_3_C_2_T_x_ MXene with Ti–O vacancies is significantly reduced for gas molecules, and the charge transfer goes in a more free manner. Therefore, the gas-sensing characteristics of such materials are significantly improved [104].

Wang et al. proposed the use of a ternary composite based on rGO nanosheets, nitrogen-doped Ti_3_C_2_T_x_ MXene (N-MXene), and titanium dioxide for formaldehyde detection at RT [169]. Nitrogen doping of Ti_3_C_2_T_x_ MXene synthesized with LiF/HCl was carried out by a hydrothermal method using urea as a nitrogen source at 180 °C. Under similar conditions, doping of the obtained N-MXene with titanium dioxide was carried out by a hydrothermal method using titanium tetrabutoxide and KCl solution. The introduction of rGO into the N-MXene/TiO_2_ system was performed by ultrasonic treatment of the dispersion containing N-MXene/TiO_2_ and rGO under a nitrogen atmosphere. SEM studies of the N-MXene/TiO_2_/rGO showed that the sample has a specific layered structure with a small TiO_2_ content, mainly at the edge areas. Surface functionalization was studied by IR spectroscopy. It was found that OH group-related valence vibrations of adsorbed water at 3420 cm^−1^, terminal O–H groups at 1390 cm^−1^, –NH groups at 3120 cm^−1^, and Ti–O groups at 665 cm^−1^ were detected. XRD data showed a shift of the diffraction peak from the (002) plane to low angles, from 9° to 6.7°, after N-doping of MXene, confirming that nitrogen atoms contribute to the increased interlayer distance. Under gas-sensing tests of the obtained material, it was established that there was no response to 20 ppm of formaldehyde at 20 °C in almost dry air (RH = 2.1%). However, with increasing the humidity level to the RH = 37–62% range, the response could be recorded with a low noise level [169]. The optimum RH value was found to be 54% because of the fast response and complete recovery of the sensor resistance when the target gas was removed from the cell. The average sensor response was 132% and 26%, or 20 ppm and 4 ppm of HCHO, respectively. The N-MXene/TiO_2_/rGO-based sensor showed a high selectivity to formaldehyde when compared with sulfur dioxide, carbon dioxide, ethanol, acetone, and benzene, all of which appeared at 20 ppm concentration, and 4 ppm of hydrogen sulfide; the response to competitive gases did not exceed 3.5%. The authors proposed a mechanism for increasing sensitivity to formaldehyde with increasing atmospheric humidity [169]. According to their concepts, HCHO vapors easily dissolve in water to form hydrated formaldehyde, HCHO∙H_2_O. The resulting hydrate weakens the dissociation of adsorbed water on the TiO_2_ surface. These processes lead to resistance rising because, according to the authors, the dissociation of H_2_O molecules on the metal oxide surface produces ionic-type conductive behavior. The hydration reaction is reversible due to the long and weak hydrogen bonds in HCHO∙H_2_O [169].

A synthesis method of 3D Ti_3_C_2_T_x_/rGO/SnO_2_ aerogel as a formaldehyde detector was developed by the authors in [170]. The given nanocomposite was obtained by a hydrothermal method using freshly prepared Ti_3_C_2_T_x_ under hydrofluoric acid etching and GO produced by Hummer’s method with the addition of SnCl_4_∙2H_2_O, NH_4_F, urea, and ammonium citrate. SEM and TEM images indicated the composite’s 3D structure, where Ti_3_C_2_T_x_ and rGO are represented by linked nanosheets, while SnO_2_ appears as spheres distributed on top of the sheets. The resulting material is characterized by an extremely high specific surface area of 103.2 m^2^/g, as confirmed by BET data. The sensor based on synthesized Ti_3_C_2_T_x_ /rGO/SnO_2_ was studied after exposure to 10 ppm of formaldehyde [168]. Nanocomposite was found to be an *n*-type semiconductor and exhibited a high response/recovery rate of 2.9 s and 2.2 s, which seems to be a sequence of a large number of surface-active centers accelerating the adsorption/desorption of the target gas. As the concentration of formaldehyde grew from 10 ppm to 200 ppm, the sensor response increased from ca. 55% to ca. 275%. The authors also compared the response to formaldehyde, which was found to be superior to those toward ethanol, methanol, acetone, ammonia, and toluene at the same concentration of 10 ppm. This sensor performance was explained by the high conductivity of the composite and the lowest bond dissociation energy of formaldehyde, 364 kJ/mol, in the series of gases studied, which contributes to a rapid reaction with chemisorbed oxygen on the surface. It was demonstrated that humidity vapors ranging from 20 rel.% to 60 rel.% have no significant effect on the response, but further increasing to 85 rel.% causes the response to drop by a quarter. To explain the observed sensor, the authors followed a typical detection mechanism in metal oxides dealing with redox reactions between the analyte gas and chemisorbed oxygen on the surface of a 3D composite and charge transfer into the EDL of a semiconductor. Again, the p-n-heterojunctions in this composite play a positive role in facilitating charge carrier separation and expansion of the EDL width. The high selectivity towards formaldehyde was supported by DFT calculations. It was found that the composite has the strongest affinity for HCHO [170].

**Figure 37:**
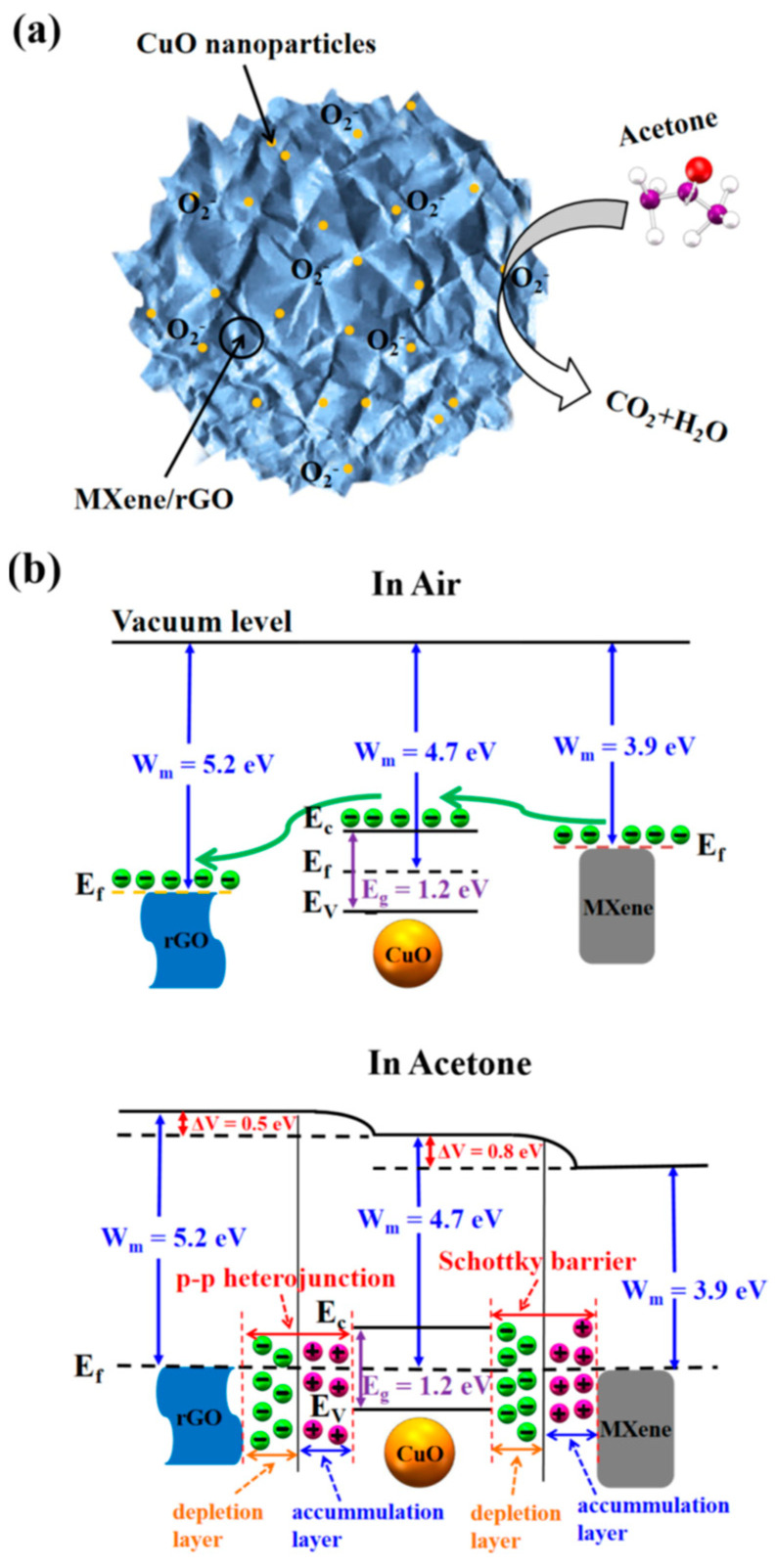
(**a**) Gas-sensing mechanism diagram of the 3D MXene/rGO/CuO aerogel to acetone gas. (**b**) Band structure diagram of the 3D MXene/rGO/CuO aerogel under different conditions. Reprinted with permission from [171]. Copyright 2021: Elsevier B.V.

Using a procedure similar to [170], a 3D Ti_3_C_2_T_x_/rGO/CuO aerogel was created [171] by one-step hydrothermal treatment of a solution containing copper chloride, graphene oxide, MXene, ammonium fluoride, urea, and sodium citrate, followed by freeze-drying. The aerogel was mixed with ethanol to form a homogeneous paste that was applied with a brush to an Al_2_O_3_ substrate equipped with Au electrodes. SEM images revealed that Ti_3_C_2_T_x_/rGO/CuO consists of a porous network similar to the original rGO with the copper oxide nanoparticles evenly distributed on its surface, while MXene sheets are placed on the rGO sheets. XRD patterns confirmed the presence of CuO and rGO plane reflexes only, which the authors attribute to an excessively low MXene concentration. Gas-sensing tests of the obtained 3D aerogel coatings showed that the maximum response value was achieved at 25 °C. High selectivity to acetone, with a response to 100 ppm concentration equal to ca. 50.9%, was argued because the response to other test gases, including benzene, thymethylamine, methanol, toluene, and triethylamine, did not exceed 15%. The authors showed that humidity significantly affected the sensor characteristics of the composite: the sensor response went gradually down from 52% to 43% with an increase in humidity from 20 rel.% to 60 rel.%. This effect is associated with the screening of surfaces by H_2_O molecules. The mechanism of acetone detection proposed by the authors (Figure 37) is fully based on the typical concepts characteristic of p-semiconductors, such as CuO. The improved gas-sensing performance is justified by a p-p-heterojunction at the rGO-CuO interface and the presence of Schottky barriers at the Ti_3_C_2_T_x_-CuO interface. Furthermore, the high response of the aerogel is explained by its large specific surface area.

Zhou et al. reported the creation of a chemiresistive gas sensor based on N-Ti_3_C_2_T_x_/PEI/rGO polymer composite, where PEI is polyethyleneimine, to detect CO_2_ in a wide range of concentrations, 4–3000 ppm at RT [172]. The methodology for the synthesis of MXene and its nitrogen doping is similar to that described in [169]. The synthesis of the composite was performed by mixing N-Ti_3_C_2_T_x_, PEI solution, and rGO under ultrasound for 2 h. SEM images of the obtained products indicated that the nitrogen-doped MXene has a laminar structure wrapped in PEI with a small number of TiO_2_ particles located predominantly at its boundaries. XRD patterns displayed a rather weak (110) reflection of Ti_3_C_2_T_x_, which the authors attributed to the covering effect of the amorphous PEI. A high degree of interaction between the polymer and N-MXene was confirmed by a strong electrostatic interaction due to different zeta potentials (PEI: +3.95 mV; N-Ti_3_C_2_T_x_: −16.7 mV). It was found that the obtained composite film had a maximum response value with a full recovery at 62 rel.% of humidity under RT conditions; when detecting 600 ppm of CO_2_, the response reached almost 9% at RH = 60%. When measuring the sensor’s response to input of other gases (SO_2_, H_2_S, HCHO, CO, NH_3_), it was confirmed to have a high selectivity to CO_2_. The authors argued that the large number of amino groups in PEI provides a stable and strong interaction with CO_2_ molecules, especially in a humid atmosphere. The proposed sensor demonstrated a superior lower limit of CO_2_ detection, equal to 8 ppm, than its commercial infrared counterpart, whose detection limit is 50 ppm. The high response in a humid atmosphere and the high selectivity of the N-Ti_3_C_2_Tx/PEI/rGO-based coating are further attributed to the proton conductivity of the composite [172]. The primary and secondary amino groups of PEI promote the acid-base reaction with the formation of carbamates, while the tertiary amines supply the CO_2_ dissolution with carbonic acid formation through a base-catalyzed hydration. This feature yields a faster recovery of CO_2_ molecules than one in the acid-base reaction due to the branched PEI structure, which inhibits the acid-base reaction due to the steric effect. In a humid atmosphere, the hydrophilic N-doped MXene is covered with H_2_O molecules to protonate the polymer, which, as a result, facilitates a higher response rate. At the same time, rGO promotes charge transfer and accumulation. Following CO_2_ exposure, the amount of free amino groups at the composite is reduced due to the noted reactions, which support lower proton mobility, resulting finally in a significant rise in resistance [172].

Summing up, it can be noted that the researchers introduced carbon materials into MXene to target the following issues:

(1) To increase the specific surface area, which directly affects the amount of sorbed analyte gas;
(2) To create p-p- and p-n-heterojunctions, which contribute to a more complete charge separation and hence a higher response;
(3) To create defects in the MXene structure under controlled oxidation conditions that promote adsorption centers for analytes;
(4) To change the prevailing conductivity type from *p*- or *n*-type to ionic with the introduction of polymer matrices.

Many authors provide DFT calculations to explain the high selectivity for a particular analyte, which confirm the adequate adsorption and desorption energies. However, they do not emphasize or take into account that simulations are performed in ideal conditions without chemisorbed oxygen on the sensor surface, which casts doubt on this method of confirming gas selectivity mechanisms, especially for the works that employ metal oxide semiconductors as dopants. Therefore, a set of synthetic and research methods for the study of the influence factors on the chemoresistance properties of composite sensors based on MXene and carbon materials needs further improvement and extension.

**Table 4:** Sensory characteristics of nanocomposite coatings containing Ti_3_C_2_T_x_/Ti_2_CT_x_ MXenes and reduced graphene oxide.

Gas Analyte	Concentration	Detection Conditions	Response (ΔR·100%/R_0_), %	Detection Limit	Receptor Material	Synthetic Features	Ref.
Ammonia	50 ppm	RT	+6.77	10 ppm	rGO/Ti_3_C_2_T_x_ (40 wt.%)	LiF + HCl, 50 °C, 24 h, +rGO: wet-spinning of composite fibers from dispersion of Ti_3_C_2_T_x_ and GO in DMF with subsequent heat treatment, 200 °C, Ar	[166]
NO_2_	50 ppm	RT	+37	10 ppm	rGO/Ti_3_C_2_T_x_ (33 wt.%)	HF (50%), 50 °C, 24 h, +rGO: ultrasonic treatment of ethanol dispersion containing Ti_3_C_2_T_x_ and rGO (hydrazine reduction), 0 °C, 40 min	[168]
NO_2_	5 ppm	RT	−19.854	10 ppb	Ti_3_C_2_T_x_/TiO_2_/rGO	Mixing of Ti_3_C_2_T_x_ and GO dispersions (mass ratio 2:1)	[104]
Formaldehyde	50 ppm	RT, RH = 54%	+325	2 ppm	N-Ti_3_C_2_T_x_/TiO_2_/rGO	LiF + HCl (6 M), RT, 2 h, nitrogen doping: hydrothermal treatment of ethanol dispersion with urea addition, 180 °C, 24 h, + TiO_2_/rGO: (1) hydrothermal treatment of «N-Ti_3_C_2_T_x_–Ti(OC_4_H_9_)_4_–KCl–C_2_H_5_OH–H_2_O» system, 180 °C, 24 h, (2) Ultrasonic treatment of suspension containing N-Ti_3_C_2_T_x_/TiO_2_ and rGO, N_2_	[169]
Formaldehyde	200 ppm	RT, RH < 30%	−275	10 ppm	Ti_3_C_2_T_x_/rGO/SnO_2_	HF (10%), RT, 6 h, + rGO/SnO_2_: hydrothermal treatment of suspension containing GO, Ti_3_C_2_T_x_, SnCl_4_∙2H_2_O, NH_4_F, urea, and ammonium citrate, 140 °C, 24 h, washing with HCl_dil_, freezing	[170]
Acetone	200 ppm	25°C, RH~20%	+50.9	10 ppm	Ti_3_C_2_T_x_/rGO/CuO	HF (10%), RT, 6 h, delamination: DMSO, US, + rGO/CuO: hydrothermal treatment of suspension containing GO, Ti_3_C_2_T_x_, CuCl_2_∙2H_2_O, NH_4_F, urea, and ammonium citrate, 140 °C, 24 h, washing with HCl_dil_, freezing, heat treatment, 300 °C, 2 h, Ar	[171]
CO_2_	600 ppm	RT, RH = 60%	+9	8 ppm	N-Ti_3_C_2_T_x_/PEI/rGO	LiF + HCl (6 M), RT, 2 h, nitrogen doping: hydrothermal treatment of ethanol dispersion with urea addition, 180 °C, 24 h, +PEI/rGO: ultrasonic treatment of an ethanol suspension containing N-Ti_3_C_2_T_x_, rGO (prepared by glucose reduction in alkaline medium), and PEI, 2 h, N_2_	[172]

### 4.4. Modifying Ti_3_C_2_T_x_ and Ti_2_CT_x_ with Noble Metal Nanoparticles

The decoration of receptor materials with noble metal nanoparticles is an effective method for the sensitization of semiconducting metal oxides [173]. There is a series of works on obtaining composite materials for chemiresistive gas sensors containing Ti_3_C_2_T_x_ MXene whose surface has been decorated with highly dispersed Pt, Pd, Ag, and Au particles. Table 5 briefly summarizes the sensory properties of these materials.

**Figure 38:**
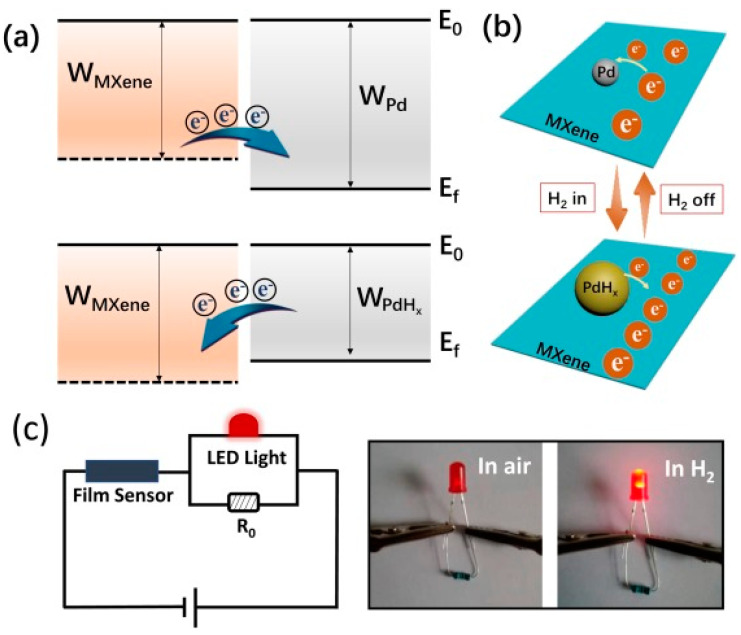
(**a**) Energy band diagrams of Pd and MXene before and after H_2_ exposure, and (**b**) the diagram of surface H_2_ adsorption and electron transfer between Pd colloidal nanoclusters (CNC) and MXene. The significantly lowered work function of PdH_x_ has driven the electron transfer back from Pd/PdHx to MXene, resulting in the electron doping of MXene and therefore the resistance decrease. (**c**) An alarm circuit design for H_2_ leaks detection using one piece of MXene@Pd CNC film sensor and LED bulb, and photographs indicating the LED bulb was turned on after H_2_ exposure. Reprinted with permission from [174]. Copyright 2020: Elsevier B.V.

Thus, Zhu and colleagues [174] considered the possibility of designing flexible and light-weight electronic devices, including high-performance H_2_ sensors operating at RT with the help of Ti_3_C_2_T_x_, MXene, and Pd nanoclusters. For this purpose, a PVP (polyvinylpyrrolidone)-stabilized suspension of palladium clusters was introduced into the dispersion of layered Ti_3_C_2_T_x_, which enabled the metal particles to fix on the MXene surface. With support from the EDX method, the mass fraction of Pd in Ti_3_C_2_T_x_/Pd films was found to be ca. 9.4%, while cross-sectional mapping ensured C and O atoms would distribute throughout the material volume but Ti, F, and Pd atoms would appear mainly on the surface. The increase in the MXene interlayer distance, as shown by the shift of (002) reflection for the Ti_3_C_2_T_x_ phase from 6.5° to 5.5° on the composite XRD pattern, indicates the introduction of Pd nanoclusters between the MXene planes. The XPS spectra proved that the palladium is partially oxidized, which is not a hindrance to the detection of hydrogen because the latter has strong reducing properties. The sensory properties of Ti_3_C_2_T_x_/Pd coatings were studied upon exposure to 0.5–40% of hydrogen in air [174]. An optimal material composition with maximum hydrogen sensitivity was found, while the pristine Ti_3_C_2_T_x_ layer yielded almost no response to H_2_. It was revealed that the output current of the Ti_3_C_2_T_x_ /Pd composite increased under hydrogen interaction to be equivalent to a decrease in resistance, indicating an *n*-type electronic conductivity. The responses to 0.5–40% of hydrogen were ca. 7.4–41.7%. The response and recovery times of the sensors were found to show a tendency to rise with increasing H_2_ concentration. The Ti_3_C_2_T_x_/Pd film exhibited excellent cyclic stability and reproducibility. The gas-sensing properties of the Ti_3_C_2_T_x_/Pd film placed on the flexible substrate were also investigated upon mechanical bending at various angles (θ = 30°, 60°, 90°, 120°), and after successive “bending-straightening” cycles from 0° to 180°. The sensor displayed good stability in terms of response and response/recovery time. Furthermore, the authors supported the long-term stability of the sensitive coating via its measuring for 20 days: the response value and response time to 4% of H_2_ were almost the same as when the experiment for analyte detection began. It is worth noting that the sensor showed an insignificant response to gases other than hydrogen, including methanol (1000 ppm), acetone (1000 ppm), and ammonia (500 ppm). These tests proved the selectivity of such a material for H_2_. The authors tried to explain the Ti_3_C_2_T_x_/Pd resistance drop upon H_2_ exposure (Figure 38) and assigned this mechanism to the formation of palladium hydrides (PdH_x_) during hydrogen adsorption, which have a lower work function of approx. 3.2 eV than that of metallic Pd, of approx. 5.3 eV, resulting in a reverse electron transfer from palladium hydride clusters to the MXene [174]. The reverse process occurs during H_2_ desorption. The authors assembled a hydrogen leak detection system with a Ti_3_C_2_T_x_/Pd film and LED lamp. The stable operation of the lamp when exposed to 4% H_2_ for tens of seconds confirms the successful H_2_ leak alarm.

Phuong Doan et al. [175] tried to solve two tasks at once: to develop a highly sensitive and selective hydrogen sensor and a solid-state hydrogen accumulator. For this purpose, a Na_2_PdCl_4_ solution with polyvinylpyrrolidone was mixed with an ethylene glycol suspension of multilayer Ti_3_C_2_T_x_ MXene obtained by Ti_3_AlC_2_ MAX-phase etching with concentrated hydrofluoric acid. Then, the Pd nanoparticles were synthesized via heating the resulting reaction system at 160 °C for 2 h. According to the XPS analysis, it was found that the surface of the multilayer MXene after the palladium decoration was functionalized with –OH or –O groups. Based on SEM data, the authors identified the best sample in terms of density and palladium nanoparticle distribution, suitable for gas-sensing studies. For the selected Ti_3_C_2_T_x_/Pd sample, satisfactory responses to 10–100 ppm of H_2_ are noted; in particular, the chemiresistive response to 100 ppm of H_2_ was 56%.

The sensor is reported to demonstrate distinct responses even after 90 days of storage in air. Selectivity was tested against 100 ppm of methane, ammonia, and nitrogen dioxide. The response to all these analytes did not exceed 5%. It should be noted that no baseline recovery was demonstrated after NO_2_ exposure. The authors explain the good sensor selectivity versus H_2_ by the creation of palladium hydrides, whose particles can contribute to hydrogen atoms’ delivery to the neighboring MXene planes through the spill-over mechanism with the formation of TiH_2_. Meanwhile, such a functionalization leads to increased sample resistance. This hydrogen transfer is also enhanced by surface oxygen-containing groups. When discussing this issue, the authors refer to the data in [176] indicating that hydrogen atom diffusion is intensified when the concentration of oxygen groups grows.

**Figure 39:**
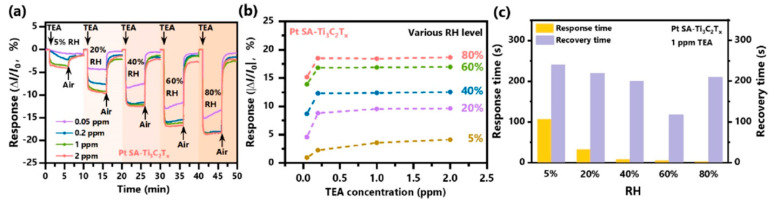
(**a**) Sensor responses to triethylamine (TEA) (0.05–2 ppm) under various RH (5 to 80%). (**b**) Plots of sensor response vs. TEA concentration from (**a**). (**c**) Response and recovery times of the Ti_3_C_2_T_x_/Pt sensor toward 1 ppm TEA at various RHs. Reprinted with permission from [177]. Copyright 2022: American Chemical Society.

The purpose of another work [177] was to study the gas-sensing characteristics of VOC sensors based on field-effect transistors employing Ti_3_C_2_T_x_/Pt composite as receptor material. To decorate MXene planes with single platinum atoms, a solution of H_2_PtCl_6_·6H_2_O was added to its dispersion, followed by stirring the reaction system in an Ar atmosphere for 30 min. The authors point out that the process of MXene delamination supports the appearance of titanium vacancies on the Ti_3_C_2_T_x_ nanosheet surface, which serve as active centers for anchoring single Pt atoms due to their strong ability to capture alternative atoms and good reducing potential. It was shown that the Ti_3_C_2_T_x_/Pt sample obtained by such a method had a high response to 10 ppm of triethylamine. However, rather long response and recovery times were observed, suggesting a strong interaction between the analyte molecules and the material surface. Tests revealed that the sensor resistor baseline shifts to higher values by 55% and 172% after 20 days of exposure to 20 rel.% and 80 rel.% of surrounding humidity, and the response goes down by 60% and 89%, respectively (Figure 39). On the contrary, exposure to dry air (RH < 5%) almost does not change the resistance, which indicates that the coating is stable enough in a low-humidity environment. The selectivity was studied with respect to 10 ppm of analytes of different chemical natures, including acetone, ethanol, formaldehyde, benzene, hydrogen sulfide, ammonia, carbon monoxide, and dioxide. It is demonstrated that the triethylamine response is three times higher when compared with that of other gases. Overall, following the results given in [177], it can be stated that single Pt atom doping enhances the response of Ti_3_C_2_T_x_ MXene to triethylamine vapor. To explain the detection mechanism, the authors assume that triethylamine is oxidized at the material surface while interacting with surface-adsorbed oxygen. As a result, additional electrons are released, according to Reaction (26). Because the Mxene is considered a *p*-type conductor, these surface reactions lead to a lower carrier density of holes and reduced conductivity.

2(C_2_H_5_)_3_N + 43 O^−^_(ads)_ → 12CO_2_ + 2NO_2_ + 15H_2_O + 43 ē (26)

The enhancement of the response under introducing single Pt atoms matures from, according to the authors, the effect of chemical sensitization and advancing the adsorption of analyte/oxygen molecules.

The study by Xu and colleagues [178] was aimed at designing a hydrogen sulfide sensor capable of operating under real humidity conditions with the help of delaminated Ti_3_C_2_T_x_ modified with Ag nanoparticles. To synthesize the Ti_3_C_2_T_x_/Ag composite, a silver nitrate solution was introduced into the few-layer MXene dispersion, followed by ultrasonic treatment. TEM analysis revealed that silver nanoparticles have an average size in the range of 28.2–30.8 nm. The XRD data showed that the decoration of MXene by silver nanoparticles causes an obvious oxidation of the MXene surface; the shift of the (002) plane reflection from 7.4° to 6.3° indicates widening the interlayer space. D and G bands of graphitized carbon have been found in the Raman spectra. Measurement of the CVC (current-voltage characteristics) showed that higher additions of silver result in a reduction of the sample’s conductivity. The authors suggested that this phenomenon belonged to the oxidation of MXene with the reduction of silver on its surface. All samples showed *p*-type semiconductor characteristics. Studies with exposure to hydrogen sulfide revealed that the most sensitive sample of Ti_3_C_2_T_x_/Ag yields the chemiresistive response to 10 ppm of H_2_S equal to 60%, which is more than 10 times higher than that of undecorated MXene [178]. The study of selectivity for 1 ppm of NO_2_, SO_2_, H_2_, acetone, ethanol, CO, ammonia, and formaldehyde showed that the Ag addition promotes the detection of all the gases under test. The authors hypothesized that the water molecules being adsorbed on the surface would reduce the number of charge carriers at the MXene surface, which would additionally cause the response to advance [178]. The observed resistivity growth in the detection of all test analytes, or *p*-type response, is explained by the fact that (1) gas adsorption prevents a charge carrier transfer and reduces carrier density, and (2) gas adsorption might increase the interlayer distance and thereby prevent an interlayer electron transfer. The enhanced response of the Ti_3_C_2_T_x_/Ag sample to hydrogen sulfide is due primarily to the chemical sensitization of MXene by silver nanoparticles and the reduction of partially oxidized silver with the release of electrons, their recombination with holes, and a corresponding increase in resistance.

Wu et al. [179] investigated the effect of Ti_3_C_2_T_x_ decoration by silver on its gas sensitivity towards ammonia. The synthesis of Ti_3_C_2_T_x_/Ag composite was performed by the self-reduction of AgNO_3_ in the solvent over the reducing MXene surface via the introduction of the silver nitrate solution into the dispersion of multilayer MXene, to be followed by stirring for 30 min. The sensors were printed using an electrohydrodynamic jet printer with Ti_3_C_2_T_x_/Ag-based functional inks. It was found that the Ti_3_C_2_T_x_/Ag and multilayer Ti_3_C_2_T_x_ sensor materials were more sensitive to NH_3_ after tetramethylammonium hydroxide intercalation than the few-layer MXene-based sensors. The authors explain this with three reasons [179]:

− XPS and IR results showed that these materials are characterized by a large number of defects considered to be effective adsorption centers;
− The increased content of intercalated water, which has an essential role in ammonia adsorption due to its strong affinity, was proven experimentally for these samples and will be further confirmed with the help of DFT calculations;
− These materials had a hierarchical, accordion-like structure, which is characterized by a high porosity and a specific surface area.

A gold nanoparticle-decorated multilayer Ti_3_C_2_T_x_ MXene was considered by the authors in their study [180] as a receptor material for the detection of low formaldehyde concentrations. In this case, the Ti_3_C_2_T_x_ sample synthesized with a mixture of lithium fluoride and hydrochloric acid was ultrasonically treated and then mixed with HAuCl_4_·3H_2_O for the Au^3+^ reduction reaction and the formation of nanoclusters on the MXene surface. SEM images revealed that Au nanoparticles, about 30 nm in diameter, were uniformly distributed on the Ti_3_C_2_T_x_ sheets. The exceptional Ti_3_C_2_T_x_-Au selectivity towards oxygen-containing organic compounds (ethanol, formaldehyde, and acetone) compared with hydrocarbon and aromatic analytes (toluene, hexane, and ethylene) was established and can be explained by dipole scattering from methyl groups that reduces the overall electron transfer and decreases the response. Among all oxygen-containing analytes, formaldehyde yielded the highest chemiresistive response; in particular, the response to 20 ppm of its concentration was 3%. The response to 200 ppm of water vapor, equal to 1.28%, was found to be significantly lower compared with that to 20 ppm of HCHO, indicating quite selective formaldehyde detection.

In summary, it can be concluded that the decoration of MXenes with noble metal nanoparticles/clusters considerably advances the sensitivity to the analyte gases, for which the latter exhibit a high catalytic activity. Most researchers connect this fact with: (1) the emerging spill-over effect; (2) increasing the number of adsorption centers for oxygen molecules and analyte gases; and (3) intercalating noble metal atoms between MXene sheets that promote an inter-layer electron transfer. Most researchers also decorated the MXene surface with noble metal atoms in situ, which additionally caused a reduction in conductivity as a result of MXene surface partial oxidation accompanying the appearance of heterojunctions. However, this aspect of the detection mechanism was not considered in any of the given articles. Thus, the understanding of the synergistic effect caused by the noble metal introduction into the MXene-based composite structure and contributing to the improved sensing characteristics of their sensors is at an early stage and needs further exploration.

**Table 5:** Sensory characteristics of coatings based on nanocomposites containing Ti_3_C_2_T_x_/Ti_2_CT_x_ MXenes and noble metal nanoparticles.

Gas Analyte	Concentration	Detection Conditions	Response (ΔR·100%/R_0_), %	Detection Limit	Receptor Material	Synthetic Features	Ref.
H_2_	0.5%	RT	−7.4 *	0.5%	Ti_2_CT_x_/Pd	LiF + HCl (9M), 40 °C, 24 h, delamination: US, 30 min; +Pd: mixing dispersions of MXene and Pd nanoclusters, separation by filtration	[174]
H_2_	100 ppm	RT	+56	10 ppm	Ti_3_C_2_T_x_/Pd	HF (50%), 50 °C, 24 h, +Pd: ML-Ti_3_C_2_T_x_ suspension in ethylene glycol was mixed with Na_2_PdCl_4_ solution with polyvinylpyrrolidone, heating at 160 °C, 2 h	[175]
Triethylamine	10 ppm	RT	+6 *	0.05 ppm	Ti_3_C_2_T_x_/Pt	+Pt: H_2_PtCl_6_·6H_2_O solution was added to the few-layer Ti_3_C_2_T_x_ MXene dispersion, stirring for 30 min, Ar	[177]
H_2_S	1 ppm	RT	+6 *	50 ppb	Ti_3_C_2_T_x_/Ag	+Ag: silver nitrate solution was introduced into the few-layer Ti_3_C_2_T_x_ dispersion, followed by ultrasonic treatment	[178]
Formaldehyde	20 ppm	RT	+3	1 ppm	Ti_3_C_2_T_x_/Au	LiF + HCl (9M), RT, 24 h, delamination: US, 1 h; +Au: HAuCl_4_ solution was added to the few-layer Ti_3_C_2_T_x_ dispersion, stirring	[180]

* S = (I − I_0_)/I_0_ × 100 (%) [174,177,178].

### 4.5. Modifying Ti_3_C_2_T_x_ and Ti_2_CT_x_ with Polymers

The development of composite materials containing MXenes and polymers is quite common, primarily for obtaining materials for liquid sensors. This is justified by the necessity to promote porosity and to design hierarchical structures, which are extremely important for MXene-based materials. These issues were thoroughly discussed in another review [181]. In addition, the introduction of polymer components leads to high elasticity and durability of the MXene-based layers, which are required for their practical implementation in flexible Internet-of-Things units. The presence of certain functional groups, acidic or basic, on the polymer surface improves the selectivity of the sensors for specific gaseous analytes.

The number of studies on the fabrication and analysis of chemiresistive gas sensors employing MXenes and polymers is not high. Most of them are mainly aimed at detecting ammonia [182,183,184,185,186,187], methanol [188], and NO_x_ [189,190]. Almost all of the composites under study were developed with conductive polymers, such as polyaniline [182,183], PEDOT:PSS [184,185,188], polypyrrole [186], and polyethyleneimine [172,190].

Thus, a high-performance ammonia sensor based on a composite of hollow polyaniline nanospheres with Ti_3_C_2_T_x_ (2–25 wt%) MXene, which was deposited directly on a flexible PET substrate during polymerization, is reported in [182]. The response, ΔR/R_0_, to 10 ppm NH_3_ of the PANI/Ti_3_C_2_T_x_ composite with an optimum MXene content of 15 wt.% was found to be 3.7 at room temperature. The degradation of the material under a 20° cyclic bending, which leads to a decrease in the response value, is only noticeable after 500 cycles.

In the paper of Li et al. [183], a flexible hybrid ammonia sensor was developed using the PANI/Ti_3_C_2_T_x_ composite for agricultural tasks. It is shown that the material exhibits an appropriate performance even at a relative humidity of 20–80% in the practically important temperature range of 10–40 °C. The authors gave an explanation of the high gas-sensing properties due to the formation of the Schottky barrier in the material under study and the improvement of the polymer protonation degree.

For the PEDOT:PSS/Ti_3_C_2_T_x_ composite, the sensitivity to ammonia [184,185] and to such an important VOC as methanol [188] was also observed. Nitrogen doping and partial MXene oxidation were found to increase the interlayer distance and consequently improve the NH_3_ detection kinetic parameters of the 100 wt.% N-Ti_3_C_2_T_x_–50 wt.% PEDOT:PSS composite [184]. At a mass ratio of PEDOT:PSS/Ti_3_C_2_T_x_ equal to 4:1, a high sensitivity of the hybrid material at room temperature to 300 ppm methanol was also shown as a result of the increased interlayer distance in MXene; for comparison, the response to 300 ppm acetone and ethanol is more than 5 times lower.

For the sensor based on a composite of polypyrrole (PPy) and Ti_3_C_2_T_x_, derived by polymerization in the presence of MXene plates, it is also reported to have enhanced sensitivity to ammonia, making it promising for implementation in the Internet of Things (IoT) [186]. The authors attribute this primarily to advancing the specific surface area, the high electron mobility of MXene, and the formation of hydrogen bonding between the polymer and Ti_3_C_2_T_x_ functional groups.

Thus, the use of composite materials based on Ti_3_C_2_T_x_ MXene with conductive polymers allowed one to enhance the observed responses not only by making materials with a developed surface, which provides a higher number of adsorption centers and facilitates gas access to the MXene surface, but also by generating Schottky barriers at the components’ interfaces. Moreover, a number of experiments have shown a high influence of ionic conductivity on the detection mechanism in a humid atmosphere, which is characteristic for electrically conductive polymers. This can be extremely promising for the manufacturing of sensors aimed at detecting diseases taking place in vivo through exhaled breath because this environment is highly enriched with humidity vapors.

## 5. Conclusions

Analysis of publications on the design of chemiresistive sensor materials based on carbide MXene phases, Ti_3_C_2_T_x_ and Ti_2_CT_x_, shows a high potential of these materials for sensors operated at RT and indicates extensive R&D efforts applied by the research community in this field to level the unfavorable properties of MXenes as receptor materials. Among others, the low stability against oxidation and the extreme sensitivity to H_2_O vapors can be partially compensated in these MXene structures by a chemical modification of their surface with bulk organic fragments, often of a hydrophobic nature such as sodium ascorbate, trimethylacetic acid anhydride, or perfluoroalkyl silane. In addition to creating steric hindrances for the diffusion of water molecules to the MXene surface, this also allows one to expand the interlayer space, i.e., to enlarge (i) the diffusion space for analyte gases, (ii) the specific surface area, and (iii) the number of active adsorption centers, and consequently to improve the response and kinetic characteristics. Doping MXenes with heteroatoms, such as nitrogen or sulfur, as well as replacing functional groups with –Cl, –Se, etc., also provides an opportunity to extend the distance between MXene 2D layers, which gives some options for detection selectivity because of the geometry factor.

The approaches used to develop MXene composites with carbon nanomaterials, polymers, noble metal nanoparticles, and especially semiconducting metal oxides are shown to be very effective. In these hybrid materials, the operation temperature is reduced almost to RT. That is a drastic difference from, say, metal oxide-based gas sensors, which are currently the most commercialized ones. Moreover, the response value and response/recovery times of pristine MXenes are substantially improved via enhancing the material porosity and appearing heterojunctions at the interface of involved components. In the case of MXenes decorated with a minor amount of metal oxides, the latter atoms could embed into the inter-layer space and be not just localized on the surface of MXenes that facilitates its access to analyte molecules from a gas phase, increasing the number of adsorption centers.

The detection mechanisms suggested by the authors for Mxene-derived composites are not always uncontroversial. However, in recent years, the following factors have appeared to be among the primary reasons explaining the enhancement of their sensory properties:

(1) An increase in the specific surface area of nanocomposites, thus raising the diffusion rate of gases and advancing the number of adsorption centers;
(2) Retention of polar functional groups (–O, –OH, –F, etc.) on the MXene surface within the composites and the appearance of titanium defects that improve gas adsorption, in particular for analytes capable of forming hydrogen bonds;
(3) High electric conductivity and charge carrier mobility, typical for MXenes, which allow one to raise in some cases the energy efficiency of sensors based on composites with semiconductor metal oxides;
(4) Composed of heterojunctions that support an effective separation of charge carriers at the interfaces and improve the chemiresistive response;
(5) Growing the number of dissociative adsorption centers with the introduction of noble metals, which exhibit a high catalytic activity in various surface reactions.

One of the most effective ways to reduce the response/recovery time is UV irradiation, optionally pulsed, to promote primarily analyte desorption, thereby accelerating this process. Still, in some composites such as Ti_3_C_2_T_x_/TiO_2_ and Ti_3_C_2_T_x_/ZnO, the UV-activation also improves sensitivity and reduces the detection limit to the ppb range of analyte concentrations.

Despite the observed progress in the development of Ti_3_C_2_T_x_/Ti_2_CT_x_-based receptor materials for RT chemiresistive gas sensors, there are some challenges, common to all sensor materials and specific to MXenes:

(1) High sensitivity to humidity hinders the detection of other analyte gases;
(2) Urgent need to detect individual components in complex gas atmospheres, i.e., under interreference with the presence of other gases that facilitate a sensor response;
(3) High, uncontrollable ability of MXenes to reduce when synthesizing gas sensor nanocomposites.

One of the promising areas where Ti_3_C_2_T_x_- and Ti_2_CT_x_-based materials can be widely applied is the development of multisensor units [191] that allow one to distinguish even chemically akin analytes due to the mathematical signal processing of vector signals. A combination of printed technologies [192] for applying sensor coatings to local miniaturized areas of the multisensor and flexible substrates [193] convenient for embedding gas sensors into portable electronics can be extremely beneficial for this purpose.

## Data Availability

Not applicable.
